# Shapeshifting exsolved FeNi bimetallic nanostructures as catalytic switchers during the CO_2_-mediated ethane conversion

**DOI:** 10.1038/s41467-026-71282-6

**Published:** 2026-04-13

**Authors:** Filippo Colombo, Anastasios I. Tsiotsias, DongHwan Oh, Luca Nodari, Georgios I. Siakavelas, Linda Joseph, Xiao Sun, Nikolaos D. Charisou, WooChul Jung, Maria Goula, Simone Mascotto

**Affiliations:** 1 Institute of Inorganic and Applied Chemistry, University of Hamburg, Hamburg, Germany; 2 Department of Chemical Engineering, University of Western Macedonia, Kozani, Greece; 3 Department of Material Science and Engineering, Korean Advanced Institute of Science and Technology, Daejeon, Republic of Korea; 4 Department of Chemical and Biomolecular Engineering (BK21 Four), Korea Advanced Institute of Science and Technology (KAIST), Daejeon, Republic of Korea; 5 Dipartimento di Scienze Chimiche, Università degli Studi di Padova, Padova, Italy; 6 Institute of Condensed Matter Chemistry and Technologies for Energy, National Research Council. C.so Stati Uniti 4, Padova, Italy; 7 Institute of Integrated Natural Science, University of Koblenz, Koblenz, Germany; 8 Deutsches Elektronen-Synchrotron, Hamburg, Germany; 9 Department of Materials Science and Engineering, Seoul National University, Seoul, Republic of Korea; 10 Research Institute of Advanced Materials, Seoul National University, Seoul, Republic of Korea; 11 Centre for Research & Technology Hellas (CERTH), Chemical Process and Energy Resources Institute (CPERI), Maroussi, Greece; 12 School of Science and Technology, Hellenic Open University, Patras, Greece

**Keywords:** Catalyst synthesis, Nanoparticles, Heterogeneous catalysis

## Abstract

In the era of the energy transition, the development of sustainable, high-performance, and multifunctional catalysts that adapt to complex catalytic processes is essential. Here, we report shapeshifting bimetallic iron–nickel catalysts developed via an exsolution strategy for carbon dioxide–mediated ethane conversion. By controlling the reduction temperature of a perovskite host, either alloyed iron–nickel nanoparticles or oxide–alloy core–shell nanoparticles are selectively formed. Oxidative regeneration of the perovskite enables reversible interconversion between these distinct nanostructures within the same parent material. As a result, the catalyst exhibits switchable selectivity between ethane dry reforming and carbon dioxide–assisted oxidative dehydrogenation while maintaining high stability. Repeated redox cycling confirms that the structural transformation and catalytic performance are largely reversible. These results demonstrate that exsolution provides a robust platform for designing regenerable catalysts with deliberately tunable and switchable catalytic states.

## Abstract (web summary)

The development of sustainable, high-performance, and multifunctional catalysts capable of adapting to complex catalytic processes is critical in the era of energy transition. Here, the authors report shapeshifting bimetallic Fe–Ni catalysts developed via an exsolution strategy for CO_2_-mediated ethane conversion, where the catalyst structure can be reversibly transformed and regenerated, enabling deliberate access to ideal catalytic states under reaction-relevant conditions.

## Introduction

The catalyst industry generated an estimated economic value of approximately USD 32 billion in 2024, with heterogeneous catalysts accounting for 71% of the market. The expanding oil and gas sector is expected to drive further catalyst demand in the coming years. Additionally, the growing focus on green/low-carbon technologies and the increasing energy needs have made catalysts crucial in refining processes such as reforming, (de-)hydrogenation, and cracking.

As the world moves towards reducing CO_2_ emissions, the energy transition not only involves new technologies but also a substantial increase in the demand for specific materials. This regards particularly critical minerals like cobalt and noble metals, fundamental in heterogeneous catalysis, which need to be substituted with more abundant elements such as iron. For this reason, the energy transition is deeply intertwined with a material transition. In the field of heterogeneous catalysis, this transformation includes increasing investments from industries in catalyst recycling and substantial innovation in the development of novel catalytic converters with high performance.

In recent years, metal exsolution from doped metal oxide matrices stood out as a highly promising approach for the design of innovative and sustainable catalyst materials^1^ with improved performance^2^, lifetime^3^, and recyclability^4,5^. Understanding the underlying mechanistic processes, such as dopant diffusion and reduction, as well as nanoparticle nucleation and growth, represents the first prerogative for the development of exsolved materials for the future catalyst market.

Among the exsolved catalysts, the design of multimetallic nanoparticles attracts a lot of attention^6,7^. It is well known that by tuning the structure and composition of bimetallic nanoparticles, the catalytic performance, and especially the selectivity, can be largely improved^8,9^. The development of iron-based multimetallic nanoparticles is currently dominating the scientific literature because this element is both the second most abundant metal on Earth, and offers high catalytic activity due to its rich redox chemistry. The rather electropositive character of Fe compared to the other metals forming the nanoalloy (e.g., Ni, Cu, and Co) is also responsible for structural and compositional changes of the nanoparticles during catalytic operation, which in turn influence the performance of the catalyst. For example, Oh et al. discovered a rocking-chair behavior of Fe species in trimetallic Fe–Co–Ni exsolved nanoalloys during chemical looping reforming coupled with CO_2_ splitting. They found that Fe species move back and forth between the inside and the outside of the host oxide matrix, i.e., as Fe cations in the oxide lattice and as part of the metallic alloy, respectively^6^. The group around Chenghao Yang and Meilin Liu showed that iron nanoalloys exsolved at high temperature (*T* > 800 °C) form a core-shell M-Fe@FeO_*x*_ structure (M = Ni, Cu) with the highly oxygen-defective FeO_*x*_ layer playing a fundamental role in the CO_2_ adsorption, dissociation, and reduction during high-temperature CO_2_ electrolysis^10,11^. In a similar work, the formation of exsolved core-shell RuFe@FeO_*x*_ nanoparticles was observed, which were shown to possess high electrocatalytic activity, with the FeO_*x*_ phase being responsible for the adsorption and conversion of the CO_2_^12^. The formation of such heterostructures appears to occur only at high temperatures, as for low-temperature exsolution (*T* < 700 °C), only nanoparticles with pure metallic surface are detected^8,13^. A common application for this variation between metallic alloy surface and M-Fe@FeO_*x*_ heterostructure is for the reaction between ethane and CO_2_^14^. This reaction has gained significant popularity in recent years, as a means to transform the underutilized ethane from shale gas and the waste CO_2_ captured from emission sources into value-added chemicals^15^. It mainly follows two different pathways, the CO_2_-assisted oxidative dehydrogenation of ethane (1-ODH) and the dry reforming of ethane (2-DER):

1 $${{{\rm{C}}}}_{2}{{{\rm{H}}}}_{6}+{{{\rm{CO}}}}_{2}\to {{{\rm{C}}}}_{2}{{{\rm{H}}}}_{4}+{{\rm{CO}}}+{{{\rm{H}}}}_{2}{{\rm{O}}}\,({{\rm{ODH}}})$$

2 $${{{\rm{C}}}}_{2}\,{{{\rm{H}}}}_{6}+2{{{\rm{CO}}}}_{2}\to 4{{\rm{CO}}}+3{{{\rm{H}}}}_{2}({{\rm{DER}}})$$

Reaction (1) is undoubtedly more valuable, as it leads to the production of ethylene, a valuable petrochemical feedstock, with a reduced carbon footprint. Reaction (2), on the other hand, can also be utilized to produce synthesis gas (H_2_ and CO mixture), which can be used in subsequent Fischer–Tropsch synthesis^15^.

For both of these reactions, bimetallic Fe–Ni catalysts have received remarkable research attention in recent years^14,16–22^ First, they avoid the use of precious metals (e.g., Pt, Pd), or toxic Cr, and they have also demonstrated an interesting ability to alter the reaction selectivity via the tuning of specific material characteristics (e.g., Fe:Ni molar ratio, metal loading, support nature and structure). Xie et al.^16^. highlighted that this selectivity control in bimetallic catalysts can be influenced by the surface chemistry and various structural transformations (e.g., alloying or oxygen-induced segregation), with the metallic (alloy) surface favoring C–C bond scission and the dry reforming selectivity, and the inverse M’O_*x*_/M (in our case FeO_*x*_/Fe–Ni) interface favoring C–H bond scission and ethane dehydrogenation^14^. In our previous work, we showed that Fe-based bimetallic exsolved heterostructures can possess a metallic (alloy) or an oxidized surface, influencing the catalytic activity and selectivity during the CO_2_-assisted ethane conversion^8^. In general, although highly interesting, this selectivity control between ethane dehydrogenation and ethane reforming could thus far be obtained by using distinct catalyst materials.

Exploiting the unique properties of exsolved nanoparticles to be influenced by the exsolution treatment conditions, as well as to be reincorporated back into the parental oxide matrix upon oxidative treatment, we propose herein an innovative strategy to prepare single exsolved bimetallic catalysts with tunable and switchable catalytic performance. La_0.4_Sr_0.4_Ti_0.6_Fe_0.35_Ni_0.05_O_3-δ_ (LSTFN2) with 20 at.% of A-site deficiency is chosen as the parental oxide matrix. The temperature-dependent exsolution behavior of FeNi nanoparticles is investigated by combining different complementary analysis techniques, and a formation mechanism for the high-temperature formation of the FeO_*x*_ shell layer is proposed. The shapeshifting from low-temperature (*T* = 400 °C) exsolved FeNi nanoparticles to high-temperature (*T* = 850 °C) exsolved FeO_*x*_@FeNi heterostructures mediated by the oxidative regeneration of the oxide matrix is carefully addressed, and we discover that it is a quasi-reversible process. The structural change is also accompanied by a corresponding switch in the catalytic performance/selectivity during the CO_2_-mediated ethane conversion.

Specifically, we show herein that the catalytic performance/selectivity can be switched between CO_2_-assisted oxidative dehydrogenation of ethane and dry reforming of ethane by simply altering the exsolution treatment conditions on a single multifunctional perovskite material. The material exsolved at 400 °C shows approx. 78% selectivity for reforming, which is switched to approx. 75% selectivity for dehydrogenation when exsolved at 850 °C instead. In addition to this, the shapeshifted/regenerated catalysts (following re-oxidation and re-exsolution) are also tested, and the selectivity switch in this case was also achieved to a great extent. Similar results were replicated via in situ regeneration (oxidation/reduction) treatments during a multi-cyclic operation.

This research, therefore, shows that by tuning the exsolution conditions and exploiting the regeneration of the oxide parental matrix, the exsolution of bimetallic structures can be tailored for the design of ideal nanomaterials with switching functional properties. These findings, obtained by a careful assessment of the chemical and structural changes of the materials, open new prospects for the use of exsolution as an innovative design strategy for advancing material transitions in energy applications and the development of smart materials.

## Results and discussion

### Parental material

**Fig. 1:**
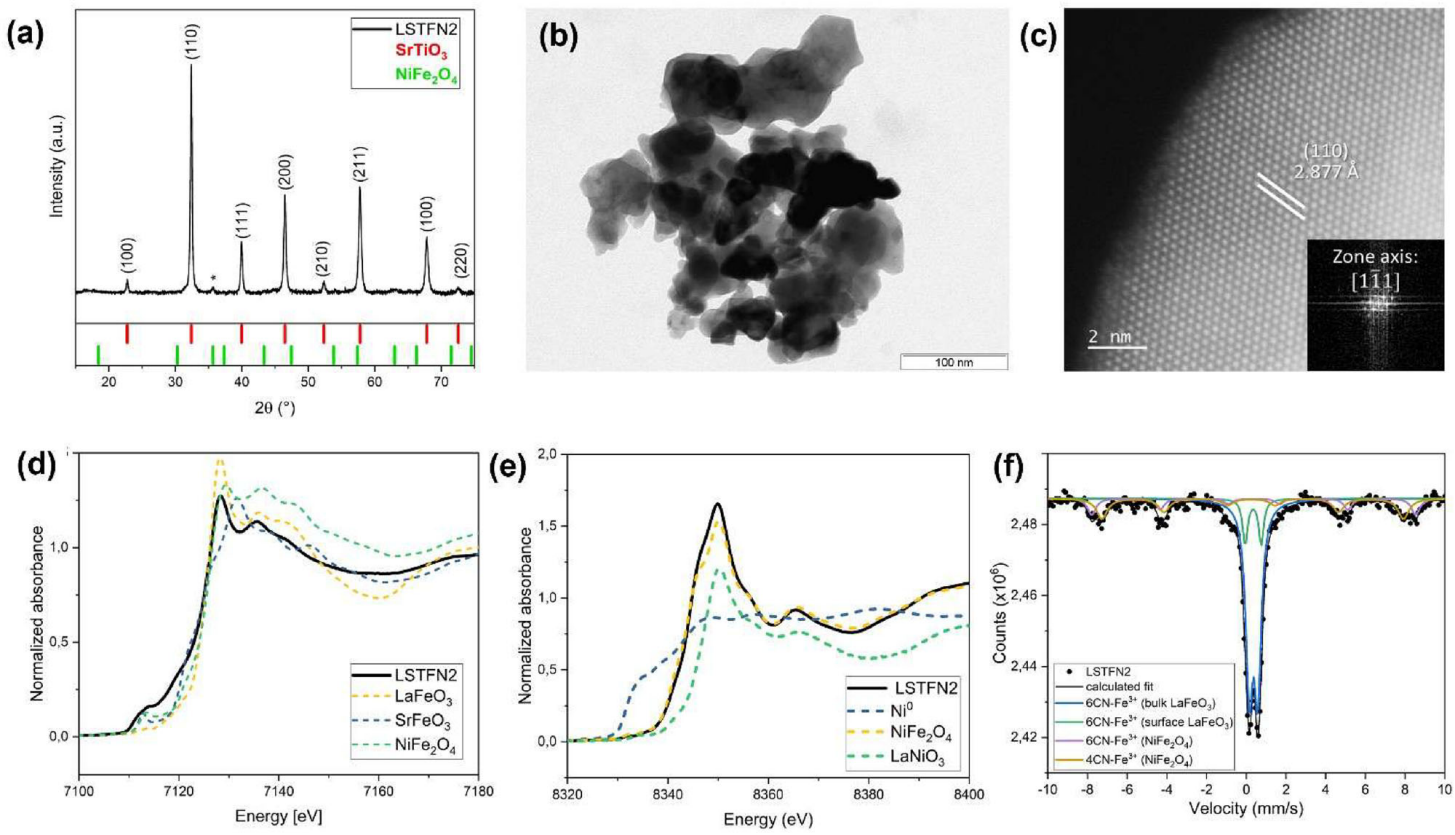
Structural characterization of the synthesized host scaffold. **a** Powder diffractogram of the LSTFN2 material. **b** TEM picture of the as-synthesized material. **c** HRTEM images of as-synthesized LSTFN2, with detail of the lattice spacing calculation. **d** XANES spectra of the Fe K-edge of the as-synthesized LSTFN2 material and different standards used for a qualitative comparison, namely SrFeO_3_, LaFeO_3_, NiFe_2_O_4_. **e** XANES spectrum of the Ni K-edge for the as-synthesized LSTFN2 material, together with Ni^0^, LaNiO_3_ and NiFe_2_O_4_ standards. **f** Mössbauer spectrum of the as-synthesized LSTFN2 material, with related attributions.

The LSTFN2 material shows a main phase with the ${Pm}\bar{3}m$ cubic space group, typical of SrTiO_3_ (PDF Nr. 01-079-0175), as already observed. Moreover, a minor spinel phase ascribed to NiFe_2_O_4_ (PDF Nr. 00-034-0641) can be noticed (Fig. 1a). Its formation can be related to a partial phase reconstruction, as a consequence of the high A-site deficiency during the high-temperature treatment. The crystallite size and the lattice parameter of the main perovskite phase are calculated to be 45 nm and 3.910 Å, respectively.

LSTFN2 is characterized by a fair specific surface area (SSA) of 15 m^2^/g determined by nitrogen physisorption (Table 1 and Supplementary Fig. 1a). The formation of a quite porous morphology is also verified by transmission electron microscopy (TEM, Fig. 1b) analysis, which evidences the presence of a nanostructured material with intraparticle porosity. Figure 1c shows an atomically resolved TEM image of LSTFN2, presenting cubic perovskite with a d-spacing of 2.877 Å in the (110) plane, which matches well with the one obtained by XRD (*d*_*110_XRD*_ = 2.758 Å). All metal elements are homogeneously occupying the bright cation sites without any segregated parts, which implies the successful synthesis of the scaffold without any significant amount of secondary phase formation. In addition, energy dispersive X-ray spectroscopy (EDX) from TEM measurement was performed to investigate the elemental distribution. All the cations are homogeneously distributed within the oxide structure (Supplementary Fig. 1b, c), and the calculated atomic percentages lead to an experimental composition very close to the nominal one, La_0.41_Sr_0.39_Ti_0.68_Fe_0.27_Ni_0.05_O_3-δ_, thus showing that, although present, the spinel side phase is marginal to the perovskite one.

**Table 1:** Lattice parameter (*a*) obtained from XRD, SSA obtained with the BET method, Ni (^Ni^E_K_) and Fe (^Fe^E_K_) K-edge energy from XANES, and relative amount in % of the iron species from Mössbauer spectroscopy

Sample	*a* [Å]	SSA [m^2^/g]	^Ni^E_K_ [eV]	^Fe^E_K_[eV]	Fe in ABO_3_	NiFe_2_O_4_	*γ*-Fe_2_O_3_	FeO	Fe^0^
LSTFN2	3.903	15	8344.2	7126.4	68	32		-	-
R400	3.899	13	8344.8	7126.7	57	28		-	15
R650	3.898	-	8345.5	7126.5	60			5	35
R850	3.916	14	8344.7	7124.3	28			29	43
R400-O	3.899	-	8344.5	7126.5	90		10		
R850-O	3.898	-	8344.5	7126.5	84		16		
R400-O-R850	3.906	10	8344.9	7124.0	35			25	40
R850-O-R400	3.900	10	8344.7	7126.4	85		10		4

More precise information on the oxidation state and local structure of the metals, and particularly on the Ni and Fe species, was obtained by X-ray Absorption Near-edge Structure (XANES) spectroscopy. The XANES spectra of Fe and Ni in LSTFN2, along with the reference spectra of SrFeO_3_, LaFeO_3_, LaNiO_3_, and NiFe_2_O_4_, are displayed in Fig. 1d, e. The Ni K-edge spectrum shows a good match with the one for NiFe_2_O_4,_ indicating that Ni species are also allocated in the spinel phase, thus corroborating the XRD findings. Regarding the Fe K-edge spectrum, Fe species in LSTFN2 resemble a combination of LaFeO_3_ and NiFe_2_O_4_, as can be noticed by the pronounced white line at 7130 eV and the neighboring shoulders at 7135 eV and 7142 eV. In contrast to what was observed on LSTFN2 annealed at low temperatures (*T* = 600 °C), the presence of SrFeO_3_ domains in the material, and so of Fe(IV) species, seems unlikely, due to the marked mismatch with the corresponding reference spectrum. This finding indicates that upon high-temperature treatment (*T* = 900 °C) SrFeO_3_ domains evolve into a more stable configuration.

Better insight into the Fe speciation was provided by Mössbauer spectroscopy. According to Fig. 1f, the Mössbauer spectrum of the pristine material consists of an intense paramagnetic absorption, centered around ≈0.36 mm/s, together with a broad sextet, attributable to the presence of magnetically coupled species. From the fitting of the spectrum, the two doublets are ascribed to bulk (Δ = 0.44 mm/s) and surface (Δ = 0.82 mm/s) ferric sites of the perovskite LSTFN2 lattice. The hyperfine parameters of the two other sextets can be attributed to the octahedral (*δ*: 0.38 mm/s and *H*: 50.6 T) and tetrahedral (*δ*: 0.28 mm/s and *H*: 47.1 T) sites of a partially inverse spinel lattice, as NiFe_2_O_4_ (Table S1). The broad linewidth of both sites (0.23 and 0.32 mm/s, respectively) suggests a certain disorder around the ferric sites, as a consequence of different local environments.

### Exsolution process

The parental oxide LSTFN2 underwent a reduction treatment to promote the exsolution of FeNi nanoparticles with negligible loss of SSA (Table 1). Treatments at different temperatures (*T* = 400–850 °C) were carried out with the intent to control the final composition of the exsolved phase.

**Fig. 2:**
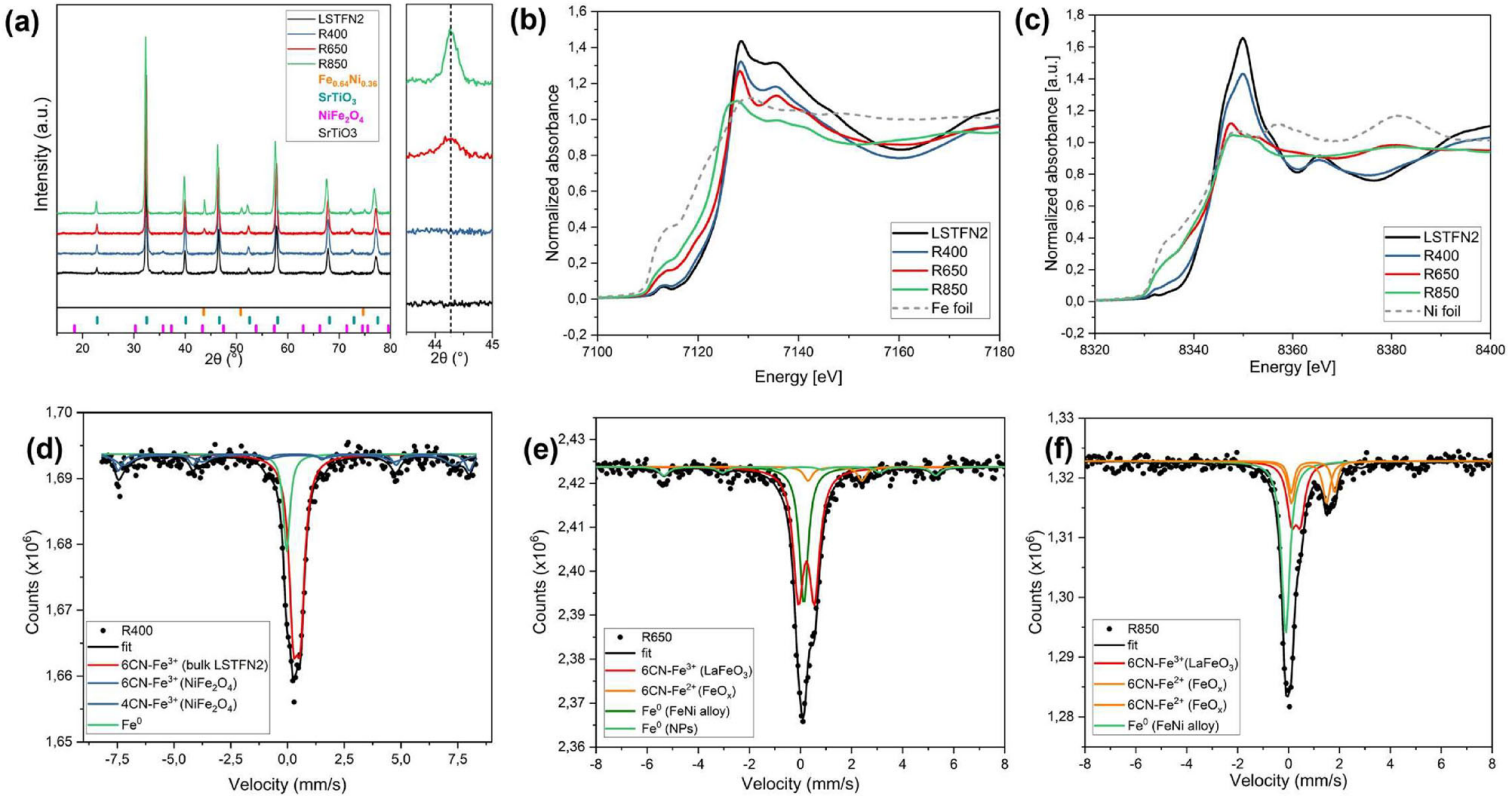
Structural changes upon the exsolution process. **a** XRD patterns of the as-synthesized, R400, R650, and R850 exsolved materials, together with a focus on the 2*θ* range of bimetallic alloys. The 2*θ* values of the reflections of Fe_0.64_Ni_0.36_, SrTiO_3_, and NiFe_2_O_4_ are also reported as references. **b** XANES absorption spectra for the Fe K-edge of the R400, R650, and R850 perovskite materials, together with the spectra used for qualitative comparison. **c** XANES spectra measured at the Ni K-edge for the as-synthesized, R400, R650, and R850 materials, together with reference spectra of Ni^0^ and NiFe_2_O_4_. **d**–**f** Mössbauer spectra of the R400, R650 and R850 materials, respectively.

From the XRD pattern of the material reduced at *T* = 400 °C, no metallic phases are visible. However, the darker color of the powder after reduction (Supplementary Fig. 2a) and, more significantly, the hydrogen consumption observed in the mass spectrogram acquired during the exsolution process (Supplementary Fig. 2b), proved that the reduction process took place even at such a low temperature. From Fig. 2a, it is evident how the perovskite matrix retained the cubic ${Pm}\bar{3}m$ structure even after reduction at 850 °C. The NiFe_2_O_4_ spinel phase disappears after the reduction treatment at 650 °C. Its vanishing is most likely linked to the appearance at this temperature of two metallic phases. The reflection at 2θ = 44.8° is attributed to an α-phase, either Fe or Fe–Ni, (lattice parameter *a* = 2.86 Å), whereas the more prominent reflection centered at 2θ = 43.77° (see magnification in Fig. 2a) is ascribed to a bimetallic Fe_3_Ni *γ*-phase (PDF-Ref. Nr.: 00-047-1405, space group ${Fm}\bar{3}m$). By increasing the exsolution temperature (*T* = 850 °C), the Bragg peak of the γ-phase increases in intensity, indicating a crystal growth up to 42 nm. The α-phase contribution in the R850 sample is almost absent, suggesting that at high temperature the alloy phase evolves to the ${Fm}\bar{3}m$ allotrope, which is the most thermodynamically stable phase. Interestingly, it can be noticed that the position of the (110) reflection of the perovskite parental structure shifts to lower 2*θ* values compared to the pristine material, thus indicating a lattice expansion, with the lattice parameter increasing from 3.910 Å to 3.923 Å (Supplementary Fig. 2c), indicative of oxygen vacancy formation during the exsolution process. Although the exsolved materials underwent significant structural modifications, their SSA remained comparable to the pristine sample (Table 1).

Further information regarding the reduction of the metallic species in the exsolved materials after the exsolution process at various temperatures is provided by XANES spectroscopy (Fig. 2b, c). Concerning the Fe spectra of R400, a slight increment of the pre-edge feature, as well as a decrease of the white line intensity are observed, indicating the beginning of the reduction process. For R650 and R850, a significant change in these features is visible. The absorption edge shifts towards lower energies, indicating the presence of Fe^2+^ and Fe^0^. Also, the shape of the white line, along with other spectral features, gradually resembles the shape of the metallic Fe^0^ spectrum. Interestingly, by increasing the reduction temperature up to 650 °C, the white line peak (at *E* = 7128.6 eV) gets sharper. Using the reference spectra as a comparison (see Supplementary Fig. 3a), this effect is likely ascribed to the consumption of the NiFe_2_O_4_ side phase, in agreement with the XRD data. Beyond 650 °C, the perovskite component LaFeO_3_ also gets massively converted both to a FeO and Fe^0^ phase. In the case of the Ni XANES spectrum of R400, a decrease in the white line intensity and a corresponding increase in the pre-edge feature are visible, indicating the presence of metallic Ni in the material. The spectra of the samples reduced at 650 °C and 850 °C are very similar and resemble a metallic nickel structure. This indicates two things: the first is that a great extent of Ni in the parental material has been reduced to the metallic state, and the second is that a significant amount of the Ni ions were contained in the NiFe_2_O_4_ phase.

To get better insights into the state of iron species during the exsolution process, Mössbauer spectroscopy was performed (Table 1, Fig. 2d–f, and Supplementary Table 1). The spectrum of R400 is dominated by a broad, intense, and asymmetric absorption centered around 0.28 mm/s, along with a magnetically coupled component of smaller intensity. From the four components (one singlet, one doublet, and two sextets) of the fitting, the doublet can be ascribed to distorted ferric sites in the LSTFN2 lattice, the singlet to a 15% of metallic iron (or an iron-nickel alloy) in a superparamagnetic regime (Supplementary Table. 1), confirming that even at temperatures as low as 400 °C a significant reduction of iron to the metal state takes place. The two sextets are compatible with octahedral (6CN) and tetrahedral (4CN) iron sites in the partially inverted spinel structure. This result perfectly agrees with the XRD and XANES measurements, as these ferric sites can be attributed to NiFe_2_O_4_, which is still present after the reduction at 400 °C and does not vary much from the pristine state. By increasing the reduction temperature to 650 °C, the metallic phase (identified by a singlet and a sextet) increased to 35% of the total amount of iron. In addition, a further contribution assigned to Fe^2+^ species in tetrahedral coordination is observed, thus indicating the presence of iron oxides by 5% formed during the course of the reduction process. At the last reduction stage at 850 °C, the amount of metallic iron or iron alloy increased to 43%, and that of Fe^2+^ species to 29%. The ferrous sites consist of two different species tentatively ascribed to Fe^2+^ in wustite (FeO) with different defective sites.

**Fig. 3:**
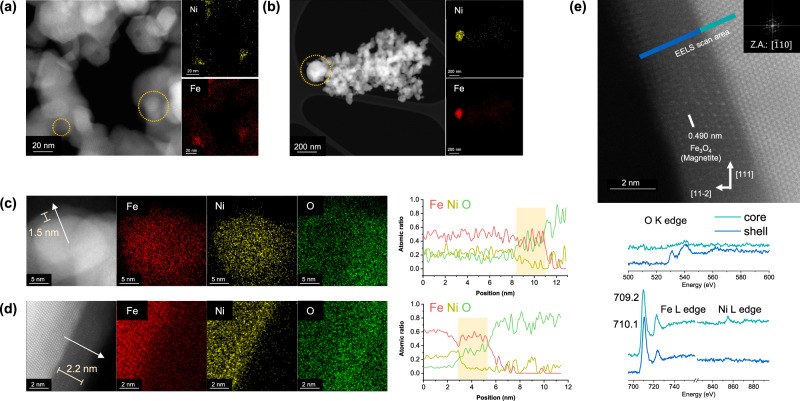
Microscopic analysis of the exsolved nanoparticles. HADDF-STEM image and respective iron, nickel, and oxygen EDX mapping result of the R400 (**a**) and R850 (**b**) materials; High-resolution images and EDX mapping result with line-scan signal (scan direction and shell thickness are denoted in the figure) of R400 (**c**) and R850 (**d**) materials; and **e** atomic resolution STEM and EELS result of R850, indicating the different composition of the core and the shell portions in the exsolved nanoparticles.

**Fig. 4:**
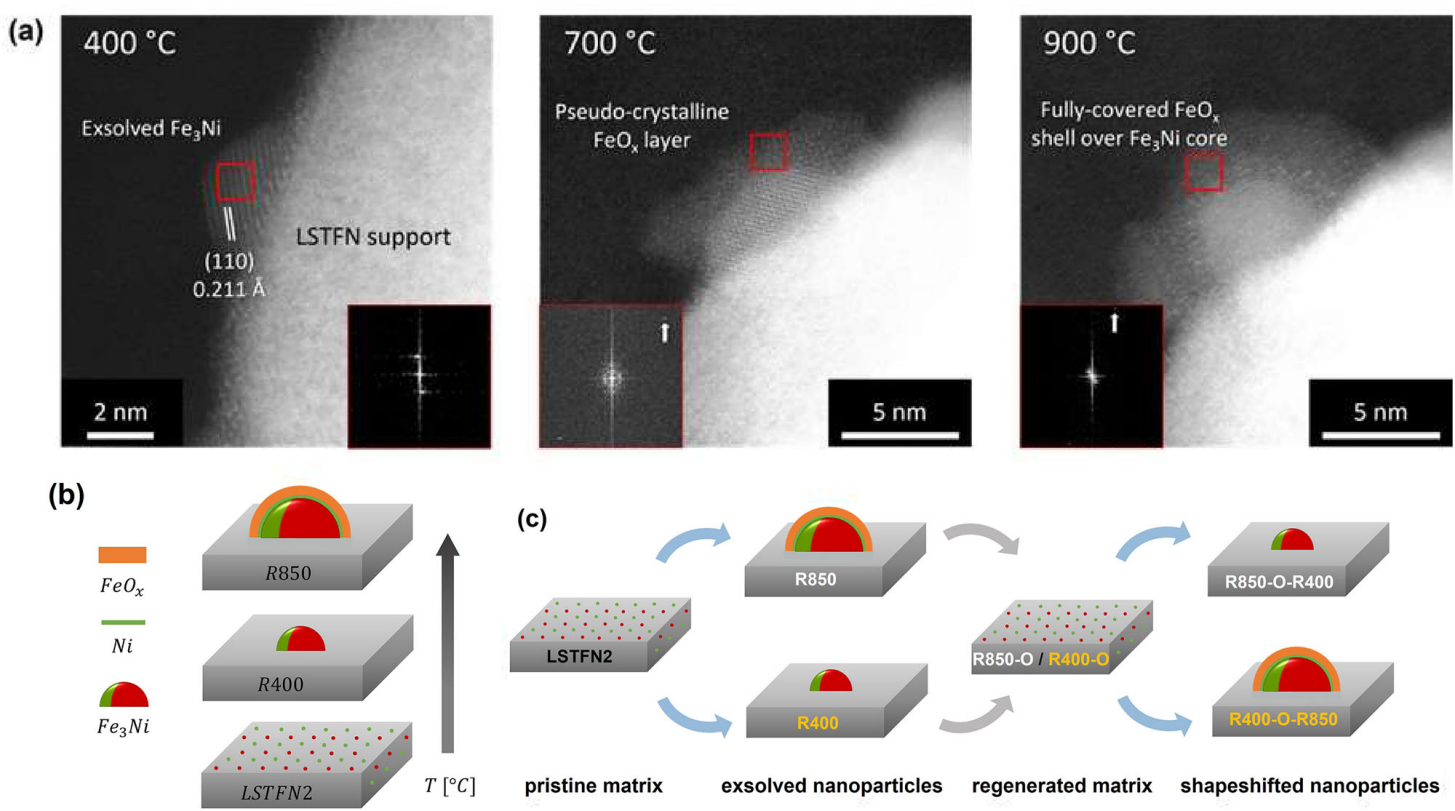
Evolution of exsolved NiFe alloy nanoparticles. Divergence of LSTFN2 support at an increasing temperature, followed by in situ TEM (**a**) and a schematic representation of it (**b**). Cartoon representing the shapeshifting mechanism applied to the exsolved NiFe nanoparticles mediated by the regeneration of the pristine perovskite matrix (**c**). Adapted with permission from ref. ^8^. Copyright 2022 American Chemical Society.

HR-TEM investigations of the exsolved materials show that NiFe segregations are present over the parent scaffold with a 20 nm diameter for R400 (Fig. 3a), confirming the Mössbauer analyses, the MS results, and the reduction-related color change. It should be noted that Fe is not readily reduced at low temperatures, however, we achieve Fe exsolution likely due to the hydrogen spillover effect and nucleation role mediated by Ni. As the reduction temperature increased up to 850 °C, the exsolved NiFe alloy segregation got larger, around 150 nm in diameter with higher crystallinity (Fig. 3b). The significant difference between the nanoparticle size compared to the XRD results from the fact that EDX mapping reveals the elemental segregation, whereas XRD reveals the average size of the crystalline domains of the alloy. To understand the microscopic structure of the exsolved NiFe species, high-resolution analysis was further conducted (Fig. 3c, d). As can be seen in the images, exsolved nanoparticles in R400 (Fig. 3d) have low crystallinity and are covered with a thin amorphous shell (~1 nm). EDXS-line scan analysis reveals that the outermost layer is iron oxide (FeO_*x*_), and the metallic core is composed of Fe_3_Ni. Such a core-shell structure could probably be formed by direct air exposure following the exsolution process, thus indicating the formation of an amorphous oxide passivation layer. Concerning the R850 system, the particle has a similar core-shell structure, but the outermost layer is crystalline (Fig. 3d). EDXS-line scan analysis indicates that the crystalline outermost layer (overlayer) is again iron oxide, and it is separated from the core Fe_3_Ni alloy by a thin Ni layer. This is a general trend observed also in the other particles. In Fig. 3e, an atomic resolution TEM image and electron energy loss spectroscopy (EELS) reveal that the overlayer comprises a spinel iron oxide structure (magnetite, space group: Fd-3m) presenting clear crystallographic periodicity. The spectra present a low tail height compared to the core, implying the oxidized feature. Those results are well-coincidental with the prior Mossbauer and XAS analysis, and likely to conclude that such a phase can readily form upon the exsolution process. The distinct difference of this iron oxide shell in R850 from that in R400, might be related to its different origin depending on the exsolution temperature. This difference would also confirm the findings of Mössbauer spectroscopy, which indicated that the formation of a highly defective iron oxide phase was increasingly formed by 650 °C. To get better insights into the formation of the exsolved core-shell structure, we performed in situ TEM experiments under high-vacuum conditions to avoid the disturbing effect of the air exposure (Fig. 4a). At *T* = 400 °C, NiFe metal nanoparticles were observed without the presence of an amorphous oxide layer. Interestingly, as the temperature increased, the pseudo-crystalline overlayer started to arise on the top of the NiFe alloy with clear contrast, and it eventually covered the core alloy at *T* = 900 °C. EDX results of the in situ TEM analyses show that the shell layer is composed of iron and oxygen only (Supplementary Fig. 4). Considering the high vacuum conditions of the measurement, it is more likely that the oxygen species are coming from the support material without the participation of gaseous species. It is not easy to provide a clear explanation for the formation of an iron oxide shell due to the high activity of the material at high temperatures. One possible reason could be due to the well-known strong metal support interaction (SMSI) of iron oxide. SMSI refers to a phenomenon in which the support material (typically a reducible oxide) strongly interacts with metal nanoparticles, often leading to the encapsulation of the metal surface by a thin oxide overlayer under reductive or high-temperature conditions^23,24^. In this system, iron exsolution occurs at low temperatures within the subsurface region (~10 nm) in which low-coordinated iron species bound by alpha oxygen are located (see Supplementary Fig. 5). As the temperature increases, SMSI is sequentially triggered on the exsolved particle, leading to the migration and reconstruction of bulk iron oxide species onto the particle surface. These migrating species, coordinated by more stable beta oxygen, form a passivating oxide layer over the metallic Fe_3_Ni core.

The selective formation of a FeO_*x*_ layer from the La_0.4_Sr_0.4_Ti_0.6_Fe_0.35_Ni_0.05_O_3-δ_ solid solution can be rationalized by considering the Tammann temperatures for the binary oxides forming the perovskite structure—i.e., the temperature at which ions in the bulk of a solid become thermally mobile and can diffuse more readily, approximately half of the material’s absolute melting temperature. Neglecting NiO due to its low content, FeO and Fe_3_O_4_ have Tammann temperatures of roughly 560 °C and 600 °C, respectively, whereas TiO₂, SrO, and La₂O_3_ exhibit much higher values, exceeding 800 °C. These estimates are in excellent agreement with our experimental observations. Based on extensive ex situ characterization—including XANES, Mössbauer spectroscopy, XRD, and TEM of reduced samples at different temperatures—supplemented by in situ TEM, it is observed that the formation of the iron oxide shell is temperature-activated, initiating at approximately 600 °C. In situ TEM and Mössbauer spectroscopy further reveal that the shell’s thickness and atomic ordering increase with temperature up to 850 °C. Mössbauer data indicate that its atomic composition resembles that of wüstite, whereas TEM imaging reveals magnetite domains. As both phases coexist in the Fe–O phase diagram^25^, it is reasonable to assume that the shell generally consists of a defective Fe_*x*_O_*y*_ layer with Fe(II/III) in a cubic crystal habit.

### Shapeshifting exsolution

So far, we have shown that exsolved nanoparticles with two different structures/compositions can be obtained by applying a low or a high reduction temperature. Now we want to demonstrate that it is possible to switch between these two nanoparticle conformations within the same material by mediating through an oxidative step at 850 °C, in which the pristine perovskite structure is regenerated and the metal nanoparticles are dissolved in the matrix (Fig. 4c). In this fashion, shapeshifting nanoparticles using the same parental oxide structure are obtained. The regeneration of the perovskite structure performed at *T* = 850 °C under oxidative conditions via the redissolution of the exsolved nanoparticles, represents a fundamental step for the shapeshifting exsolution process and requires, therefore, particular attention.

**Fig. 5:**
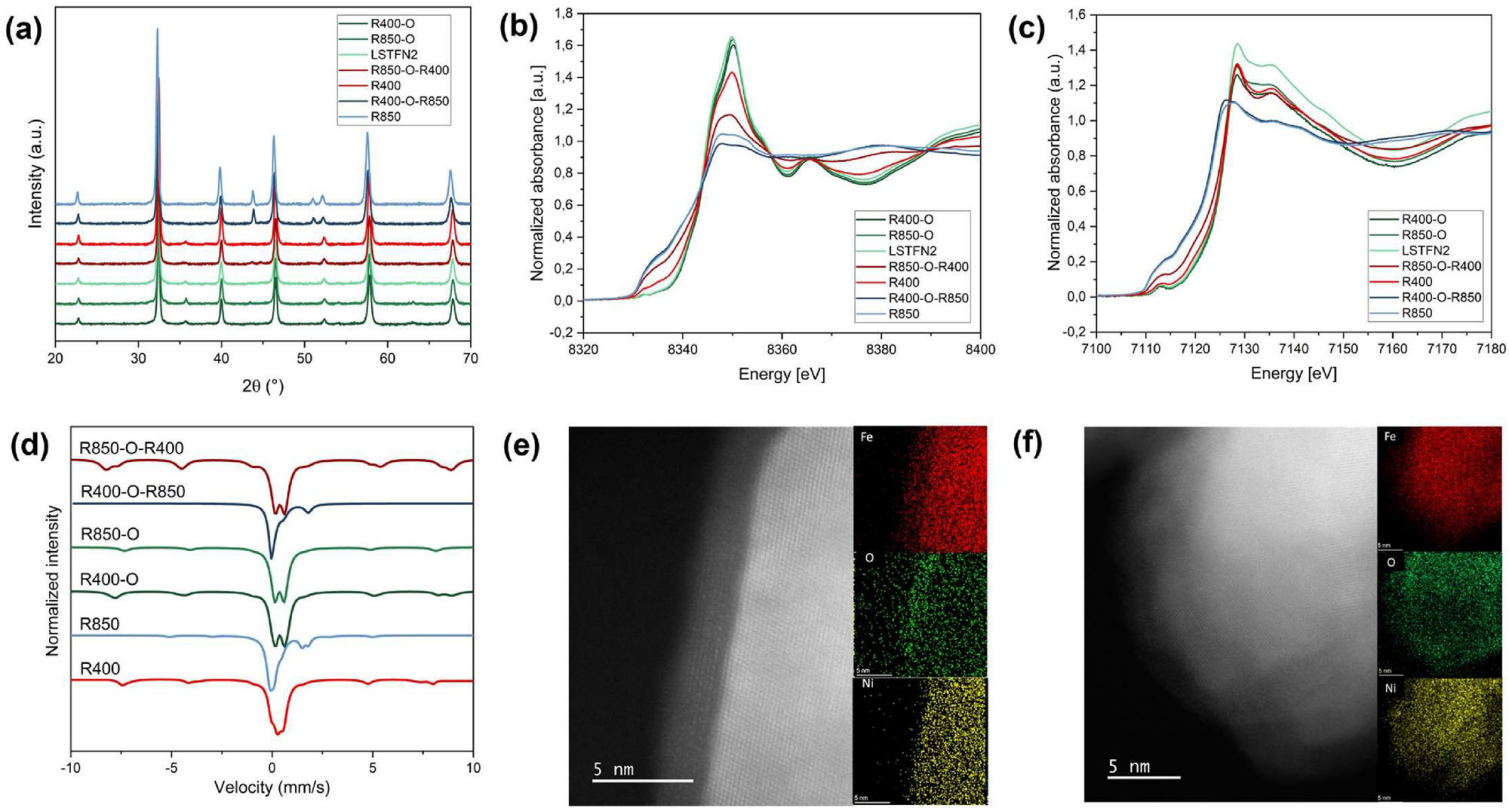
Effect of regeneration on the exsolved nanoparticles. **a** XRD patterns, **b** Ni K-edge XANES spectra, **c** Fe K-edge XANES spectra, and **d** Mössbauer spectra of the R400-O, R850-O, as-synthesized, R850-O-R400, R400, R400-O-R850, and R850 exsolved materials; HR-TEM pictures and EDX mapping of the **e** R400-O-R850 and **f** R850-O-R400 exsolved LSTFN materials.

In general, we can conclude that, because the oxidation temperature is equal to the reduction temperature for R850-O, a full reincorporation of the exsolved ions into the perovskite matrix can hardly be achieved. This can clearly be observed in the most intense XRD reflection of the spinel side phase and a residual signal of the bimetallic nanoparticles (Fig. 5a and Supplementary Fig. 6). The extent of the reincorporation in the parental oxide matrix is also dependent on the type of ion. From the complete superimposition of the Ni XANES spectra in Fig. 5b it is clear that Ni completely regains the chemical environment of the pristine LSTFN2 system. However, this is not the case for Fe, since it has a much larger concentration and thus it dissolves much more than nickel. Although the shape of the XANES curves at the Fe K-edge of the re-oxidized materials is the same as that of the pristine material (Fig. 5b), their intensity is significantly lower. This phenomenon can be ascribed both to the presence of metallic iron residuals, as shown in the XRD pattern of R850-O, and to a loss of symmetry in the geometric arrangement of the Fe cations as a consequence of the likely formation of disordered iron oxide species upon the reoxidation treatment. This latter hypothesis is confirmed during the Mössbauer analyses (Fig. 5d, Supplementary Fig. 7, Table 1, and Supplementary Table 1). Both R400-O and R850-O materials, in addition to the doublet signals belonging to the perovskite phase, present a sextet ascribed to the ferric site in a highly defective environment and assigned to the cation-deficient spinel maghemite, *γ*-Fe_2_O_3_, which would also explain the intensity increase of the spinel reflection in the XRD of R850-O. Hence, the reoxidation of the exsolved materials leads only to partial reincorporation of the exsolved ions into the parental oxide matrix. This is particularly true for the iron species that, due to their much lower mobility in contrast to nickel, form additional defective iron oxide phases.

The effectiveness of the shapeshifting exsolution process, i.e., the reduction of the reoxidized materials to the respective opposite original exsolution temperature (Fig. 5a), was first addressed by XRD. The pattern for R400-O-R850 is identical to the one for R850, with a pronounced signal of metallic exsolved particles ascribed to the Fe_3_Ni phase. However, when comparing R850-O-R400 with R400 after the shapeshifting process, the signal of the metallic FeNi nanoparticles is still clearly visible and is explained by the incomplete dissolution of the metallic phase in R850-O (Supplementary Fig. 6). However, this difference does not seem to affect the porosity as both shapeshifted materials possess the same SSA (Table 1). Looking at the re-reduction process of the single ions by XANES spectroscopy (Fig. 5b, c), it can be clearly seen that in both switched materials the white line and the pre-edge features of the Ni K-edge spectra strongly increased compared to the R400 and R850 systems. This indicates that the Ni species underwent a stronger reduction after the shapeshifting process. However, in the case of the Fe K-edge spectra, no significant difference is noticed before and after shifting. The higher metallic content (intensity increase of the pre-edge feature) observed in R850-O-R400 is a consequence of the incomplete dissolution of the metallic phase in R850-O, as shown by the XRD. This finding was further corroborated by Mössbauer spectroscopy (Fig. 5d, Supplementary Fig. 7, Table 1, and Supplementary Table 1). In the case of R850-O-R400 material, the re-exsolution at 400 °C seems to promote the formation of a consistent amount of magnetically ordered phases. The presence of a narrow absorption, centered at ≈0.4 mm/s, is typical of ferric sites in the LSTFN2 configuration. A broad magnetic pattern with two sextets indicates the formation of a consistent amount of magnetically ordered phases close to those obtained in the R400-O material, which are related to a partially inverse spinel. In addition to that, a small amount of metallic Fe phase is observed from the fitting. Concerning the material reduced at higher temperature, i.e., R400-O-R850, the spectrum components are exactly the same as those found in R850 (Table 1), indicating the presence of an iron metallic phase (40%) along with a highly defective iron oxide (25%). The optimal congruence between the materials exsolved and switched at high temperature, was further confirmed by TEM-EDX (Fig. 5e, f). Regarding the reduction at 850 °C, micrographs of the R400-O-R850 material indicate the formation of large alloy nanoparticles surrounded by a thick crystalline FeO_*x*_ shell (as in R850). Regarding the R850-O-R400 material, the nanoparticle microstructure and the elemental distribution were also found to be similar as in R400, indicating the presence of a Fe_3_Ni core surrounded by a thin oxide shell (Supplementary Fig. 8). However, the particle size in R850-O-R400 is far larger than in R400, probably as a consequence of metallic residuals from R850-O, which might act as sites for further particle growth during the re-exsolution process.

Clear evidence of the material transformation on a single system was provided by in situ XRD experiments. We performed two sets of measurements reproducing the R850-O-R400 and the R400-O-R850 redox cycles on LSTFN2 (Supplementary Figs. 9 and 10). Experimental details are reported in the supporting information. The XRD data reveal significant structural changes during reduction at 850 °C under H_2_. The emergence of a new peak at *Q* = 3.0 Å^−1^, coupled with a pronounced intensity increase at *Q* = 3.5 and 4.9 Å^−1^, is attributed to the formation of the Fe_0.64_Ni_0.36_ alloy nanoparticles. The concurrent decay of the peak at approx. 2.5 Å^−1^ confirms the depletion of the NiFe_2_O_4_ phase. Notably, exsolution was triggered consistently upon exposure to 850 °C in H_2_, independent of the sample’s thermal and redox history. The reversibility of this process was confirmed by the redissolution of the nanoparticles upon removal of either the reducing atmosphere or the high temperature. As shown in Fig. 5a, the particle regeneration (shapeshifting) is almost complete for R400-O-R850 and only partial for R850-O-R400. As a consequence of the prolonged high temperature oxidation followed by the re-reduction process, a slight decrease in the surface area is observed for the shapeshifted materials (Table 1).

### Catalytic experiments on exsolved materials

**Fig. 6:**
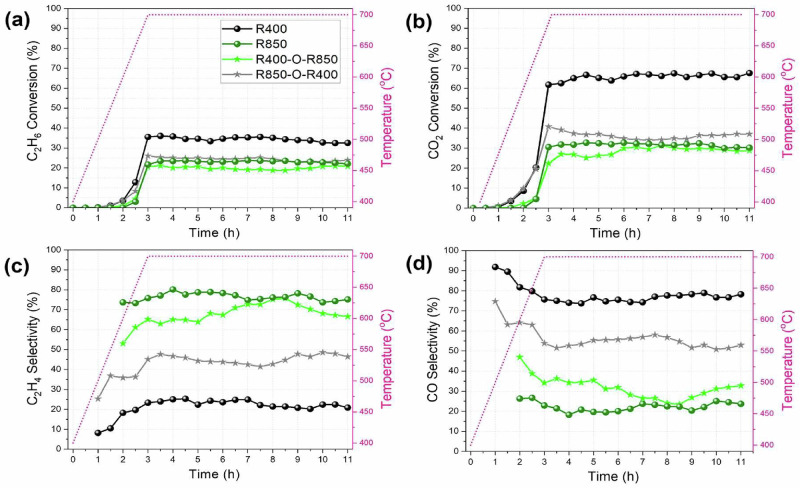
Catalytic activity of exsolution catalyst. Trend over time of the **a** C_2_H_6_ conversion, **b** CO_2_ conversion, **c** C_2_H_4_ selectivity (dehydrogenation), and **d** CO selectivity (reforming) during the catalytic testing consisting of 3 h of temperature ramping (400–700 °C) and 8 h of time-on-stream (700 °C constant).

The exsolved perovskites at 400 and 850 °C (R400, R850) were subsequently evaluated regarding their catalytic activity and selectivity for the reaction between C_2_H_6_ and CO_2_. The reactant conversions for C_2_H_6_ and CO_2_, as well as the product selectivities for C_2_H_4_ (dehydrogenation) and CO (reforming) are presented in Fig. 6. Supplementary Table 2 presents the overall reaction metrics at the end of the experiments. It is noted, that a small amount of CH_4_ is also produced via ethane cracking^2,26,27^, but its yield and selectivity values are quite small (below 1.5 %). During the first phase of temperature ramping from 400 up to 700 °C (first 3 h), the reactant conversions (C_2_H_6_ and CO_2_) increase steadily and then stabilize upon reaching the final temperature of 700 °C. During the second phase of time-on-stream (i.e., between the 3^rd^ and 11^th^ h), the rather limited change in the reactant conversion and product selectivity values is a testament to the great catalytic stability of the exsolved perovskite materials^8,28–30^.

The differences in the reactant conversion values between R400 and R850 are evident in Fig. 6a, b. In general, R400 can reach much higher reactant conversions compared to R850, although for CO_2_ conversion in particular, the difference is much greater. Most importantly, however, great differences are observed regarding the selectivity towards C_2_H_4_ and CO (Fig. 6c), or regarding the reaction pathways of ethane oxidative dehydrogenation (ODH) and dry ethane reforming (DER). For R400, the reforming pathway dominates, as the CO selectivity is stabilized at approx. 78% at 700 °C (Supplementary Table 2). In contrast, for R850, the dehydrogenation pathway is the dominant one, with the C_2_H_4_ selectivity stabilizing at approx. 75% at 700 °C. Therefore, it is shown that the different exsolution treatments over the same perovskite oxide material (LSTFN2) can effectively tune the reaction selectivity towards either the dehydrogenation or the reforming pathway.

**Fig. 7:**
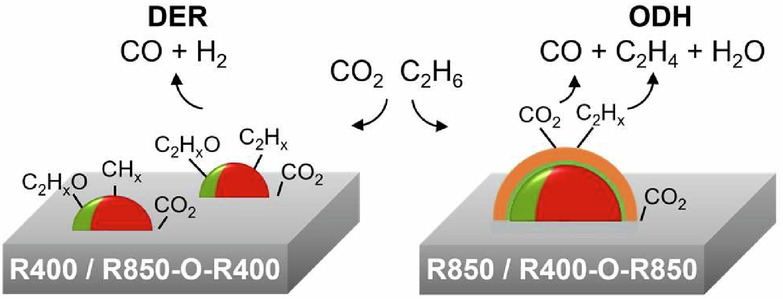
Schematic of the catalytic reaction. CO_2_-mediated ethane conversion mechanism over the exsolved and shapeshifted catalysts. Adapted with permission from ref. ^8^. Copyright 2022 American Chemical Society.

The reason for this great discrepancy in product selectivity during the reaction between C_2_H_6_ and CO_2_ can be traced back to the physicochemical properties of these two materials, such as the oxidation state of the Fe and Ni active metals, the material nanostructure, and the exposed catalytically active surface sites. For R400, the exsolved metal nanoparticles were shown to have a composition of Fe_3_Ni (Fig. 3c, d). The thin amorphous FeO_*x*_ shell observed during HRTEM (Fig. 3e), attributed to air exposure, is most likely easily removed under the reducing reaction conditions^9^. As a result, it is expected that the surface-exposed Fe_3_Ni metallic sites anchored on the redox-active and defect-rich LSTFN2 perovskite support render this material more active towards C–C bond scission and the ethane reforming pathway^2,8,31,32^. A possible reaction mechanism (Fig. 7) is that C_2_H_6(g)_ molecules are activated on the Fe_3_Ni metallic sites to various adsorbed intermediate species, including C_2_H_*x*(ads)_, C_2_H_*x*_O_(ads)_, and CH_*x*(ads)_ (the latter following C–C bond scission), while CO_2_ is activated on the support or the metal-support interface (e.g., on the surface oxygen vacancies) to generate CO_(g)_ and adsorbed oxygen species^8,27,32^. The strong electrophilic oxygen species supplied by the redox-active support can then further oxidize the adsorbed carbonaceous intermediates over the metallic sites to CO, thus favoring the reforming selectivity, all the while mitigating the amount of deposited coke and promoting the catalytic stability^8,17,19,31^.

On the other hand, for R850, the perovskite material is decorated with larger metallic Fe_3_Ni nanoparticles that are encapsulated by a thick crystalline FeO_*x*_ overlayer. This type of metal oxide/metal (M′O_*x*_/M) inverse interface has been previously reported to favor C-H bond scission and the ethane dehydrogenation pathway (ODH) over the reforming one (DER)^14,16,18,19,33^. In particular, Guo et al^19^. and Yan et al^14,18^. prepared various Fe–Ni bimetallic catalysts over different types of supports and concluded that the presence of a high amount of FeO_*x*_ (as overlayers, or in the interfacial sites) can prevent the scission of C–C bonds, while selectively splitting the C–H bonds, which is attributed to the supply of weakly electrophilic oxygen species by FeO_*x*_^19^. In particular, weakly electrophilic oxygen species can arise in some metal oxides (incl. FeO_*x*_ or Fe_3_O_4_), serving as Lewis acid sites and Lewis acid-base pairs, favoring ethane dehydrogenation to ethylene^34^. Furthermore, the presence of an FeO_*x*_ overlayer on top of a metallic core has been identified as more active compared to bulk FeO_*x*_ or just FeO_*x*_ adjacent to the support^14,16^. In our case, the thick, crystalline FeO_*x*_ overlayer over the metallic Fe_3_Ni particles, which is likely formed by SMSI during exsolution, is much harder to reduce under the catalytic reaction conditions. Also, it is expected to supply these weakly electrophilic oxygen species required for the selective C-H activation (initially to C_2_H_5(ads)_ and then to C_2_H_4(ads)_ and C_2_H_4(g)_), all the while blocking the access of the gaseous reactants to the reduced metallic sites (metal core) that are selective for C–C bond scission^14,16,19^ (as also indicated by CO chemisorption). The CO_2_ activation could take place either on the surface of the redox-active LSTFN2 support, the metal-support interface, or also on the FeO_*x*_ overlayer, and replenish the consumed active oxygen species^15,18,19,22^ (Fig. 7).

Additional CO chemisorption experiments were conducted to evaluate the accessibility of the Fe_3_Ni metallic core sites to gaseous species. As depicted in Supplementary Table 2, R400 can adsorb much more CO than R850. It should be noted, that R850 may hold more exsolved nanoparticles on the surface; however, it is less likely to interact with CO molecules, which suggests that the thick crystalline iron oxide overlayer in R850 covers the Fe_3_Ni metal core and thus stirs the reaction towards the dehydrogenation pathway.

In order to establish a comparison of the catalytic performance of R400 and R850 with other relevant materials reported in the literature, we prepared a comparison table, which can be found in the Supporting Information (Supplementary Table 3). In short, R850 compares quite favorably for the dehydrogenation reaction (ODH) even when compared to works using similar reaction temperatures (650–700 °C). R400 does provide a decent dry reforming reactivity, but it can be outcompeted by other reported catalysts operating at a similarly high temperature, provided that they also show high stability with minimal coke deposition. In any case, however, the ability to switch the catalytic selectivity over the same multifunctional material by simply altering the exsolution treatment temperature is a unique feature of the current work.

The materials following re-oxidation and shapeshifting exsolution, i.e., R850-O-R400 and R400-O-R850, were also tested (Fig. 6 and Supplementary Table 2). To some extent, the catalytic performance is different from that of the R400 and R850 materials. Such a result is somehow expected, due to the different surface characteristics of the host matrix and the re-exsolved nanoparticles, i.e., their incomplete dissolution during reoxidation, thus leading to the generation of different surface-active sites following the shapeshifting process. However, it can be observed that R400-O-R850 has a rather more similar catalytic performance to R850, with only slightly lower reactant conversion and C_2_H_4_ selectivity values, since a similar thick FeO_*x*_ overlayer and the inverse M′O_*x*_/M interface are also present in this material (Fig. 5e)^14,16,19^. In contrast, R850-O-R400 presents significantly lower reactant conversions than R400 and lower selectivity values towards ethane reforming. This can be ascribed to the significant surface reconstruction of the material during the prior re-oxidation treatment at 850 °C (R850-O), due to the presence of residual spinel oxide and bimetallic FeNi side phases, as previously confirmed by XRD, XANES, and Mössbauer spectroscopy (Fig. 5). As already mentioned, during re-reduction for R850-O-R400, these residual phases can act as sites for further particle growth, leading to significantly larger Fe_3_Ni metal nanoparticles when compared to R400, thus restricting the population of reforming active sites (for C–C bond scission). As a result, the shapeshifting treatment (re-oxidation and re-reduction) of the LSTFN2 material can be considered viable for switching the catalytic performance from DER to ODH, but it needs to be further optimized when the ODH to DER switch is required, due to the incomplete perovskite matrix regeneration during reoxidation in the latter case. As such, the switchability of catalytic performance upon shapeshifting largely depends on the ability of the reoxidation treatment to regenerate the parental oxide structure.

**Fig. 8:**
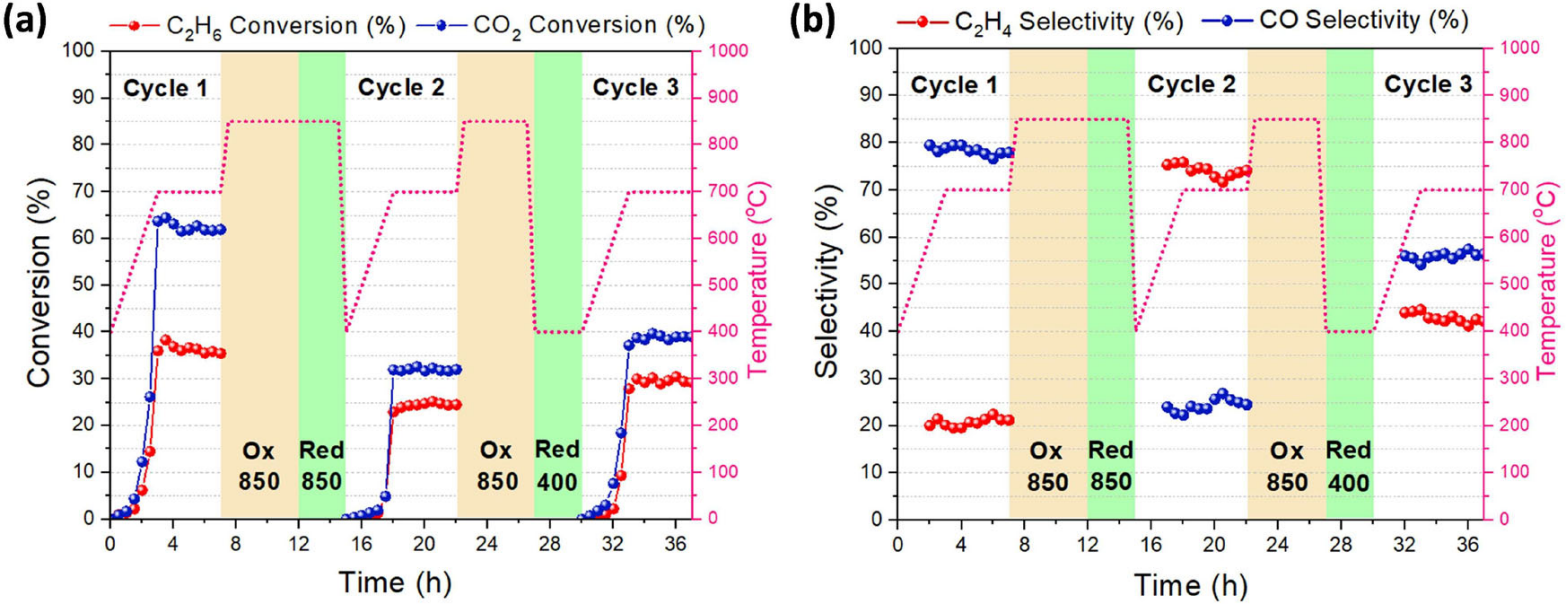
Effect of regeneration treatment on the catalytic activity. Trend over time for the **a** C_2_H_6_ and CO_2_ conversions, as well as **b** C_2_H_4_ and CO selectivities during the catalytic testing for three cycles (temperature ramping from 400 to 700 °C and time-on-stream at 700 °C constant) with intermediate in situ oxidation and reduction steps at either 850 or 400 °C.

To provide a comparison with a commercial reference catalyst, monometallic Ni/Al_2_O_3_ with 1 wt% Ni loading was also tested (Supplementary Fig. 11). It is shown that this commercial material is more active towards the DER pathway (as is expected for Ni-based catalysts)^2,27^. Despite the prior pretreatment step at 850 °C under pure H_2_ flow, it still displayed a continuous increase for the C_2_H_6_ and CO_2_ conversion values long after reaching the final reaction temperature of 700 °C. This hints at a continuous and slow material activation over time under the reactant stream and demonstrates the rather unstable nature of the commercial reference catalyst when compared to our perovskite-based multifunctional material.

The reactant conversion and product formation rates were also calculated at the end of the stability experiments and presented in Supplementary Table 4. Regarding ethane dehydrogenation, a comparison with the literature can be made by utilizing the work of Qiang et al.^35^, where it can be concluded that R850, with a STY for C_2_H_4_ at 692 μmol g_cat_^–1^ min^–1^ has a quite high C_2_H_4_ production rate compared to the other literature works, that is only slightly smaller to the higher value obtained in their own work at 700 °C (798 μmol g_cat_^–1^ min^–1^).

Additional experiments utilizing a 10 °C temperature step during the temperature ramping (560–660 °C) were carried out for the calculation of the activation energy values, and in order to compare catalyst behavior at similar (C_2_H_6_) conversion levels. The Arrhenius plots, along with small insets for the reactant conversion/product formation rates, can be found in Supplementary Fig. 12, whereas the activation energy values for C_2_H_6_ and CO_2_ conversion, as well as for CO and C_2_H_4_ production, are listed in Supplementary Table 5. These activation energy values are in agreement with those reported in other literature works^8,19,21,32,36–39^. In all of the materials, the CO_2_ activation energy values are lower than those for C_2_H_6_ activation, which can be ascribed to the concurrent side-reaction of reverse water-gas shift (RWGS)^32,38^. The activation energy for CO production generally follows the trend of CO_2_ and C_2_H_6_ conversion, whereas for C_2_H_4_ production, the values are quite higher, even for the ODH-selective materials. By comparing the different materials, R400 has the lowest activation energy values, due to its significantly pronounced low-temperature catalytic activity. On the contrary, the activation energy values are the highest for R850, whereas the regenerated samples (R850-O-R400 and R400-O-R850) show intermediate values.

A comparison is also made between the different materials at similar C_2_H_6_ conversion levels (7–8% C_2_H_6_ conversion values, temperature range 630–660 °C, Supplementary Fig. 13). As shown in Supplementary Fig. 13, the CO_2_ conversion varies greatly between the materials, as do the ethane-basis selectivity values for CO (DER) and C_2_H_4_ (ODH). In particular, R400 shows the highest DER selectivity and R850 the highest ODH selectivity, with the regenerated samples falling in between (particularly R850-O-R400). This verifies that the observed activity and selectivity differences stem from the intrinsic catalytic activity (kinetics)^14^.

Subsequently, in order to evaluate whether the aforementioned catalytic performance/selectivity switch (and thus the corresponding material structural transformations) can occur in situ, a new cyclic catalytic experiment was designed, which consisted of three catalytic cycles with intermediate in situ oxidation and reduction treatment steps. In each catalytic cycle, the same procedure of temperature ramping (400–700 °C) and time-on-stream (700 °C constant) was performed as in Fig. 6 (with just a reduced time-on-stream duration of 4 h). In situ oxidation was always performed at 850 °C, whereas in situ reduction was performed alternately at 850 and 400 °C in order to mimic the corresponding ex-situ treatments of the regenerated materials that were previously tested. The results are presented in Fig. 8.

From Fig. 8, it is clear that the first catalytic cycle (Cycle 1) for the R400 material proceeds similarly to the experiment in Fig. 6 (relatively high C_2_H_6_ and CO_2_ conversions that remain relatively stable at 700 °C). The R400 material is quite selective towards DER, with approx. 78% CO selectivity. Following in situ oxidation and reduction at 850 °C, a dramatic shift in the catalytic performance during Cycle 2 is observed. On the one hand, lower C_2_H_6_ and CO_2_ conversion values are reached at 700 °C, but on the other hand, the reaction selectivity is completely switched in favor of oxidative ethane dehydrogenation (ODH), with C_2_H_4_ selectivity of approx. 74%. This catalytic behavior is quite similar to R400-O-R850 with the corresponding ex-situ oxidation/reduction treatments, but without undergoing prior catalytic operation, and even to the freshly exsolved R850 material (Fig. 8).

Afterwards, during Cycle 3, and following in situ oxidation at 850 °C and in situ reduction at 400 °C, an attempt is made to revert the catalytic activity and selectivity back to the initial one of R400 (Cycle 1). However, the C_2_H_6_ and CO_2_ conversion values are stabilized at lower levels than during Cycle 1 (though higher than during Cycle 2), and the selectivity is rather intermediate between DER and ODH (though slightly favoring DER), at approx. 56% CO selectivity vs 42% C_2_H_4_ selectivity (a small amount of CH_4_ is also produced in all cycles). This means that the catalytic performance and selectivity switch from ODH to DER is only partial. This is also reflected, however, in the previous catalytic performance results (Fig. 6) for R850-O-R400, since, as explained before, the initial active state of R400 cannot be completely regenerated. Therefore, although an efficient selectivity switch from DER to ODH following in situ regeneration over the same material was achieved, future research work needs to be directed toward the complete structural regeneration of the initial perovskite structure, in order to achieve complete catalytic performance and selectivity switch during multi-cyclic operation.

**Fig. 9:**
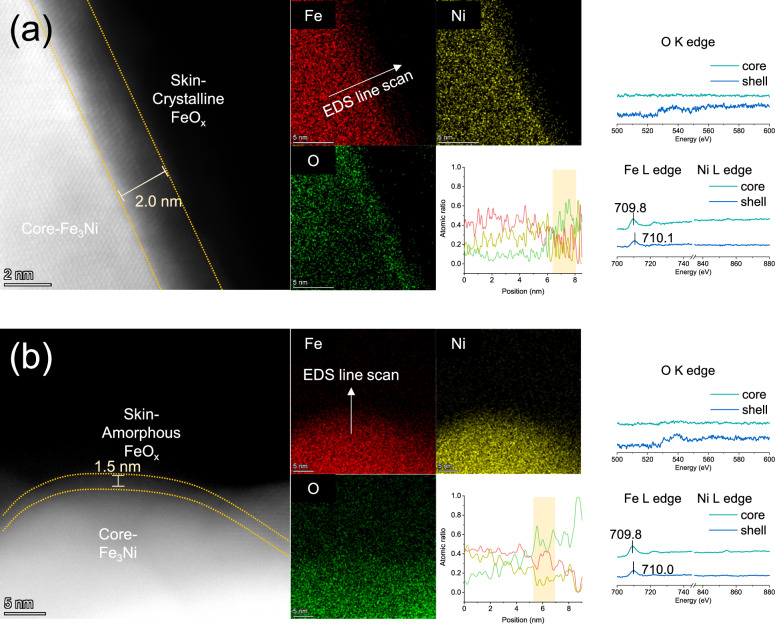
Post characterization of the spent catalyst. Atomic resolution HADDF-STEM images and corresponding EDX mapping and EELS results on **a** spent R850, and **b** spent R400.

Spent material characterization after the reaction (post-catalysis) was also carried out. It is shown that the spent perovskites display no signs of coke deposition, in contrast to spent Ni/Al_2_O_3_, where coke deposition is evident via the appearance of intense D and G bands in the Raman spectra (Supplementary Fig. 14), attributed to turbostratic carbon^40–44^. TEM images of the spent perovskites also show the lack of carbon growth over these materials (Supplementary Figs. 15–18), whereas for Ni/Al_2_O_3_, the formation of typical carbon baffles is observed (Supplementary Fig. 19). XRD was also performed for the spent catalysts (Supplementary Fig. 20), although in this case, the presence of high amounts of quartz sand in the catalyst bed gave rise to intense quartz (SiO_2_) diffractions. The perovskite reflections can, however, be observed, alongside the presence of crystalline Fe_3_Ni alloy phase (2theta = 43.7°) in the exsolved and regenerated perovskite materials. In addition, we conducted HAADF-STEM, EDX, and EELS analysis on the post-catalysis (spent) samples R400, R850, R850-O-R400, and R400-O-R850 to study potential material nanostructure changes of the spent catalysts following the catalytic testing procedure. Figure 9a represents the exsolved nanoparticle for spent R850 after the catalytic reaction, which clearly shows the intact crystalline FeO_*x*_ overlayer (also present in the fresh/exsolved sample) even after the prolonged reaction operation. The enrichment of iron species was observed in the line scan area (~2 nm), with less abundant Ni. The EELS result further reveals that the outermost layer is composed of iron oxide (710.1 eV), with distinct differences in core metals. In the case of the spent R400, the outermost FeO*x* layer presents an amorphous-like state (Fig. 9b), which is attributed to ex-situ air exposure during the handling process (similarly to fresh/exsolved R400). In the O K edge, it reveals broader peak intensity compared to the R850, indicative of less hybridization between Fe and O, presumably resulting from the native oxide^45,46^. Thus, it is likely to note that metallic FeNi nanoparticles are the major active species for R400, while FeNi nanoparticles encapsulated by FeO_*x*_ overlayer are the major active species for R850, and that the reaction conditions do not alter these initial active species any further. We further investigate the crystallinity of the outermost layer for the redox-treated (regenerated) spent samples (R400-O-R850 and R850-O-R400). As can be seen in the TEM images of Supplementary Fig. 21, the active species vary depending on the final reduction treatment. For R400-O-R850, the outermost layer presents crystallinity with a relatively well-resolved O K edge spectrum in EELS, while the R850-O-R400 shows an amorphous FeO_*x*_ layer similar to what we’ve observed in reduced samples, and these are well coincident with our prior in situ XRD results. We would like to note that they are maintained during catalysis when compared to the corresponding fresh/exsolved samples, as in the cases of R400 and R850, owing to the high structural stability of all the perovskite-derived materials used in this study.

In the present work, we showed how the shape and composition of FeNi bimetallic nanoparticles can be tailored by the exsolution approach as a function of temperature. Reduction treatments at temperatures as low as 400 °C formed small bimetallic Fe_3_Ni nanoparticles, whereas treatments at higher temperatures (*T* = 850 °C) produced a core-shell system of FeO_*x*_@Fe_3_Ni. The iron oxide shell formation was explained to originate via SMSI combined with reverse oxygen spillover from the perovskite oxide matrix. We demonstrated that we can shapeshift these nanoparticle conformations within the same host to a great extent by regenerating the perovskite oxide under oxidative conditions and re-reducing it at the opposite temperature.

The application of such exsolved nanostructures in the CO_2_-mediated ethane conversion indicated that the Fe_3_Ni and FeO_*x*_@Fe_3_Ni nanostructures, obtained via different exsolution treatments (at 400 °C and 850 °C, respectively) over the same multifunctional perovskite parent material, were highly selective for the ethane dry reforming and the ethane oxidative dehydrogenation pathway, respectively. We additionally showed that the regeneration/shapeshifting process results in a switching of the catalytic performance/selectivity (also in situ) to a great extent.

In this way, we demonstrate that the very same material can be used both for ethane dry reforming and ethane oxidative dehydrogenation using CO_2_, by simply changing the exsolution (reduction) conditions, and that even switching of catalytic performance/selectivity can be achieved following in situ oxidation/reduction, at least partially. However, to reach full switching of the catalytic performance during multi-cyclic operation, further work needs to be done to improve the oxidative nanoparticle dissolution and the complete regeneration of the initial perovskite host. In the era of the energy transition, these results pave the way towards the development of innovative multifunctional sustainable catalysts, which adapt to complex catalytic processes.

## Method

### Chemicals

Titanium (IV) isopropoxide (97%, Alfa Aesar), strontium nitrate (99%, Acros Organics), iron (III) nitrate nonahydrate (98%, Alfa Aesar), lanthanum (III) nitrate hexahydrate (99.9%, Alfa Aesar), nickel (II) nitrate hexahydrate (97%, Sigma-Aldrich) anhydrous citric acid (99.6%, Acros Organics), glycerol (99%, Alfa Aesar) were used as received without further purification.

### Material synthesis

For the purpose of this study, the system of interest is a A-site deficient, B-site-doped strontium titanate-based perovskite oxide, with stoichiometric formula La_0.4_Sr_0.40_Ti_0.6_Fe_0.35_Ni_0.05_O_3±δ_ (hereafter abbreviated as LSTFN2), which has been designed with 20% A-site deficiency for promoting the migration of B-site Fe and Ni metal ions and therefore favoring the exsolution process. The mesoporous perovskite powders have been synthesized via a template-free modified Pechini method^36^. In such a synthesis procedure, glycerol has been selected as polyol; this choice is related to its better chelating and crosslinking properties, when compared with conventionally used ethylene glycol^37,38^. In this synthesis, titanium (IV) isopropoxide (Ti(OCH(CH_3_)_2_)_4_) was initially combined with 11.9 ml of glycerol and stirred at room temperature for 30 min. Then, 40.7 mmol of citric acid were added, and the solution was heated to 60 °C using an oil bath. After 45 min of stirring to dissolve the citric acid, stoichiometric amounts of Sr(NO_3_)_2_, La(NO_3_)_3_∙6H_2_O, Fe(NO_3_)_3_∙9H_2_O, and Ni(NO_3_)_2_∙6H_2_O were sequentially added at 30 min intervals. Before adding Sr(NO_3_)_2_, the salt was dissolved in the lowest amount of deionized H_2_O possible and stirred for 30 min. The molar ratio of glycerol to citric acid to the total metal cations was set at 30:4:1. Once all the metal precursors were added, the solution was further stirred for 2 h to ensure thorough mixing. Subsequently, the temperature was raised to 130 °C, and the solution was maintained at this temperature for another 2 h under continuous stirring. The combination of polyol (glycerol) and polycarboxylic acid (citric acid) resulted in the formation of a polyester gel through a polycondensation reaction. The polymer gel was then subjected to calcination at 400 °C for 2 h, followed by heating at 900 °C for another 2 h, using a temperature increase rate of 2 °C min^−1^. The prepared as-synthesized perovskite is named LSTFN2, with “2” representing the 20% A-site deficiency of the material.

### Characterization methods

Powder X-ray diffraction was carried out for the phase analysis of the synthesized materials. This has been performed with an X’Pert Pro diffractometer from PANalytical Corp., using a 1.5406 Å Ni-filtered Cu-K_α_ radiation, operating at 45 kV and 40 mA. The average crystallite sizes of the different materials were calculated from the full width at half maximum of the (101) reflection by using the Scherrer equation. The X-ray absorption spectra at the Ni K edge (8333 eV) and Fe K-edge (7112 eV) were acquired at the P64 beamline at the Deutsches Elektronen Synchrotron (DESY, Hamburg, Germany). A Si(111) double crystal monochromator was employed for the energy scans around the respective metal absorption edge. The XAFS spectra were collected in fluorescence geometry for both the Ni and Fe K-edges using gas ionization chambers to measure the photon intensities before and after the samples. The powders were diluted in cellulose, pressed into pellets, and then covered in Kapton® tape before placing them in the beam path. Normalization of the obtained XANES data was carried out by performing pre-edge and post-edge background subtraction in the XANESA software. The same software has also been employed for the plotting and the comparison of the normalized data. The nitrogen physisorption isotherms were obtained at −196 °C using a Quadrasorb SI-MP b Quantachrome. Outgassing was performed with a Masterprep Degasser (Quantachrome Corp.) at 120 °C for 12 h. The SSA of pristine and exsolved materials was determined with the Brunauer–Emmett–Teller (BET) method^39^ at *P*/*P*_0_ = 0.07–0.3.

CO chemisorption was conducted through pulse mode with AutoChem II (Micrometrics). Prior to the experiment, 0.1 g of the sample was reduced for 3 h at either 400 or 850 °C under 10 % H_2_ in Ar. After the sample was cooled to 50 °C, a 10% CO in He pulse was injected, and the amount of consumed CO was calculated. The composition of the bimetallic nanoparticles was investigated with Corrected Scanning High-Resolution Transmission Electron Microscope (Cs-TEM) measurements at KARA (KAIST Analysis center for Research Advancement). These included photographs of the samples through HR-TEM and the EDX (energy-dispersive X-ray) microscopy. (FEI Tecnai Titan cube G2 60-300) The acceleration voltage was fixed at 300 keV. For the in situ experiment, powder samples are dispersed on the silicon nitride grid (Nanofactory, MEMS microheater), and further loaded in a heating holder (FEI Nano-Ex). During the experiment, the vacuum level was maintained under 9 × 10^−8^ mbar. Electron energy loss spectroscopy (EELS) was collected for the R850 sample with a dispersion of 0.25 eV per channel for the L edge of Fe (700–725 eV), Ni (850–880 eV), and the K edge for O (532 eV). Room temperature Mössbauer spectroscopy was performed on a conventional constant acceleration spectrometer mounting a Rh matrix ^57^Co source, nominal strength 1850 MBq. The hyperfine parameters were obtained by means of standard least-squares minimization techniques. The Mössbauer parameter *δ* is quoted relative to *α*-Fe foil. In situ synchrotron XRD was performed at the P08 beamline at PETRA III, DESY, with a photon energy of 25 keV and a beam size of 100 μm × 400 μm. The LSTFN2 powder was filled in a quartz capillary with a 2 mm diameter, open at both ends. To prevent sample movement during gas flow, the powder was confined by quartz wool plugs on either side. One end of the capillary was connected to a gas-mixing system that regulated the type and flow rate of the gas, while the outlet was connected to a mass spectrometer to verify the gas flow and monitor the reaction products. The detailed procedure of the in situ experiments is reported in the supporting information. The exsolution of the materials consisted of a treatment at high temperature of the catalyst powders under reducing conditions. A U-shaped quartz tube was first filled with 0.1 g of powder, and a heating ramp of 10 °C min^−1^ was set while flushing Ar in the system, allowing for an inert atmosphere during the heating section. The temperature of the system was measured through a Pt-100 thermocouple mounted within the exsolution cell, for real-time monitoring of the local temperature of the powder. Finally, once reaching the target temperature, the quartz tube was then subjected to a flow rate of 20 ml min^−1^ of 5% H_2_ in N_2_ for 3 h, providing the reducing conditions necessary for the nanoparticle formation. In a similar way, the re-oxidation treatments for the partial reincorporation of the Fe and Ni species in the parent perovskite structure were performed in the same experimental apparatus as the exsolution treatments. The quartz U-tube was filled with the exsolved (black) powder and subjected to a flow rate of 20 ml min^−1^ of air at 850 °C, which was flushed in the system for 5 h. The outlet composition of the exiting gas was monitored through a mass spectrometer. The extracted materials were named “RT”, with *T* indicating the temperature of the treatment. The materials have been first exsolved at three reference temperatures: one lower (400 °C, sample name “R400”), one intermediate (650 °C, sample name “R650”), and one higher temperature (850 °C, sample name “R850”). Each material was exsolved for 3 h by subjecting it to a flow of 20 ml min^−1^ of formation gas (5% H_2_ in N_2_). After the first exsolution treatment, the R400 and R850 materials were partially regenerated by re-oxidation at high temperature (850 °C) for 5 h under air. This has been carried out to induce the re-incorporation of the metallic species back into their lattice positions within the parent perovskite oxide. The samples thus obtained were referred to as “R850-O” and “R400-O”, for the materials initially exsolved at 850 °C and 400 °C and then re-oxidized, respectively. Finally, the regenerated samples have been once again reduced at the correspondent opposite temperature. That means, the “R400-O” was then re-exsolved (re-reduced) at 850 °C (sample name: “R400-O-R850”), and the “R850-O” was then re-exsolved at 400 °C (sample name: “R850-O-R400”). Raman spectra for the spent catalysts were recorded on a Bruker Senterra Raman microscope (*λ* = 532 nm, *P* = 20 mW). Measurements were acquired by performing 16 scans with an integration time of 16 s each, within the range 50–2740 cm^−1^_._

### Catalytic testing

Catalytic testing of the exsolved and regenerated perovskite materials (R400, R850, R850-O-R400, and R400-O-R850) was performed in a quartz glass tubular reactor (9 mm I.D.) at atmospheric pressure. Initially, 0.1 g of exsolved LSTFN2 perovskite oxide material was mixed with 0.5 g of quartz sand and loaded in the reactor. The total flow rate was set at 40 ml min^−1^ (10 ml min^−1^ C_2_H_6_, 10 ml min^−1^ CO_2_, and 20 ml min^−1^ Ar), with the C_2_H_6_:CO_2_ molar ratio being kept at 1:1. The initial reaction temperature was 400 °C, following a prior brief purge with Ar. The reaction procedure was carried out in two phases. During the first phase (activity testing), after passing the reaction mixture through the reactor at 400 °C, the reaction temperature was slowly increased with a rate of 100 °C per h (50 °C for every 30 min), up to 700 °C. During the second phase (time-on-stream), after the reactor temperature reached 700 °C (3 h), the temperature was kept constant at 700 °C for another 8 h. The testing of the 1% Ni/Al_2_O_3_ reference catalyst included a prior in situ reduction step under pure H_2_ flow at 850 °C for 1 h. A cyclic experiment (starting from R400) with intermediate in situ oxidation and reduction treatments was also carried out in the same reactor setup. The same catalyst weight, reactant flows, and temperature ramping procedure as above were used, but the time-on-stream duration was reduced to 4 h. Following each catalytic cycle, oxidation (Ox) was carried out in situ by increasing the temperature to 850 °C under Ar and then flowing 20% O_2_ in Ar for 5 h. In situ reduction was then carried out (following a brief Ar purge) by flowing 5% H_2_ in Ar for 3 h, either at 850 °C (before Cycle 2) or at 400 °C (before Cycle 3). Heating up and cooling down were always carried out under an Ar flow. In order to calculate the activation energy values and compare the catalytic performance at similar (C_2_H_6_) conversion levels, additional temperature step experiments were conducted utilizing a temperature step of 10 °C in the 560–660 °C range, keeping the same catalyst weight and reactant flows as before. The gaseous reaction products were analyzed online via an Agilent-7890A gas chromatograph, as described in our previous work, ref. ^8^. The ethane and carbon dioxide conversions, the hydrogen, carbon monoxide, ethylene, and methane yields, as well as the selectivities (ethane-basis) for ethylene (dehydrogenation), methane (cracking) and carbon monoxide (reforming), together with the H_2_/CO molar ratio were determined by using the following equations (Eqs. 3–12)^8^:

3 $${X}_{{{{C}}}_{2}{{{H}}}_{6}}=\,\frac{{F}_{{{{C}}}_{2}{{{H}}}_{6,{in}}}-{F}_{{{{C}}}_{2}{{{H}}}_{6,{out}}}}{{F}_{{{{C}}}_{2}{{{H}}}_{6,{in}}}}\times 100$$

4 $${X}_{{{CO}}_{2}}=\,\frac{{F}_{{{CO}}_{2,{in}}}-\,{F}_{{{CO}}_{2,{out}}}}{{F}_{{{CO}}_{2,{in}}}}\,\times 100$$

5 $${Y}_{{{{H}}}_{2}}=\,\frac{{F}_{{{{H}}}_{2,{out}}}}{3\times {F}_{{{{C}}}_{2}{{{H}}}_{6,{in}}}}\,\times 100$$

6 $${Y}_{{CO}}=\,\frac{{F}_{{CO},{out}}}{(2\times {F}_{{{{C}}}_{2}{{{H}}}_{6,{in}}}+\,{F}_{{{CO}}_{2,{in}}})}\times 100$$

7 $${Y}_{{{{C}}}_{2}{{{H}}}_{4}}=\,\frac{{F}_{{{{{C}}}_{2}{{H}}}_{4,{out}}}}{{F}_{{{{C}}}_{2}{{{H}}}_{6,{in}}}}\,\times 100$$

8 $${Y}_{{{CH}}_{4}}=\,\frac{{F}_{{{CH}}_{4,{out}}}}{2\times {F}_{{{{C}}}_{2}{{{H}}}_{6,{in}}}}\,\times 100$$

9 $${S}_{{{CH}}_{4}}=\,\frac{{Y}_{{{CH}}_{4}}}{{X}_{{{{C}}}_{2}{{{H}}}_{6}}}\,\times 100$$

10 $${S}_{{{{C}}}_{2}{{{H}}}_{4}}=\,\frac{{Y}_{{{{C}}}_{2}{{{H}}}_{4}}}{{X}_{{{{C}}}_{2}{{{H}}}_{6}}}\,\times 100$$

11 $${S}_{{CO}}=100-\,{S}_{{{CH}}_{4}}-\,{S}_{{{{{C}}}_{2}{{H}}}_{4}}$$

12 $${{{H}}}_{2}/{CO}=\,\frac{{F}_{{{{H}}}_{2,{out}}}}{{F}_{{CO},{out}}}\,$$

## Supplementary information

Supplementary Information

Transparent Peer Review file

## Data Availability

Data are available from the corresponding authors upon request.
