# Supplementary material for: Shapeshifting exsolved FeNi bimetallic nanostructures as catalytic switchers during the CO2-mediated ethane conversion
Source: Nat Commun. 2026 Apr 13;17:5117. doi: 10.1038/s41467-026-71282-6 (PMC13247154; doi:10.1038/s41467-026-71282-6)
Supplement: Supplementary file 1 — Supplementary Information [file 41467_2026_71282_MOESM1_ESM.pdf]

Supplementary Information

Shapeshifting exsolved FeNi bimetallic nanostructures as catalytic switchers during the CO_2-mediated ethane conversion

Filippo Colombo[1‡], Anastasios I. Tsiotsias[2‡], DongHwan Oh[3,4‡], Luca Nodari[5,6], Georgios I. Siakavelas[2], Linda Joseph[7], Xiao Sun[7,8] Nikolaos D. Charisou[2], WooChul Jung[9,10]* Maria Goula[2,11,12]*, and Simone Mascotto[7]*

[1] Institute of Inorganic and Applied Chemistry, University of Hamburg, Martin-Luther-King-Platz, 6, 20146 Hamburg, Germany

[2] Department of Chemical Engineering, University of Western Macedonia, ZEP, Kozani, 50100, Greece
[3]Department of Material Science and Engineering, Korean Advanced Institute of Science and Technology, Daehak-ro, Yuseong-gu, Daejeon 34141, Republic of Korea
[4]Department of Chemical and Biomolecular Engineering (BK21 Four), Korea Advanced Institute of Science and Technology (KAIST), Daejeon 34141, Republic of Korea
[5]Dipartimento di Scienze Chimiche, Università degli Studi di Padova, via Marzolo 1, 35131 Padova, Italy
[6]Institute of Condensed Matter Chemistry and Technologies for Energy, National Research Council. C.so Stati Uniti 4, 35127 Padova, Italy
[7]Institute of Integrated Natural Science, University of Koblenz, Universitätsstraße 1, 56070 Koblenz, Germany
[8]Deutsches Elektronen-Synchrotron, Notkestraße 85, 22607 Hamburg, Germany
[9]Department of Materials Science and Engineering, Seoul National University, 1, Gwanak-ro, Gwanak-gu, Seoul 08826, Republic of Korea
[10]Research Institute of Advanced Materials, Seoul National University, 1, Gwanak-ro, Gwanak-gu, Seoul 08826, Republic of Korea
[11]Centre for Research & Technology Hellas (CERTH), Chemical Process and Energy Resources Institute (CPERI), 52 Egialias str, Maroussi, Athens 15125, Greece
[12]School of Science and Technology, Hellenic Open University, Parodos Aristotelous 18, Patras 26335, Greece

‡ These authors contributed equally to the work
*Corresponding Author: wcjung@snu.ac.kr ; mgoula@uowm.gr ; mascotto@uni-koblenz.de

KEYWORDS

exsolution; catalyst regeneration; dry reforming; ethane dehydrogenation; strong metal-support interaction

Supplementary Figures

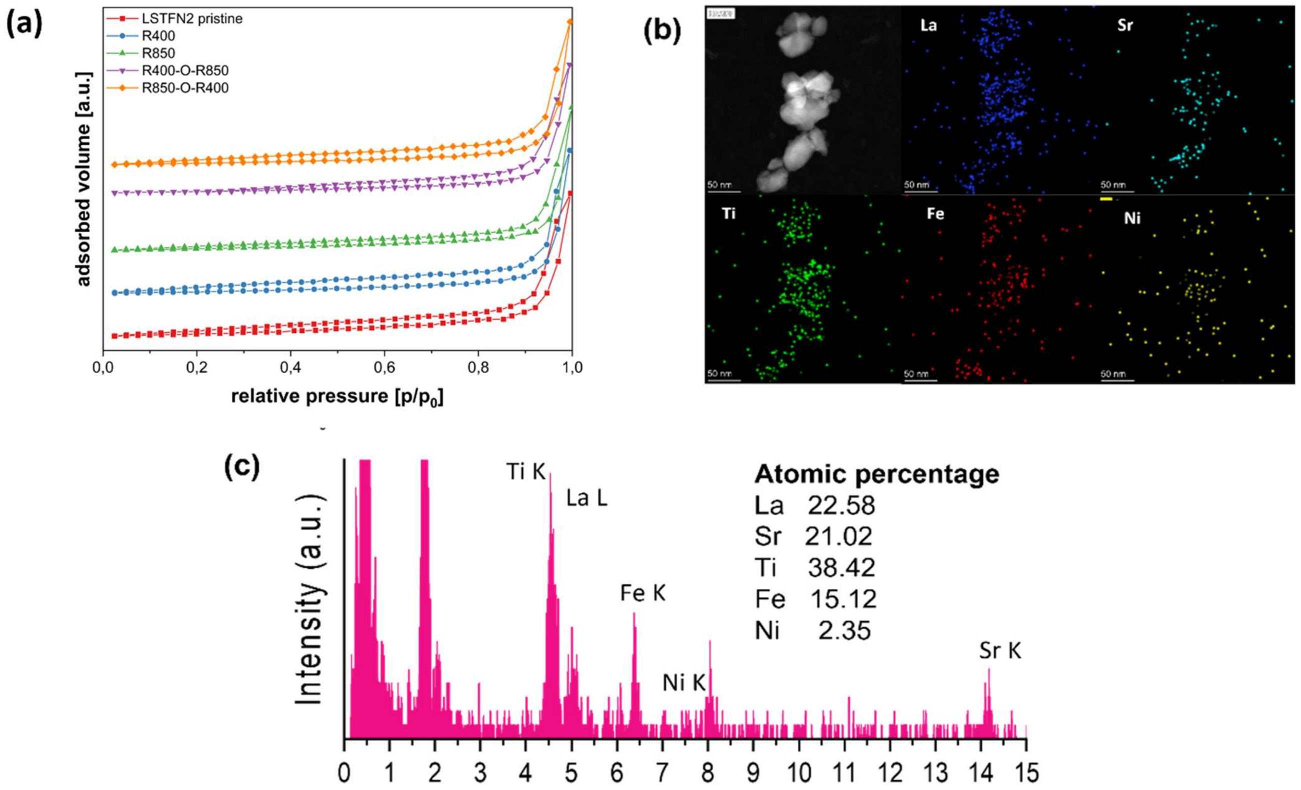

Supplementary Figure 1. Porosity and composition analysis of LSTFN2. (a) N_2-physisorption isotherm of the pristine and exsolved materials; (b) HAADF picture, HAADF-EDX mapping and (c) EDX spectrum of the as-synthesized LSTFN2 material

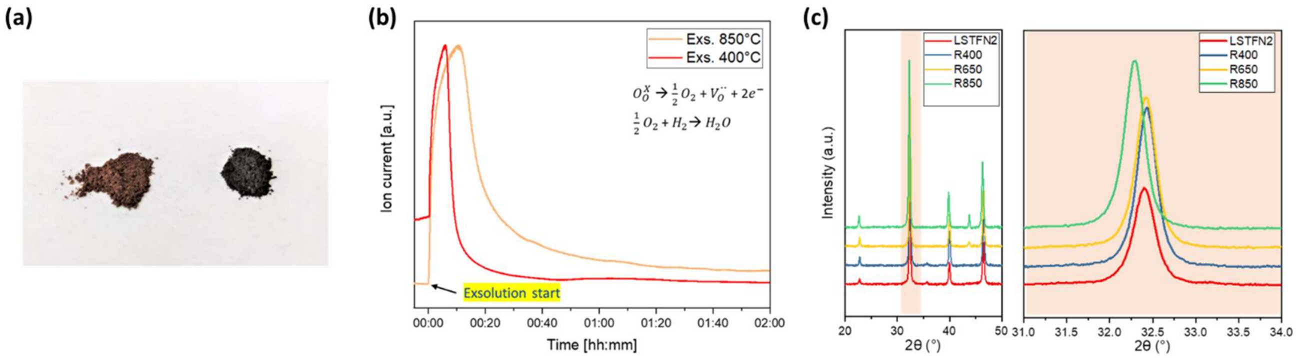

Supplementary Figure 2. **Insights into the LSTFN2 reduction**. (a) Picture of the pristine LSTFN2 material before (left) and after exsolution (right) at 400 °C for 3 h, under 5% H_2 in N_2 atmosphere; the blackening of the sample suggests a substantial reduction of the B-site species in the material; (b) mass spectrometry signals of showing water production due to the reduction of B-site metal cations at 400 °C and 850 °C as exsolution temperatures. The t=0 corresponds to the stage of injection of the reducing agent in the system, i.e. the starting of the exsolution process of the material; (c) Zoom on the (110) reflection of the pristine and exsolved LSTFN2 materials.

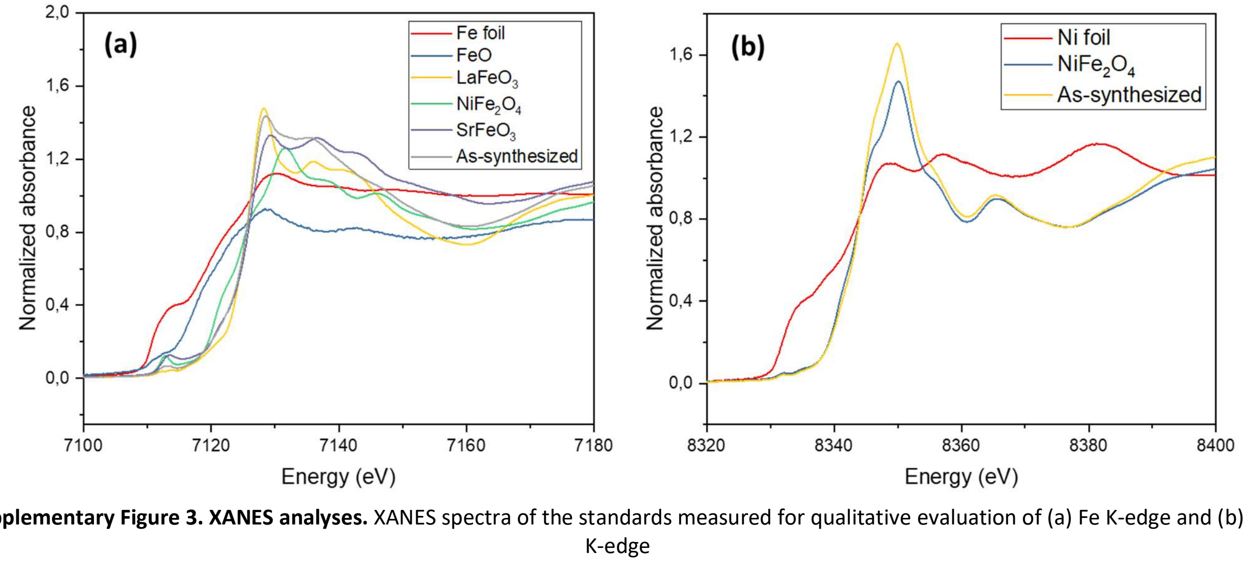

Supplementary Figure 3. XANES analyses. XANES spectra of the standards measured for qualitative evaluation of (a) Fe K-edge and (b) Ni K-edge

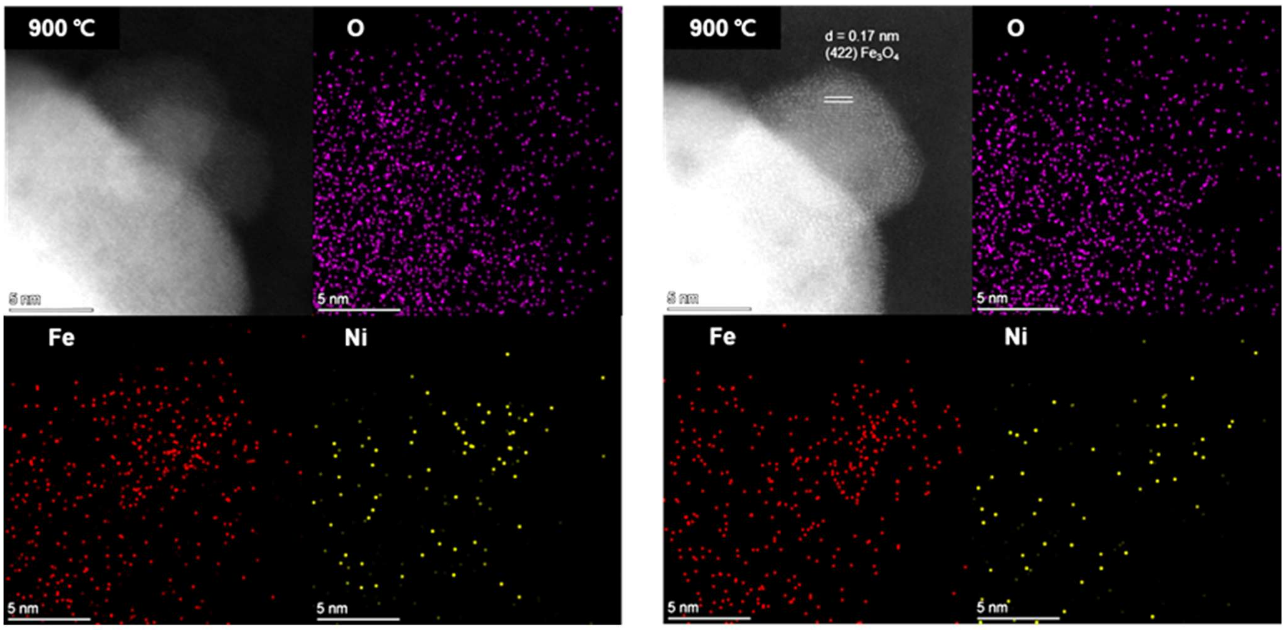

Supplementary Figure 4. TEM-EDX of LSTFN2. *In situ* HADDF-STEM and EDX mapping of the LSTFN2 material at 900 °C

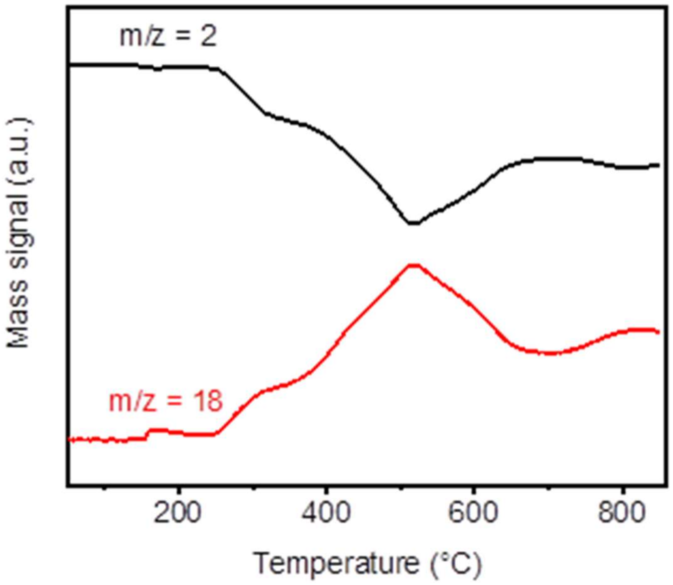

Supplementary Figure 5. Reduction profile of the LSTFN2 material under the H_2 atmosphere. Reduction of LSTFN2 under H_2 shows three H_2-consumption peaks at ~280, 400, and 710 °C (m/z = 2), attributed to exsolution associated with lattice-oxygen reduction in the subsurface region.

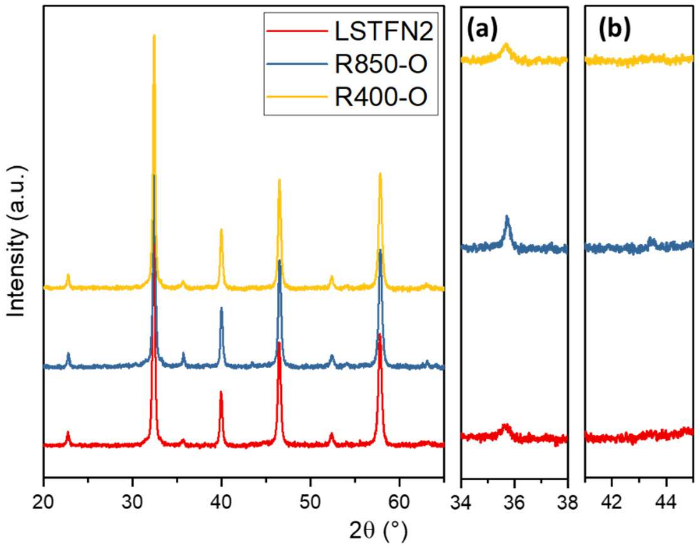

Supplementary Figure 6. XRD of the reduced perovskites after oxidation. Powder diffractograms of the re-oxidized samples, with focus on (a) the reflection range of the spinel phase and (b) that of the metallic nanoparticles.

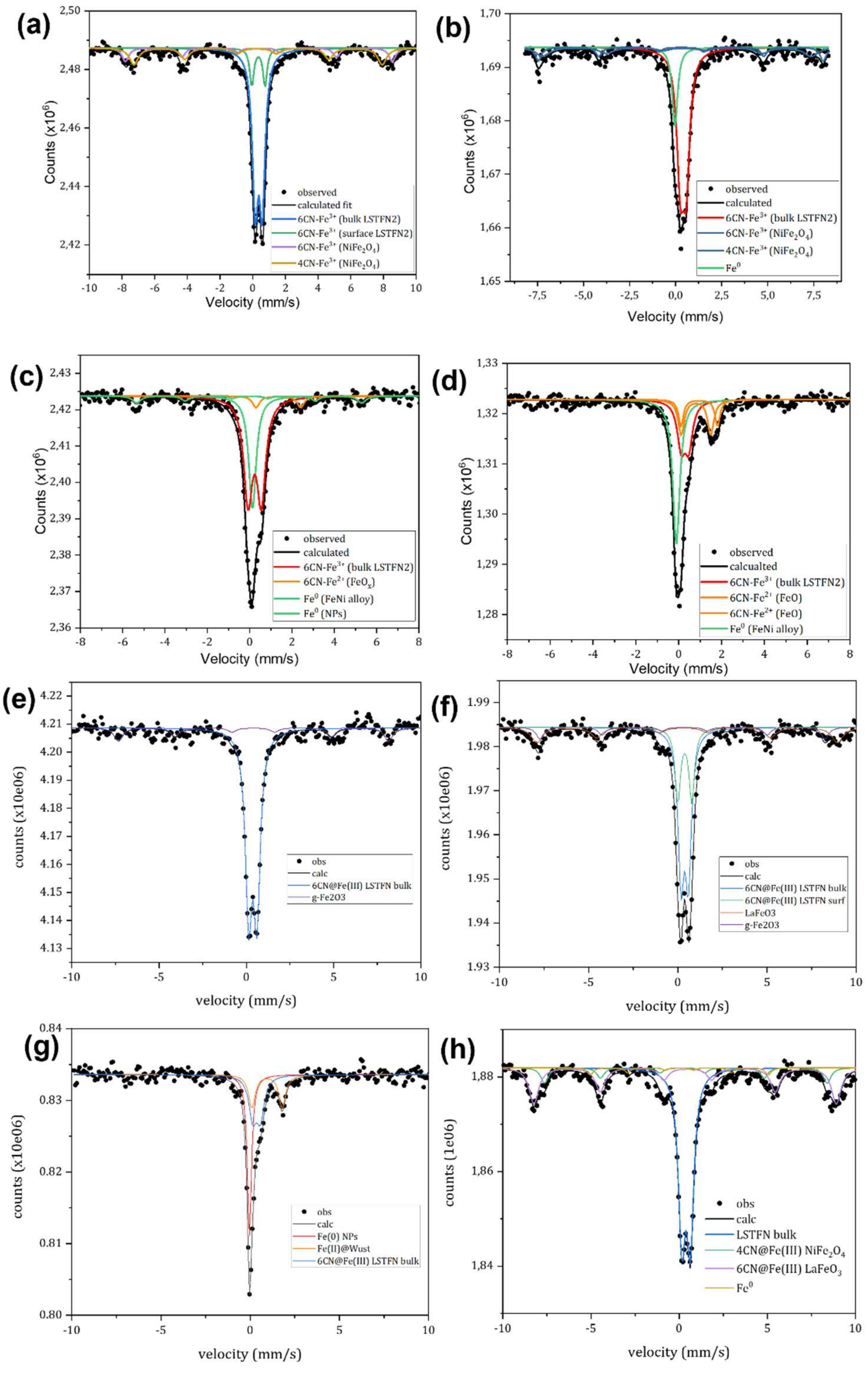

Supplementary Figure 7. Mössbauer spectra of the perovskite materials. Acquired Mössbauer spectra with the corresponding fittings for the samples LSTFN2 (a), R400 (b), R650 (c), R850 (d), R850-O (e), R400-O (f), R400-O-R850 (g), R850-O-R400 (h).

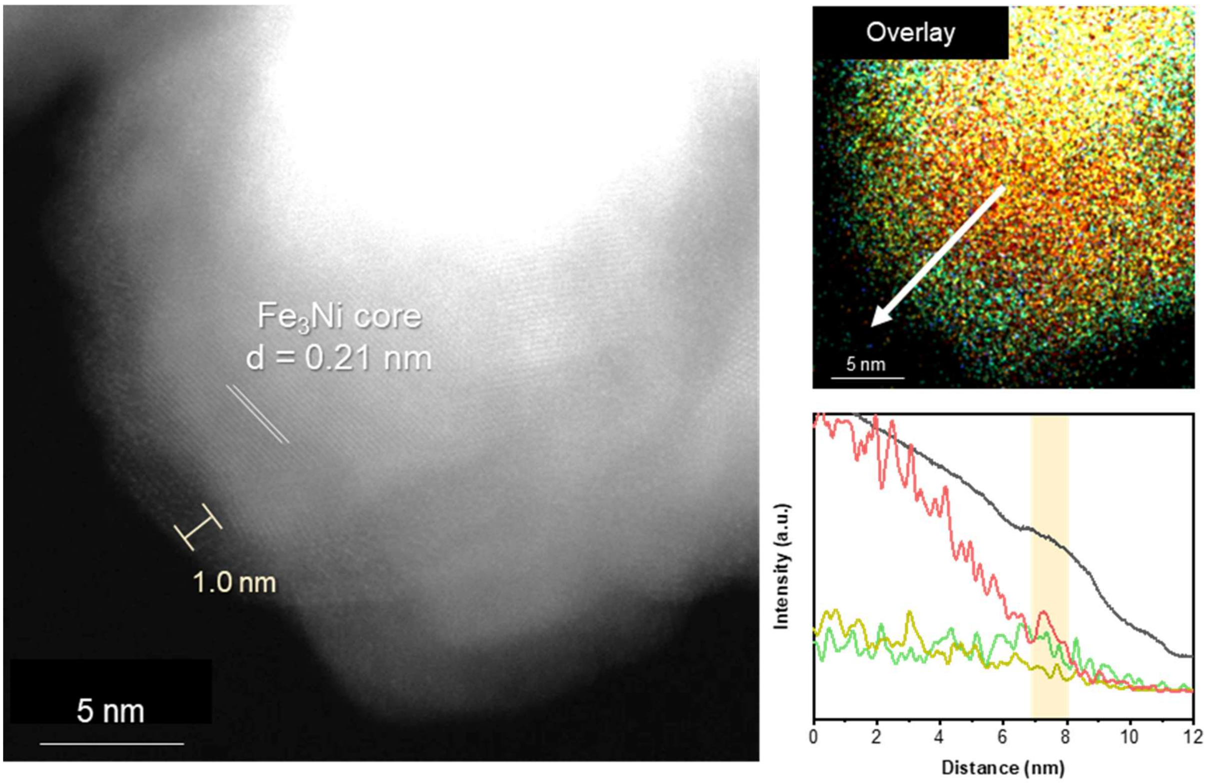

Supplementary Figure 8. Advanced electron microscopy analysis of R850-O-R400. HADDF-STEM EDX analysis result for R850-O-R400 and corresponding line profile of each element across the boundary area of the core-shell structure. The colored area represents the amorphous FeO_x shell domains, surrounding the alloy core.

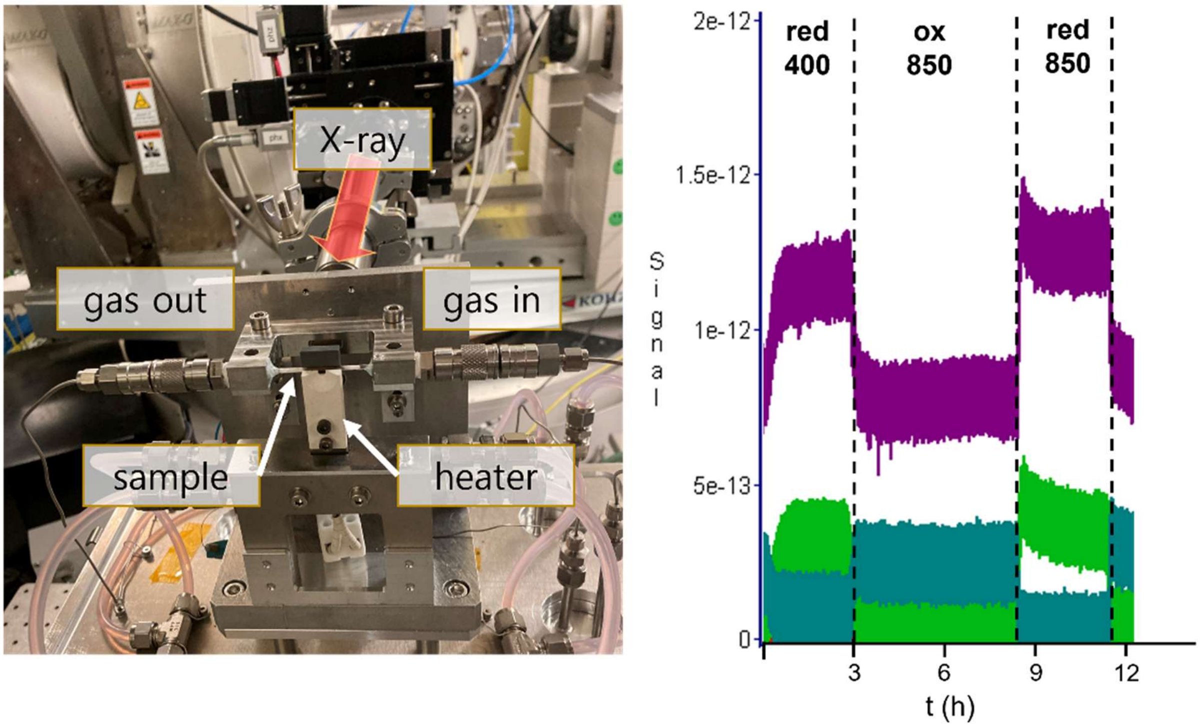

Supplementary Figure 9. In situ high-temperature cell and gas profiles. Picture of the high-temperature *in situ* cell (left) and gas profile of the mass spectrometer during the R400-O-R850 redox cycle (right). The purple curve indicates the concentration of N_2, the light green indicates H_2 and the dark green curve indicates the signal of O_2.

Details on the i*n situ* synchrotron XRD experiment

The exsolution and redissolution procedures of LSTFN2 powder sample were tracked following two protocols: 1) R850-O-R400: The sample was heated to 850 °C under synthetic air (10 mL min^{-1}) with a heating rate of 10 ^{o}C min^{-1}. Once the temperature was reached, the atmosphere was switched to 4% H_2 in N_2. The gas flow was initially 10 mL min^{-1} for ~20 minutes and then increased to 20 mL min^{-1} for a 3-hour reduction period. The gas was then switched back to synthetic air (20 mL min^{-1}) for a 5-hour reoxidation at 850 °C. Finally, the sample was cooled to 400 °C at 10 °C min^{-1} in air and subjected to a second reduction in 4% H_2 in N_2 (20 mL min^{-1}) for 3 hours before cooling to room temperature. 2) R400-O-R850: The sample was heated to 400 °C in synthetic air (20ml min^{-1}) with 10 °C min^{-1}. At 400 °C, the gas was switched to 4% H_2 in N_2 (20 mL min^{-1}) for a 3-hour reduction. The atmosphere was then switched back to air (20 mL min^{-1}), and the temperature was raised to 850 °C for a 5-hour reoxidation. Finally, while maintaining 850 °C, the gas was switched to 4% H_2 in N_2 (20 mL min^{-1}) for an additional 3-hour reduction.

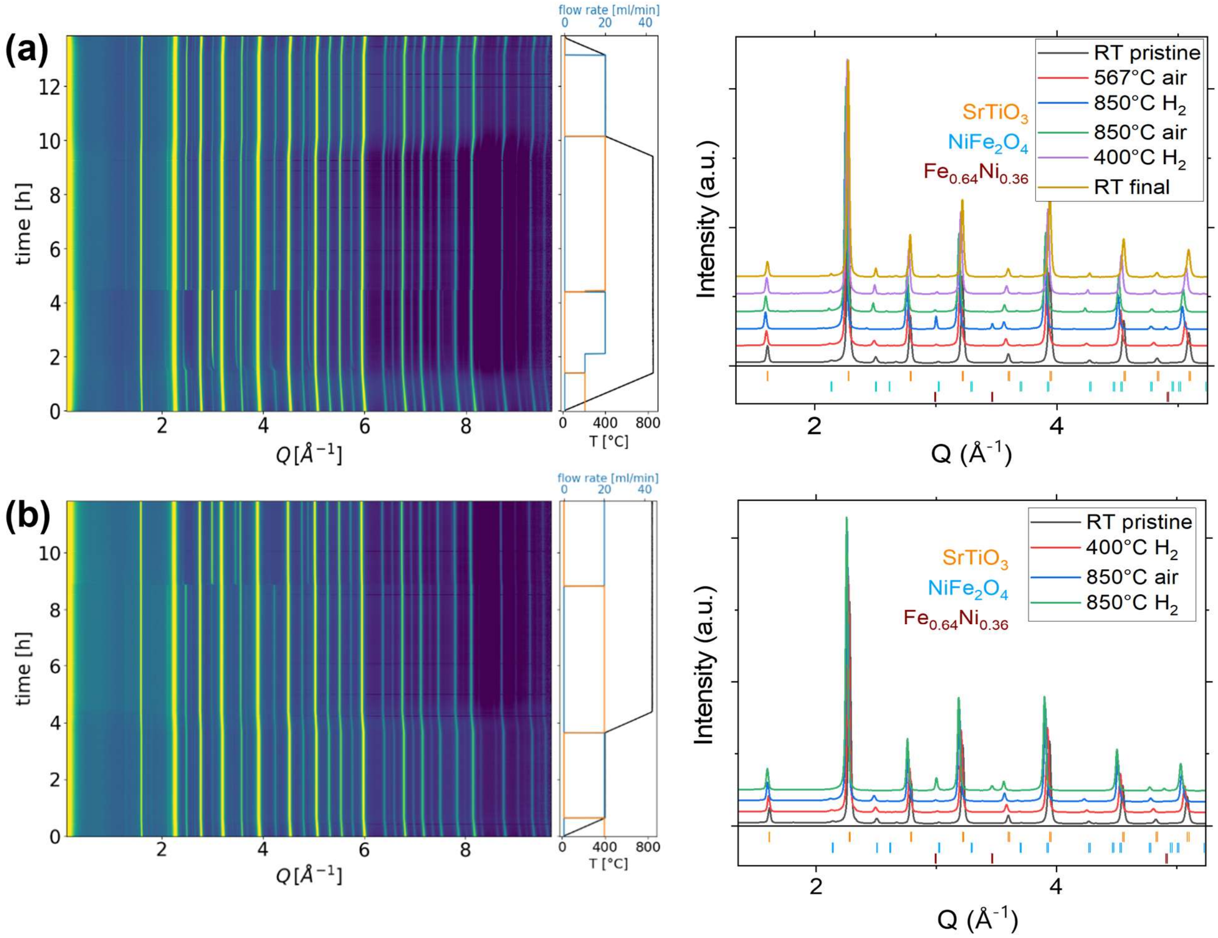

Supplementary Figure 10: **In situ XRD studies of the shapeshifting process**. (a) R850-O-R400 and the (b) R400-O-R850 during the redox cycles of LSTFN2 along with the respective XRD curves at each redox stage. The flow rate of air and H_2 in N_2 is shown in orange and blue, respectively. The temperature profile is marked in black.

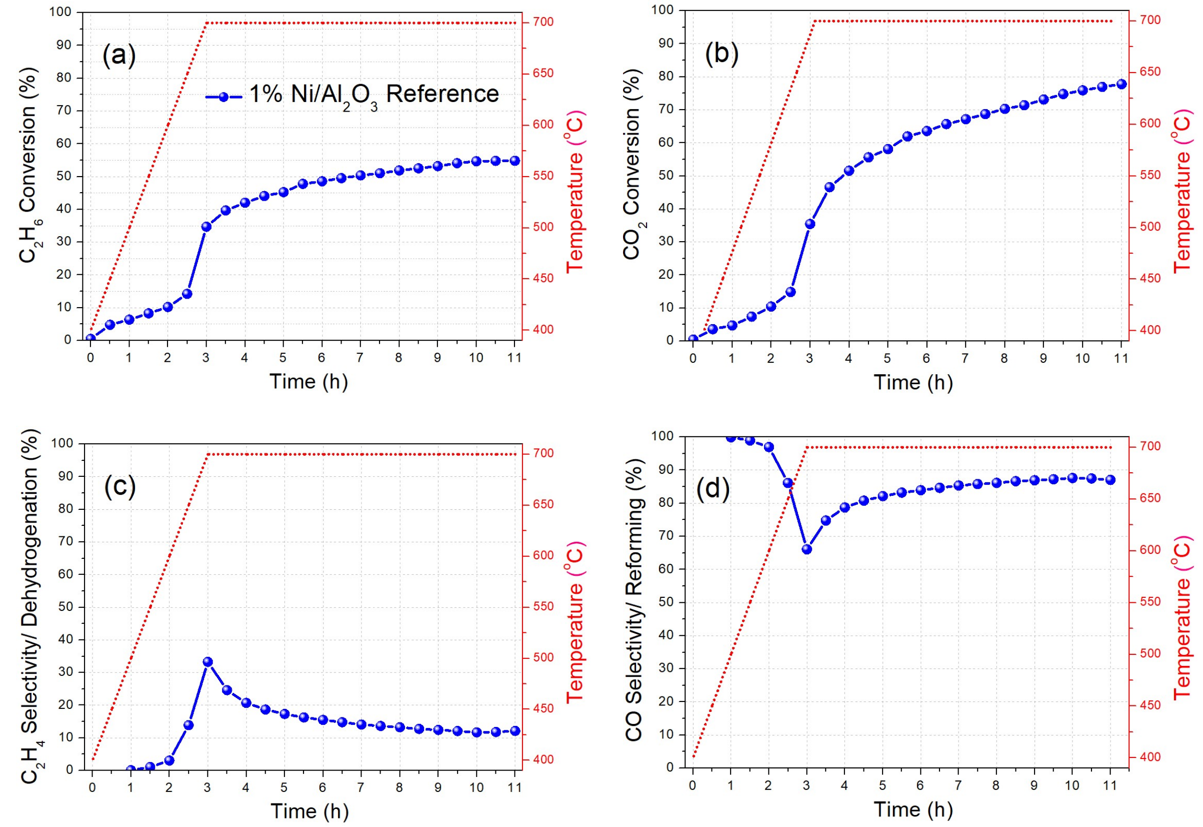

Supplementary Figure 11. Long-term catalytic test of the reference catalyst. (a) C_2H_6 conversion, (b) CO_2 conversion, (c) C_2H_4 selectivity (dehydrogenation), and (d) CO selectivity (reforming) of the 1% Ni/Al_2O_3 reference catalyst during the catalytic testing consisting of 3 h of temperature ramping (400 to 700°C) and 8 h of time-on-stream (700 ^{o}C constant).

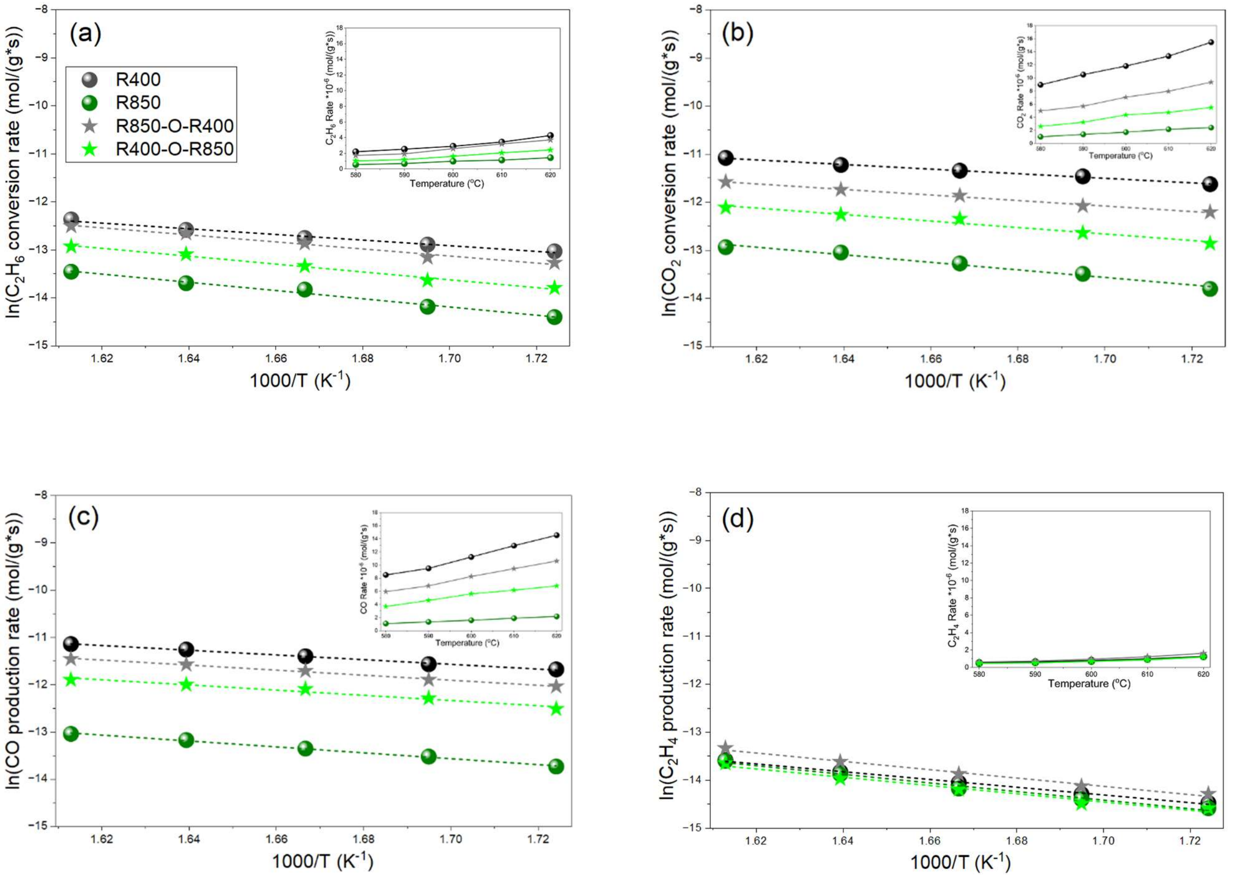

Supplementary Figure 12. Arrhenius plots for the calculation of the apparent activation energies. Natural logarithm of the consumption rates of C_2H_6 (a) and CO_2 (b), and the production rates of CO (c) and C_2H_4 (d) versus reciprocal temperature. The corresponding rates versus temperature plots are presented as insets.

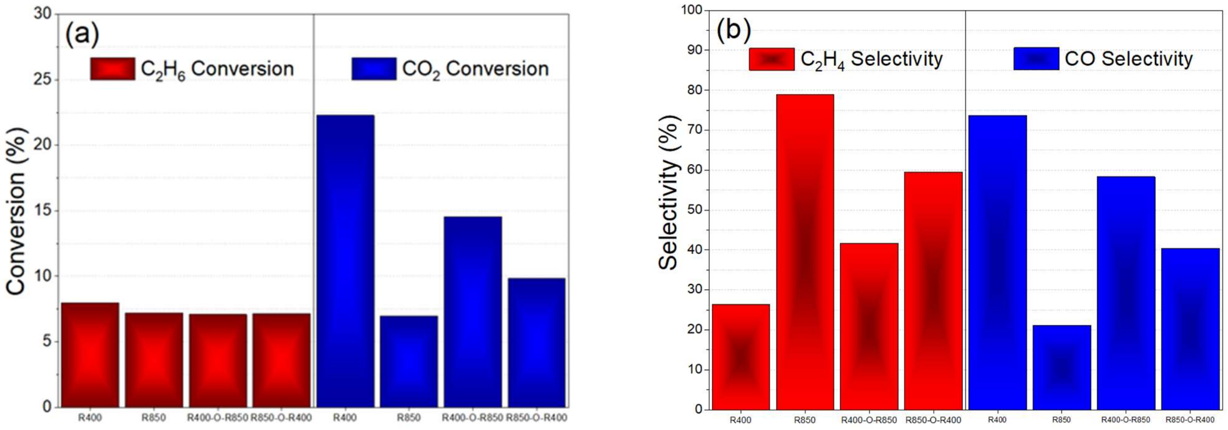

Supplementary Figure 13. Catalytic conversion and selectivity charts. Comparison of C_2H_6 and CO_2 conversions (a), as well as C_2H_4 and CO selectivities (b) at similar C_2H_6 conversion levels (7-8%).

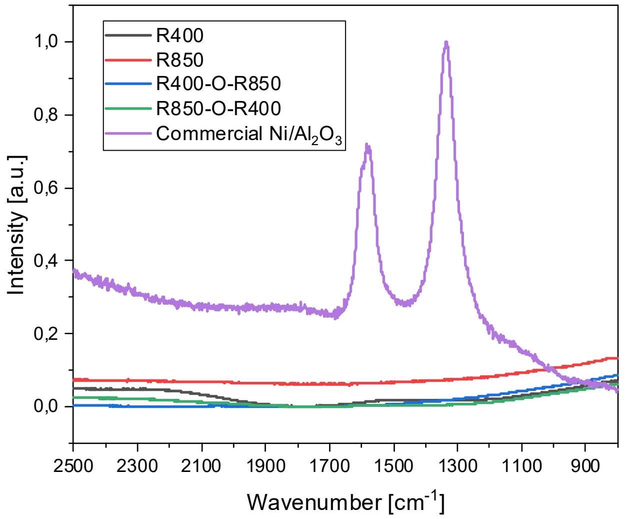

Supplementary Figure 14. Spectroscopic analysis of the materials after catalysis. Raman spectra of the spent perovskite-based and commercial catalysts.

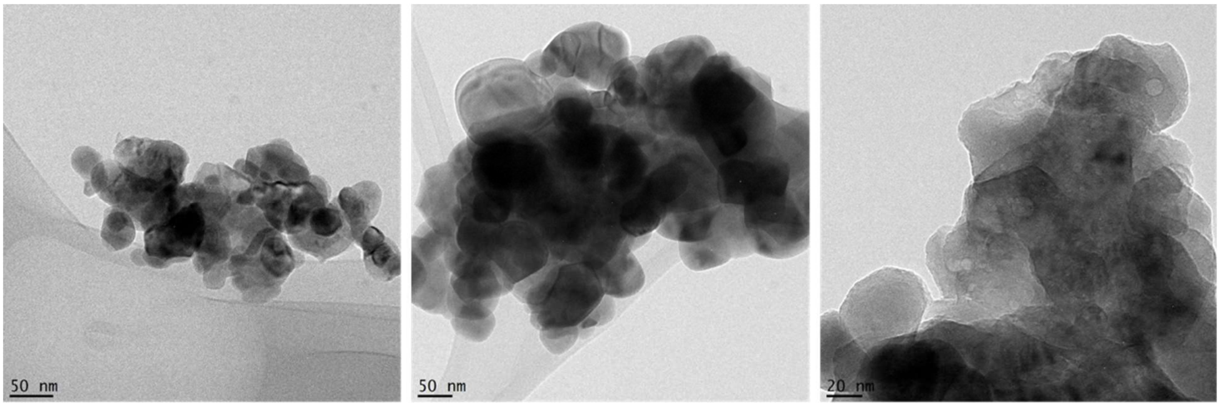

Supplementary Figure 15. Electron microscopy analysis of R850 after catalysis. TEM micrographs of the spent R850 material.

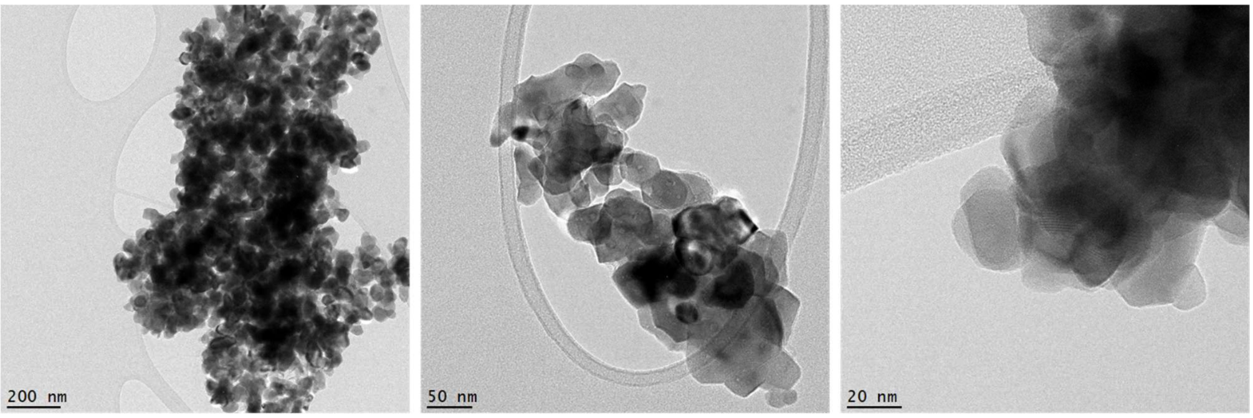

Supplementary Figure 16. Electron microscopy analysis of R400 after catalysis. TEM micrographs of the spent R400 material.

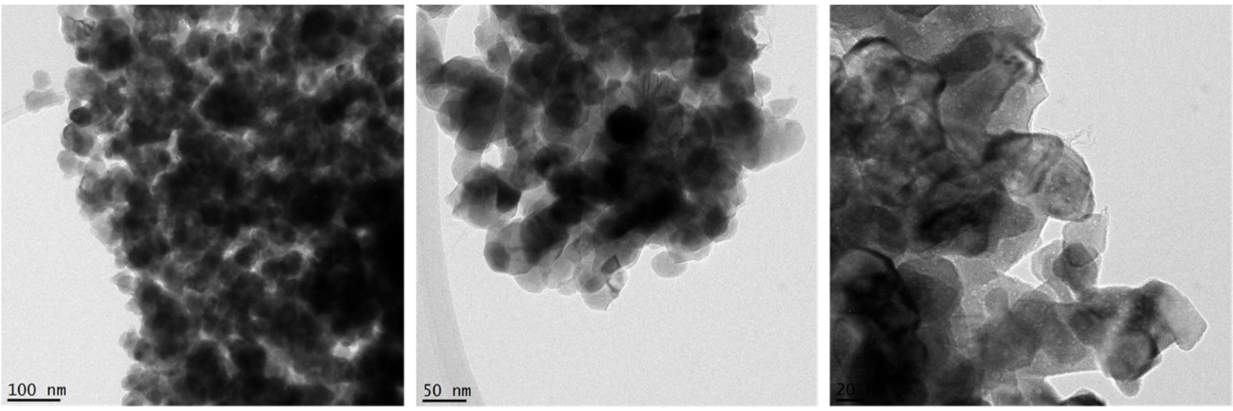

Supplementary Figure 17. Electron microscopy analysis of R400-O-R850 after catalysis. TEM micrographs of the spent R400-O-R850 material.

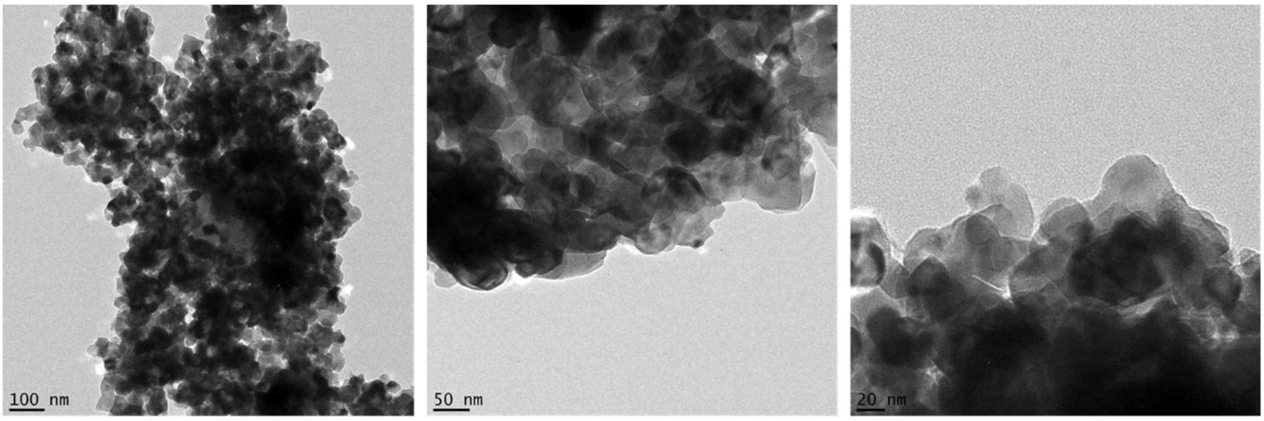

Supplementary Figure 18. Electron microscopy analysis of R850-O-R400 after catalysis. TEM micrographs of the spent R850-O-R400 material.

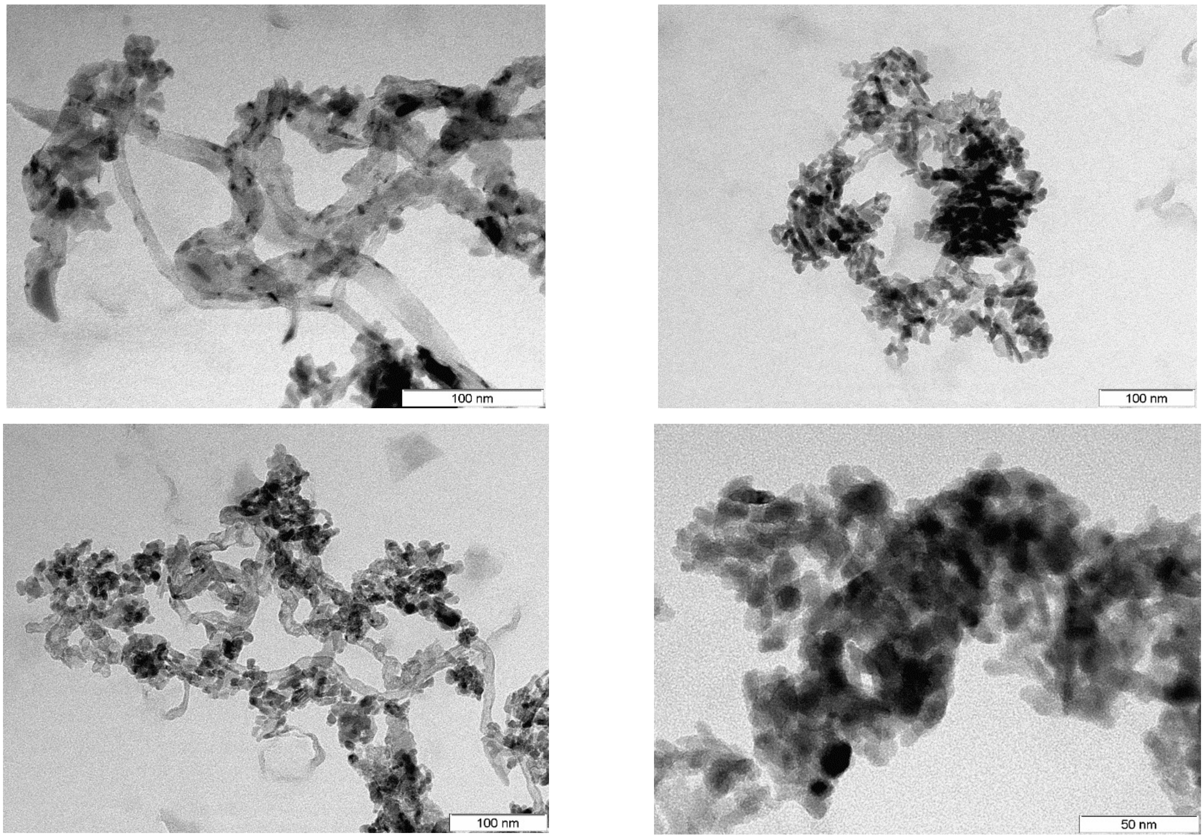

Supplementary Figure 19. Electron microscopy analyses of the reference catalyst. TEM micrographs of the spent Ni/Al_2O_3 catalyst. The formation of carbon baffles is clearly visible.

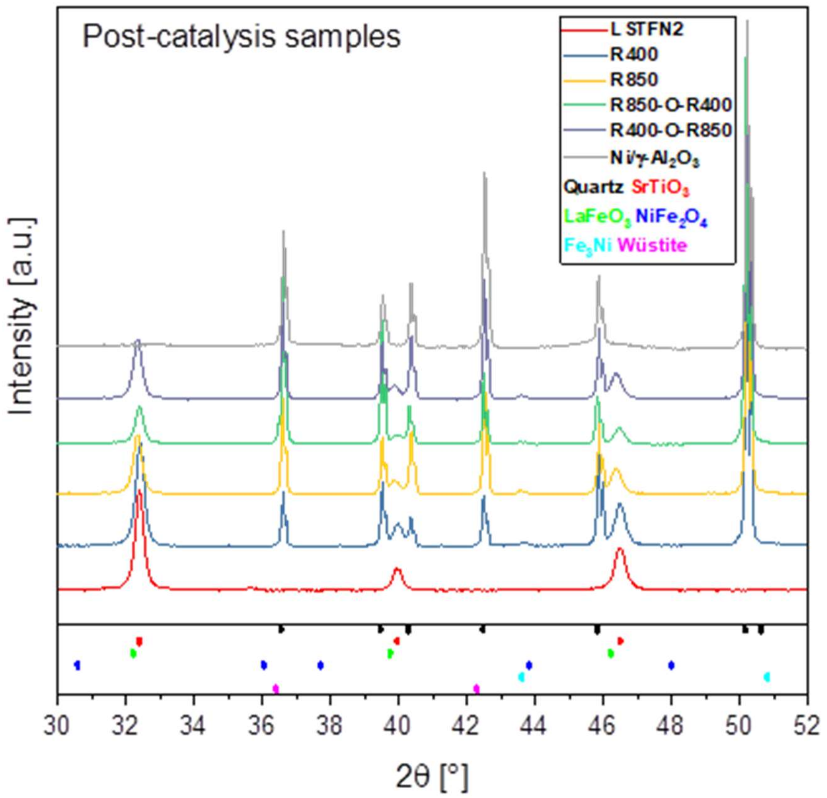

Supplementary Figure 20. XRD analyses of the spent catalysts. Post-catalysis XRD phase analysis of the spent materials. Measurement taken in the 2θ range between 30 and 55°.

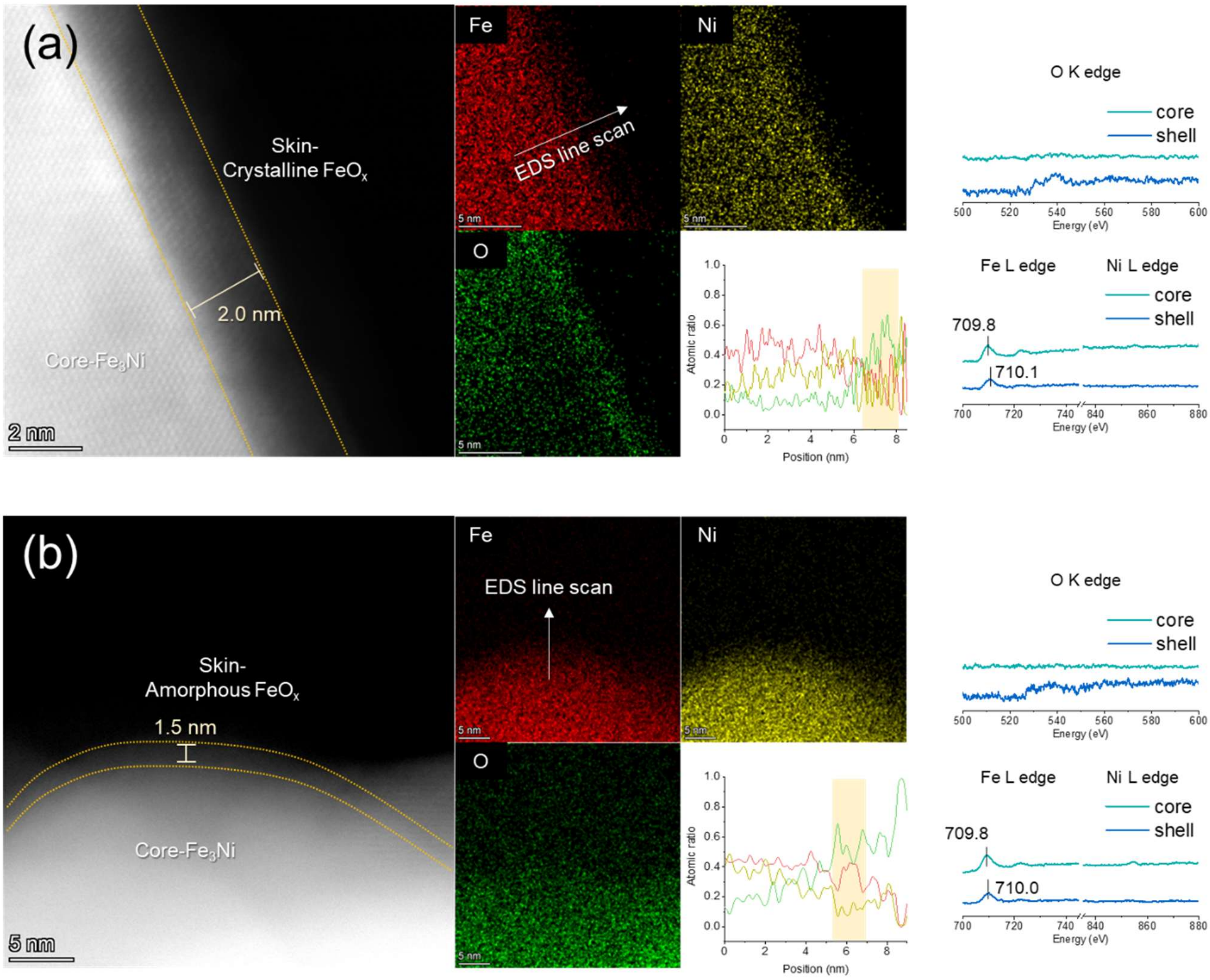

Supplementary Figure 21. Advanced electron microscopy analyses of the spent catalysts. HADDF-STEM images and corresponding EDX and EELS results on spent (a) R400-O-R850, and (b) R850-O-R400 (post-catalysis).

Supplementary Tables

Supplementary Table 1. Mössbauer hyperfine parameters of all of the examined materials, including the attributions given from the fitting of the spectra. The ones marked with * are the magnetically coupled species.

Sample	*d* (mm/s)	*Δ/e* (mm/s)	Γ_+ (mm/s)	*H* (T)	Area (%)	Attribution
**LSTFN2**	0.36	0.44	0.21		58	LSTFN2 bulk
	0.34	0.82	0.15		10	LSTFN2 surface
	0.38	-0.01	0.23	50.6	12	6CN-Fe(III)
	0.28	0.04	0.32	47.1	20	4CN-Fe(III)
**R400**	0.41	0.32	0.22		57	LSTFN2 bulk
	-0.04		0.18		15	NPs (Fe, FeNi)
	0.29	-0.015	0.20	47.96	11	6CN-Fe(III)
	0.26	-0.20	0.45	45.2	17	4CN-Fe(III)
**R650**	0.13±0.01		0.21		28	NPs (Fe, FeNi)
	0.24±0.01	0.63±0.04	0.25		60	LSTFN2 bulk
	1.3±0.1	2.1±0.2	0.20		5	Fe(II)
	0	-0.02±0.09	0.20		7	Fe(0)
**R850**	0.30	0.35	0.22		28	LSTFN2 bulk
	0.95	1.69	0.15		11	4CN-Fe(II)
	0.80	1.38	0.17		18	4CN-Fe(II)
	-0.11		0.20		43	Fe(0) or FeNi
**R400-O**	0.38	0.41	0.20		49	LSTFN2 bulk
	0.38	0.78	0.18		22	LSTFN2 surface
	0.43	0.11	0.38	52.2	19	$LaFeO_3$
	0.32	-0.06	0.25	49.6	10	γ-Fe_2O_3
**R850-O**	0.37	0.47	0.23		84	LSTFN2 bulk
	0.40	0.01	0.29	47.9	16	γ-Fe_2O_3
**R400-O-R850**	0.36	0.43	0.26		35	LSTFN2 bulk
	0.94	1.71	0.23		25	4CN-Fe(II)
	-0.04		0.15		40	Fe(0), FeNi
**R850-O-R400**	0.38	0.48	0.24		53	LSTFN2 bulk
	0.27	0.09	0.25	49.7	11	γ-Fe_2O_3
	0.41	-0.07	0.32	53.2	32	$LaFeO_3$
	0* (constraint)	0* (constraint)	0.2*	31.3	4	NPs (Fe, FeNi)

LSTFN2

The Mossbauer spectrum of pristine material consists of an intense paramagnetic absorption, centered around ≈0.36 mm/s, together with a broad sextet, attributable to the presence of magnetically coupled species. The best fitting was achieved by using four components: two paramagnetic doublets and two sextets. Concerning the two doublets, the obtained hyperfine parameters are typical of ferric ions in distorted octahedral environments. The two ferric sites differ each other for the Δ values, indicating that the paramagnetic ferric population

is distributed over two different distorted sites. The former, with the lowest *Δ* (0.44mm/s), could be representative of ferric sites hosted in the perovskite lattice. The latter exhibits a rather high *Δ* value (0.82 mm/s) could be representative of Fe(III) sites on the particle surface. Contrary to the previously studied LSTFN2 system, pristine material does not exhibit no signal attributable to Fe(IV) moieties. This evidence can be ascribed to the high calcination temperature. The broad sextet, due to magnetically coupled species, arises from the presence of a spinel structure. As matter of fact, the hyperfine parameters of the two sextets can be attributed to the octahedral (*Δ*: 0.38 mm/s and *H*: 50.6T) and tetrahedral (*Δ*: 0.28 mm/s and *H*: 47.1T) sites of a partially inverse AB_2O_4 lattice, as $NiFe_2O_4$. The broad linewidth of both sites (0.23 and 0.32 mm/s respectively) suggests a certain disorder around the ferric sites, as consequence of different local environments.

R400

The 400°C reduction promotes a drastic transformation of the system. The spectrum is dominated by a broad, intense, and asymmetric absorption, centered around ≈0.28 mm/s, together with a tiny magnetically coupled component. The spectrum was fitted with 4 components: a singlet, centered around 0 mm/s velocity value, a doublet, exhibiting a *Δ* close to 0.4 mm/s, and two weak sextets. As in pristine materials, the doublet can be ascribed to distorted ferric sites hosted in LSTFN2 lattice: it shows hyperfine parameters close to those obtained for the pristine materials: a low *Δ*, typical of distorted 6CN sites in LSTFN2 structures. Concerning the singlet, it shows an isomer shift close to 0 (-0.04 mm/s), indicating the presence of electron rich Fe nuclei. It can be reasonably ascribed to metallic iron in superparamagnetic regime. The simultaneous presence of a Fe:Ni alloy, in superparamagnetic regime, cannot be excluded at priori, because of the superimposition of the two signals, as suggested by the rather high singlet linewidth (Γ_+: 0.17mm/s). Concerning the weak magnetically coupled component, which represents the 28% of the total area, it can be tentatively fitted by using two sextets, which parameters are compatible with octahedral and tetrahedral sites in partially inverted spinel structure. It is interesting to observe that the intensity of doublet is close to the one obtained in pristine materials. This evidence points out that the bulk LSFN structure is not strongly affected by the reduction process.

R650

Increasing the reduction temperature to 650 °C the metallic phase (identified by a singlet and a sextet) increased to 35% of the total amount of iron. In addition, a further contribution, assigned to Fe^{2+} species in octahedral coordination, is observed thus indicating the presence of iron oxides by 5% formed during the course of the reduction process.

R850

The spectrum of the reduced sample shows an intense and broad absorption centered around zero velocity together with a weaker one, centered at ≈1.4 mm/s. The best fit was obtained by using a four components model, consisting in a singlet and three doublets. After the reduction, 72% of the total iron is reduced and distributed over metallic NPS and a wustite lattice. As matter of fact, similarly to R400, the singlet shows hyperfine parameters related to electron rich Fe nuclei; it is compatible with the presence of α-Fe and/or a Fe:Ni alloy in superparamagnetic regime. Concerning the two ferrous doublets, according to XANES data, can be tentatively attributed to ferrous sites in wustite lattice. The remaining 28% of the total Fe occupies a ferric site in distorted octahedral geometry, which hyperfine parameters are compatible with those of LSTFN2 bulk. It is worth observing that increasing the reduction temperature from 400°C to 850°C, the amount of metallic Fe increases dramatically from 15% to 43%. Moreover, the wustite formation seems to be preferred to the nickel ferrite one, highlighting that the increase in temperature facilitates the Fe reduction.

R850-O

The oxidation of the exsolved sample drastically transforms the Fe distribution. The spectrum is dominated by an intense, symmetric, paramagnetic absorption and a weak magnetically coupled component. The best fitting was achieved by a two components model: one doublet and one sextet. Again, the doublet can be representative of ferric nuclei in distorted octahedral geometry. It shows hyperfine parameters close to the pristine one, that it can be related to Fe(III) hosted in LSTFN2 lattice. Every attempt to fit the spectrum with two paramagnetic components did not allow for a satisfactory result. The magnetic component, populated by ≈13% of the total Fe, consists of a single weak sextet which hyperfine parameters are compatible with those of maghemite, γ-Fe_2O_3 and/or poorly crystalline hematite. As discussed in R400-O, the presence of maghemite could be consistent with the Mössbauer spectrum of R850.

R400-O

The high temperature oxidation logically promotes the complete oxidation of the reduced species. The best fit of the spectrum consists of two doublets and two sextets. The sextets are representative to ferric nuclei in distorted octahedral geometry. Both sites show hyperfine parameters close to those of the pristine material. The one with the lowest Δ (0.41 mm/s) is attributable to the ferric sites hosted in LSTFN2 structure, while the one with the highest Δ (0.78 mm/s) can be ascribed to ferric sites on the surface of the LSFTN2 particles. The two sextets are both representative of octahedral ferric sites in oxides. Considering the obtained hyperfine parameters, the sextet exhibiting the highest H value, 52.2 T, can be ascribed to Fe(III) in $LaFeO_3$, while the other one can be tentatively attributed to Fe(III) in maghemite, γ-Fe_2O_3 and/or poorly crystalline hematite, α-Fe_2O_3. Even if the distinction between the two ferric oxides is quite straightforward in case of pure, highly crystalline sample, the discrimination can be rather difficult when the species move from the ideality. Both oxides host an octahedral ferric site in which hyperfine parameters are very sensitive to the local environment. Maghemite, a cation-deficient inverse spinel, shows a H near 49.9T and a null quadrupole shift while hematite, a corundum type oxide, a considerably higher H (51.7T) and a negative quadrupole shift, as consequence of its weakly ferromagnetic nature. A poor crystallinity in hematite promotes an increase in Δ and a decrease in H, making the distinction between the to oxide quite impossible. Considering the presence of an inverse spinel structure in both pristine materials and R400 the formation of maghemite can be supposed.

R400-O-R850

The best fitting was obtained by using a three components model, consisting in one singlet, and two doublets. In general, the high temperature reduction promotes the disappearance of all the magnetically coupled components and the formation of Fe(II) and Fe(0) species. Only 35% of the total Fe isn't affected by the reduction: it is located a single distorted octahedral site, which parameters are compatible with those of the ferric sites hosted in LSTFN2 lattice. The reduction promotes the formation of consistent amount, 40%, of α-Fe and Fe:Ni alloy, represented by the broad singlet centered at -0.04 mm/s, together with a minor amount of paramagnetic ferrous species. The latter shows hyperfine parameters typical of distorted tetrahedral Fe(II) sites, compatible with Fe hosted in defected wüstite lattice, according to XRD data.

R850-O-R400

The reduction at 400°C seems to promote the formation of a consistent amount of magnetically ordered phases. The spectrum shows the presence of a broad magnetic pattern together with a narrow absorption, centered at ≈0.4 mm/s: The best fitting was achieved by three component model: one doublet and two sextets. Again, the doublet hyperfine parameters are consistent with the distorted octahedral ferric sites hosted in LSTFN2. The two sextets show hyperfine parameters are related to a partially inverse spinel. In addition to that, a small amount of metallic Fe phase is observed from the fitting. It is worthy to note that, the quite huge linewidth of the two sites can be representative of a certain disorder in the Fe local environment or can be significant of the superimposition of several components with similar hyperfine parameters, not distinguishable in the RT spectrum.

Supplementary Table 2. Summary of the catalytic performance results (reaction metrics, Figure 7) following 11 hours of reaction, i.e., 3 h of temperature ramping (400 to 700°C) and 8 h of time-on-stream (700 °C constant).

Material	Conversion (%)		Yield (%)				Selectivity (%)			H_2/CO	CO uptake (mmol/g)
	C_2H_6	CO_2	C_2H_4	CO	H_2	CH_4	C_2H_4	CO	CH_4		
R400	32.6	67.6	6.8	29.2	17.3	0.3	20.8	78.3	0.9	0.58	$1.66 *10^{-3}$
R850	22.1	30.2	16.6	12.1	6.0	0.3	75.2	23.6	1.2	0.49	$1.36 *10^{-3}$
R850-O-R400	23.9	37.1	11.1	13.9	7.7	0.2	46.3	52.9	0.8	0.55	-
R400-O-R850	20.9	28.8	13.9	11.0	5.7	0.2	66.7	32.6	0.7	0.52	-

Supplementary Table 3. Catalytic performance comparison between R400, R850, and other reported catalysts in the literature for the respective reactions of CO_2-assisted oxidative dehydrogenation of ethane (ODH) and dry ethane reforming (DER).

**1) CO_2-assited oxidative dehydrogenation of ethane (ODH)**			
**Catalyst**	**Reaction conditions**	**Catalytic Performance**	**Ref.**
R850 ($La_{0.4}Sr_{0.40}Ti_{0.6}Fe_{0.35}Ni_{0.05}O_{3\pm\delta}$ with exsolution at 850 °C)	WHSV = 24000 ml g_{cat}^{-1} h^{-1}<br>C_2H_6:CO_2 = 1:1<br>T = 700 °C (8 h)	$X_{C_2H_6}$ = 22.1%<br>X_{CO_2} = 30.2%<br>$S_{C_2H_4}$ = 75.2%	This work
Ni_1Fe_3/CeO_2	WHSV = 24000 ml g_{cat}^{-1} h^{-1}<br>C_2H_6:CO_2 = 1:1<br>T = 600 °C (11-13 h)	$X_{C_2H_6}$ = 3.5%<br>X_{CO_2} = 5.9%<br>$S_{C_2H_4}$ = 77.5%	[1]
1.0% PdFe (1:9)/ CeO_2	WHSV = 24000 ml g_{cat}^{-1} h^{-1}<br>C_2H_6:CO_2 = 1:1<br>T = 600 °C (11-12 h)	$X_{C_2H_6}$ = 6.6%<br>X_{CO_2} = 8.3%<br>$S_{C_2H_4}$ = 85.8%	[2]
NiFe/CeO_2-C1 (Ni:Fe = 1:4)	WHSV = 24000 ml g_{cat}^{-1} h^{-1}<br>C_2H_6:CO_2 = 1:1<br>T = 600 °C (12 h)	$X_{C_2H_6}$ = 4.6%<br>X_{CO_2} = 6.2%<br>$S_{C_2H_4}$ = 83%	[3]
$Fe_{1.5}Ni_{0.5}/ZrO_2$-M (ZrO_2 monoclinic)	WHSV = 9000 ml g_{cat}^{-1} h^{-1}<br>C_2H_6:CO_2 = 1:1<br>T = 700 °C (100 min)	$X_{C_2H_6}$ = 23.4%<br>X_{CO_2} = 28.0%<br>$S_{C_2H_4}$ = 80%	[4]
$Ti_{0.5}Ce_{0.5}O_2$	WHSV = 6000 ml g_{cat}^{-1} h^{-1}<br>C_2H_6:CO_2 = 1:1<br>T = 700 °C (8 h)	$X_{C_2H_6}$ = 32.3%<br>X_{CO_2} = 34.6%<br>$S_{C_2H_4}$ = 54.2%	[5]
Fe/NiO-MgO-ZrO_2 (5Fe/10NiMgZr, Fe-700 (5h))	WHSV = 7500 ml g_{cat}^{-1} h^{-1}<br>C_2H_6:CO_2 = 1:1<br>T = 650 °C (6.5 h)	$X_{C_2H_6}$ = 23%<br>X_{CO_2} = 25%<br>$S_{C_2H_4}$ = 90%	[6]
Fe/$MgAl_2O_4$ (Fe/3Mg7Al)	WHSV = 6000 ml g_{cat}^{-1} h^{-1}<br>C_2H_6:CO_2 = 1:1<br>T = 650 °C (60 min)	$X_{C_2H_6}$ = 21%<br>X_{CO_2} = 20%<br>$S_{C_2H_4}$ = 84%	[7]
$Fe_{1.5}Ni_{0.5}/CeO_2$	WHSV = 9000 ml g_{cat}^{-1} h^{-1}<br>C_2H_6:CO_2 = 1:1<br>T = 700 °C (2 h)	$X_{C_2H_6}$ = 20%<br>X_{CO_2} = 16%<br>$S_{C_2H_4}$ = 65%	[8]
$Fe_{1.5}Ni_{0.5}$/CeTi-C-2 (2% Ti substitution)	WHSV = 9000 ml g_{cat}^{-1} h^{-1}<br>C_2H_6:CO_2 = 1:1<br>T = 650 °C (1 h)	$X_{C_2H_6}$ = 10.6%<br>X_{CO_2} = 16.3%<br>$S_{C_2H_4}$ = 82.7%	[9]
$MgFeAlO_4$	WHSV = 9000 ml g_{cat}^{-1} h^{-1}<br>C_2H_6:CO_2 = 1:5<br>T = 600 °C (8 h)	$X_{C_2H_6}$ = 25.1%<br>X_{CO_2} = 22.1%<br>$S_{C_2H_4}$ = 92.8%	[10]
**2) Dry ethane reforming (DER)**			
**Catalyst**	**Reaction conditions**	**Catalytic Performance**	**Ref.**
R400 ($La_{0.4}Sr_{0.40}Ti_{0.6}Fe_{0.35}Ni_{0.05}O_{3\pm\delta}$ with exsolution at 400 °C)	WHSV = 24000 ml g_{cat}^{-1} h^{-1}<br>C_2H_6:CO_2 = 1:1<br>T = 700 °C (8 h)	$X_{C_2H_6}$ = 32.6%<br>X_{CO_2} = 67.6%<br>S_{CO} = 78.3%	This work
Ni_3Fe_1/CeO_2	WHSV = 24000 ml g_{cat}^{-1} h^{-1}<br>C_2H_6:CO_2 = 1:1<br>T = 600 °C (11-13 h)	$X_{C_2H_6}$ = 16.7%<br>X_{CO_2} = 41.3%<br>S_{CO} = 98.6%	[1]
$LaFe_{0.9}Ni_{0.1}O_3$	WHSV = 24000 ml g_{cat}^{-1} h^{-1}<br>C_2H_6:CO_2 = 1:1<br>T = 600 °C (12-14 h)	$X_{C_2H_6}$ = 21.4%<br>X_{CO_2} = 49.7%<br>S_{CO} = 97.5%	[11]
PtNi/CeO_2 (Pt:Ni = 1:3)	WHSV = 24000 ml g_{cat}^{-1} h^{-1}	$X_{C_2H_6}$ = 22.8%<br>X_{CO_2} = 53.4%	[12]

	$C_2H_6:CO_2$ = 1:1<br>T = 600 °C (12 h)	S_{CO} = 92.8%	
$Ni/MgAl_2O_4$	WHSV = 60000 ml g_{cat}^{-1} h^{-1}<br>$C_2H_6:CO_2$ = 1:2<br>T = 800 °C (18 h)	$X_{C_2H_6}$ = 96%<br>X_{CO_2} = 91%<br>S_{CO} = 93%	[13]
$Ni_{0.05}Ce_{0.975}O_2$	WHSV = 9000 ml g_{cat}^{-1} h^{-1}<br>$C_2H_6:CO_2$ = 1:1<br>T = 700 °C (2 h)	$X_{C_2H_6}$ = 63.9%<br>X_{CO_2} = 88.1%<br>S_{CO} = 94.3%	[5]
$CaZr_{0.8}Ni_{0.2}O_{3-\delta}$	WHSV = 15000 ml g_{cat}^{-1} h^{-1}<br>$C_2H_6:CO_2$ = 1:2<br>T = 700 °C (8 h)	$X_{C_2H_6}$ = 83%<br>X_{CO_2} = 82%<br>S_{CO} = 93%	[14]
FeNi/Ce-$Al_{0.5}$ (Fe:Ni = 3:1)	WHSV = 24000 ml g_{cat}^{-1} h^{-1}<br>$C_2H_6:CO_2$ = 1:1<br>T = 600 °C (8 h)	$X_{C_2H_6}$ = 11.7%<br>X_{CO_2} = 33.1%<br>S_{CO} = 97.7%	[15]
$NdFe_{0.7}Ni_{0.3}O_3$	WHSV = 60000 ml g_{cat}^{-1} h^{-1}<br>$C_2H_6:CO_2$ = 1:2<br>T = 600 °C (8-10 h)	$X_{C_2H_6}$ = 31.5%<br>X_{CO_2} = 42.4%<br>S_{CO} = 97.8%	[16]
Ni-2.4La@Si	WHSV = 36000 ml g_{cat}^{-1} h^{-1}<br>$C_2H_6:CO_2$ = 1:2<br>T = 700 °C (200 h)	$X_{C_2H_6}$ = 96%<br>X_{CO_2} = 86%<br>S_{CO} = 87%	[17]
Ni-Si@Ce	WHSV = 24000 ml g_{cat}^{-1} h^{-1}<br>$C_2H_6:CO_2$ = 1:2<br>T = 650 °C (10 h)	$X_{C_2H_6}$ = 70%<br>X_{CO_2} = 71%<br>S_{CO} = 97%	[18]

Supplementary Table 4. Reactant conversion and product formation rates following 11 hours of reaction, i.e., 3 h of temperature ramping (400 to 700 °C) and 8 h of time on stream (700 °C constant).

Material	Reactant conversion rate (μmol g_{cat}^{-1} min^{-1})		Product formation rate/ Space time yield (STY) (μmol g_{cat}^{-1} min^{-1})		
	C_2H_6	CO_2	C_2H_4	CO	H_2
R400	1357	2817	283	3650	2163
R850	921	1260	692	1513	750
R850-O-R400	995	1545	463	1738	963
R400-O-R850	870	1200	579	1375	713

Supplementary Table 5. Activation energy values for C_2H_6 and CO_2 conversion, as well as CO and C_2H_4 production calculated via the Arrhenius plots.

Material	$E_a(C_2H_6)$	$E_a(CO_2)$	$E_a(CO)$	$E_a(C_2H_4)$
R400	104	84	88	143
R850	151	138	110	158
R850-O-R400	129	101	94	153
R400-O-R850	144	119	97	152

Supplementary References:

[1] B. Yan, et al., Proc. Natl. Acad. Sci. 115 (2018) 8278–8283. https://doi.org/10.1073/pnas.1806950115.
[2] Z. Xie, et al., Chem. 6 (2020) 2703–2716. https://doi.org/10.1016/j.chempr.2020.07.011.
[3] M. Guo, et al., ChemCatChem. 13 (2021) 3119–3131. https://doi.org/10.1002/cctc.202100333.
[4] X. Li, et al., Fuel. 322 (2022) 124122. https://doi.org/10.1016/j.fuel.2022.124122.
[5] L. Li, et al., Appl. Catal. A, Gen. 635 (2022) 118565. https://doi.org/10.1016/j.apcata.2022.118565.
[6] M. Tasioula, et al., ACS Catal. 13 (2023) 2176–2189. https://doi.org/10.1021/acscatal.2c05338.
[7] Y. Zheng, et al., ACS Catal. 13 (2023) 11153−11163. https://doi.org/10.1021/acscatal.3c02029.
[8] X. Li, et al., Int. J. Hydrogen Energy. 79 (2024) 45–59. https://doi.org/10.1016/j.ijhydene.2024.06.398.
[9] X. Li, et al., Chem. Eng. J. 484 (2024) 149047. https://doi.org/10.1016/j.cej.2024.149047.
[10] Q. Du, et al., Carbon Capture Sci. Technol. 14 (2025) 100327. https://doi.org/10.1016/j.ccst.2024.100327.
[11] B. Zhao, et al., J. Catal. 358 (2018) 168–178. https://doi.org/10.1016/j.jcat.2017.12.012.
[12] Z. Xie, et al., Appl. Catal. B Environ. 245 (2019) 376–388. https://doi.org/10.1016/j.apcatb.2018.12.070.
[13] K.H. Kim, et al., J. CO_2 Util. 53 (2021) 101713. https://doi.org/10.1016/j.jcou.2021.101713.
[14] A.I. Tsiotsias, et al., J. CO_2 Util. 61 (2022) 102046. https://doi.org/10.1016/j.jcou.2022.102046.
[15] T. Zhang, et al., Engineering. 18 (2022) 173–185. https://doi.org/10.1016/j.eng.2021.11.027.
[16] Y. Wang, et al., Appl. Catal. B Environ. 337 (2023) 123010. https://doi.org/10.1016/j.apcatb.2023.123010.
[17] C. Wang, et al., Chem. Eng. J. 491 (2024) 152091. https://doi.org/10.1016/j.cej.2024.152091.
[18] X. Chen, et al., Int. J. Hydrogen Energy. 103 (2025) 501–512. https://doi.org/10.1016/j.ijhydene.2025.01.288.
