# Supplementary material for: Shapeshifting exsolved FeNi bimetallic nanostructures as catalytic switchers during the CO2-mediated ethane conversion
Source: Nat Commun. 2026 Apr 13;17:5117. doi: 10.1038/s41467-026-71282-6 (PMC13247154; doi:10.1038/s41467-026-71282-6)
Supplement: Supplementary file 2 — Transparent Peer Review file [file 41467_2026_71282_MOESM2_ESM.pdf]

nature portfolio

Peer Review File

Shapeshifting bimetallic nanoparticles with switching catalytic performance

Corresponding Author: Professor Simone Mascotto

This file contains all reviewer reports in order by version, followed by all author rebuttals in order by version.

Version 0:

Reviewer comments:

Reviewer #1

(Remarks to the Author)
Herein a reversible transformation between Fe_3Ni alloy nanoparticles and $Fe_3Ni@FeO_x$ core–shell structures was cleverly designed for the CO2-mediated ethane conversion. The results are interesting, and the characterization of the structure is adequate. This is of great significance for the promotion and application of smart catalysts based on perovskite structure. However, as the structural evolution of perovskite-based materials in reducing and oxidizing atmospheres has been reported, so the author should provide advanced insights in evolution of material structure and the effect of exsolution process on catalytic performances. There are two key issues: (1) The reason for the formation of shells on the alloy surface has not been explained clearly? The author only briefly discussed without providing sufficient experimental evidence; (2) There is a difference in activity between the two catalysts, and no further experimental evidence has been provided, mainly relying on the relationship between the structure and activity proposed in the reported literatures. Therefore, I recommend that this paper should be resubmitted after revision. The other comments are as follows:
1. The formation of a quite porous morphology is detected by transmission electron microscopy analysis. This is not enough, and the pore structure should be evaluated by nitrogen adsorption-desorption.
2. The bar of TEM and EELS spectroscopy in Figure 3 is not clear. Please modify.
3. The images of Figure 3C should be rotated to Intuitively display the thickness of thin amorphous shell.
4. The description of Figure 3e is absent. Please add the information.
5. In situ TEM experiments were performed under high-vacuum conditions to avoid the disturbing effect of the air exposure (Figure 4). In other word, H2 was absent, so that what's the role of H2 atmosphere during the exsolution process?
6. It was stated that EDXS results of the in situ TEM analyses show that the shell layer is composed of iron and oxygen only (Figure S4). Figure S4 should be corrected to Figure S3. Please check it. On the other hand, Accord to the EDXS results, the conclusion that iron and oxygen are enriched on the surface of particles is very inconclusive. Please provide more favorable EDXS evidence.
7. Two Figure 4 are in present manuscript. The numbering of the image is incorrect.
8. The author concluded that exsolved nanoparticles in R400 (Figure 3d) are covered with thin amorphous shell (~ 1 nm) of iron oxide (FeOx). However, in Figure 4c, the shell was absent in the picture of the shapeshifting mechanism. Why?
9. What's the ideal product and reaction routine for CO2-mediated ethane conversion? The activity of the R400 and R850 catalysts should be compared with the literature results, to highlight the novelty of exsolution process for designing multifunctional catalyst materials.

Reviewer #2

(Remarks to the Author)

The authors investigate dynamic redox behavior in layered double hydroxide (LDH)-derived NiFe catalysts for ethylene and CO_2 hydrogenation, focusing on the interplay between structural evolution and catalytic performance. Through a combination of operando XAS, in situ STEM, and transient measurements, they propose that redox cycles during activation and reaction influence selectivity and conversion.

The topic aligns well with the scope of Nature Communications. However, the core claims remain unconvincing due to oversimplification in the interpretation of catalytic performance. While the work touches on important themes in catalyst restructuring and redox chemistry, the manuscript does not provide a comprehensive discussion of prior studies in LDH-

derived systems or other dynamic metal-oxide catalysts in CO_2/ethylene hydrogenation. Several relevant works are overlooked, which limits the ability to understand the novelty and implications of the current findings. A stronger foundation in the literature would help frame the study's contributions and gaps more clearly.

The reaction evaluation, which should form the basis for understanding structure–function relationships, is underdeveloped. The authors primarily link catalytic behavior to exposure and conversion rather than through mechanistic insights. The treatment of selectivity is superficial, lacking a detailed breakdown of product distribution or reaction pathways. Without a thorough kinetic or mechanistic discussion, the proposed correlation between structural states and catalytic performance lacks depth and robustness. Key reaction parameters such as activation energy, turnover frequency, and product-specific rates are absent, further limiting interpretability.

The dynamic nature of the reaction conditions, particularly the linear temperature ramp toward steady state, presents an opportunity for extracting mechanistic insights. The manuscript does not fully exploit this aspect. Comparing catalyst behavior at equivalent conversion levels during the transient phase could reveal whether the observed performance differences stem from intrinsic activity or changes in redox state. Such analysis is absent, leaving ambiguity about whether the differences between catalysts are kinetic, structural, or purely linked to the accessible number of sites.

The RX-O-RY sample, which appears to have undergone a reduction-oxidation-reduction treatment, is not described clearly. It is implied that this sequence mimics potential in situ redox cycles during catalytic operation. However, the paper does not explore whether these transformations can occur under actual reaction conditions, such as in the presence of ethylene or CO_2. The lack of post-reaction characterization or detailed in situ monitoring under working conditions makes it difficult to establish whether the proposed redox cycling mechanism is relevant during catalysis. The stability and reversibility of the transformations under reaction conditions are not adequately supported.

Furthermore, the literature context is insufficiently developed. The title and abstract are very vague.

Reviewer #3

(Remarks to the Author)
I co-reviewed this manuscript with one of the reviewers who provided the listed reports. This is part of the Nature Communications initiative to facilitate training in peer review and to provide appropriate recognition for Early Career Researchers who co-review manuscripts.

Version 1:

Reviewer comments:

Reviewer #1

(Remarks to the Author)
I have re-reviewed the manuscript and find that all the comments have been well-addressed. I have no further comments and think it can be accepted as is.

Reviewer #2

(Remarks to the Author)
I am satisfied with the responses and changes made. I think additional refinement of the title is needed to be more precise: which specific bimetallic nanoparticles? otherwise the title is vague

Reviewer #3

(Remarks to the Author)
I co-reviewed this manuscript with one of the reviewers who provided the listed reports. This is part of the Nature Communications initiative to facilitate training in peer review and to provide appropriate recognition for Early Career Researchers who co-review manuscripts.

Open Access This Peer Review File is licensed under a Creative Commons Attribution 4.0 International License, which permits use, sharing, adaptation, distribution and reproduction in any medium or format, as long as you give appropriate credit to the original author(s) and the source, provide a link to the Creative Commons license, and indicate if changes were made.
In cases where reviewers are anonymous, credit should be given to 'Anonymous Referee' and the source.
The images or other third party material in this Peer Review File are included in the article's Creative Commons license, unless indicated otherwise in a credit line to the material. If material is not included in the article's Creative Commons license and your intended use is not permitted by statutory regulation or exceeds the permitted use, you will need to obtain permission directly from the copyright holder.
To view a copy of this license, visit https://creativecommons.org/licenses/by/4.0/

Ref: NCOMMS-25-47822
Title: Shapeshifting bimetallic nanoparticles with switching catalytic performance
Journal: Nature Communications

Responses to the Reviewer's Comments

All authors are pleased to submit the revised version of our manuscript. We appreciate the reviewers for their careful consideration of our manuscript and favorable view of the significance of our findings. We endeavored carefully to address all of the constructive comments and suggestions by the reviewers, all of which have been very useful to improve the clarity of the manuscript.

Our responses to the reviewers' comments are given below. Also attached are the revised manuscript and supplementary information, where revised parts are highlighted in red font.

Reviewer #1

Herein a reversible transformation between Fe_3Ni alloy nanoparticles and $Fe_3Ni@FeO_x$ core–shell structures was cleverly designed for the CO_2-mediated ethane conversion. The results are interesting, and the characterization of the structure is adequate. This is of great significance for the promotion and application of smart catalysts based on perovskite structure. However, as the structural evolution of perovskite-based materials in reducing and oxidizing atmospheres has been reported, so the author should provide advanced insights in evolution of material structure and the effect of exsolution process on catalytic performances. There are two key issues: (1) The reason for the formation of shells on the alloy surface has not been explained clearly? The author only briefly discussed without providing sufficient experimental evidence; (2) There is a difference in activity between the two catalysts, and no further experimental evidence has been provided, mainly relying on the relationship between the structure and activity proposed in the reported literatures. Therefore, I recommend that this paper should be resubmitted after revision. The other comments are as follows:

We appreciate reviewer #1 for acknowledging the academic significance and technical impact of our research.

Thank you very much for bringing issue (1) to our attention. We acknowledge that presenting two different formation processes for the FeO_x shell could be misleading to readers if not supported by precise experimental evidence. A comprehensive understanding of the oxide shell formation would require multiple, complex in situ experiments (e.g., XPS, DRIFTS), which are beyond the scope of the present study. To simplify the discussion, we therefore focus on the strong metal-support interaction (SMSI) mechanism, which is considerably better studied than the reverse oxygen spillover effect.

In this way, the selective formation of a FeO_x layer from the $La_{0.4}Sr_{0.4}Ti_{0.6}Fe_{0.35}Ni_{0.05}O_{3-\delta}$ solid solution can be rationalized by considering the Tammann temperatures for the binary oxides forming the perovskite structure - i.e., the temperature at which ions in the bulk of a solid become thermally mobile and can diffuse more readily, approximately half of the material's absolute melting temperature. Neglecting NiO due to its low content, FeO and Fe_3O_4 have Tammann temperatures of roughly 560 °C and 600 °C, respectively, whereas TiO_2, SrO, and La_2O_3 exhibit much higher values, exceeding 800 °C. These estimates are in excellent agreement with our experimental observations. Based on extensive ex situ characterization—including XANES, Mössbauer spectroscopy, XRD, and TEM of reduced samples at different temperatures—supplemented by in situ TEM, it is observed that the formation of the iron oxide shell is temperature-activated, initiating at approximately 600 °C. In situ TEM and Mössbauer spectroscopy further reveal that the shell's thickness and atomic ordering increase with temperature up to 850 °C. Mössbauer data indicate that its atomic composition resembles that of wüstite, whereas TEM imaging reveals magnetite domains. As both phases coexist in the Fe/O phase diagram [1], it is reasonable to assume that the shell generally consists of a defective FeO_x layer with Fe(II/III) in a cubic crystal habit.

This new discussion has been added at page 8 of the manuscript.

Regarding issue (2), we kindly note that we have proceeded to greatly expand the catalytic evaluation part and the corresponding discussion. New catalytic experiments have been performed, additional key performance metrics (i.e., reaction rates, activation energy values etc.) have been calculated, comparison of catalytic performance at similar C_2H_6 conversion levels has been made, and also a multi-cyclic catalytic experiment incorporating in-situ

regeneration (oxidation/ reduction) treatments has been performed. Moreover, additional post-reaction characterization on the spent materials has been carried out as follow: (1) the discussion on the potential reaction mechanism has been revised/ improved, based on the relevant recent literature. ***Figure 7*** *has also been revised accordingly. (2) a relevant discussion has been included based on the new temperature step experiments with 10 °C temperature step (see also* **Supplementary Fig. 11, 12, and Supplementary Table 3 and 4***). In short, based on these new experiments, the activation energy values (for C_2H_6 and CO_2 conversion, as well as for CO and C_2H_4 production) were calculated and the results were discussed. Moreover, a comparison of the intrinsic catalytic activity is made at similar C_2H_6 conversion levels for the four different materials. (3) a relevant discussion has been included based on a new multi-cyclic catalytic experiment with intermediate in-situ regeneration (oxidation/ reduction) treatments (****Figure 9****). It is shown, that the regeneration treatments (previously done ex-situ) can also proceed in-situ during catalytic operation. In short, following oxidation and reduction at 850 °C, the catalytic performance and reaction selectivity can be almost entirely switched from reforming to dehydrogenation with great success. During subsequent oxidation at 850 °C and reduction at 400 °C, the catalytic performance and selectivity switch was partial (similar performance also observed for R850-O-R400 in* ***Figure 7****). As such future work needs directed in the complete perovskite nanostructure regeneration and thus to full selectivity switching under multi-cyclic operation. For these changes, please refer to the aforementioned parts of the revised manuscript and revised supplementary information.*

References

[1]: Darken, L.S.; Gurry, R.W. "The system Iron-Oxygen:1.The Wüstite field and related euilibria J. Am. Chem. Soc. 1945, 67, 8, 1398–1412

Comment 1 of Reviewer 1: The formation of a quite porous morphology is detected by transmission electron microscopy analysis. This is not enough, and the pore structure should be evaluated by nitrogen adsorption-desorption.

We thank the reviewer for this valuable suggestion. In response, we further characterized the materials by N_2 physisorption measurements. For all samples, the collected adsorption–desorption isotherms correspond to Type II behavior, which is characteristic of macroporous or non-porous materials (see Figure R1). As these isotherms do not exhibit a

hysteresis loop, the mesopore size distribution cannot be reliably determined; therefore, the only meaningful parameter that can be extracted is the specific surface area, calculated using the BET model.

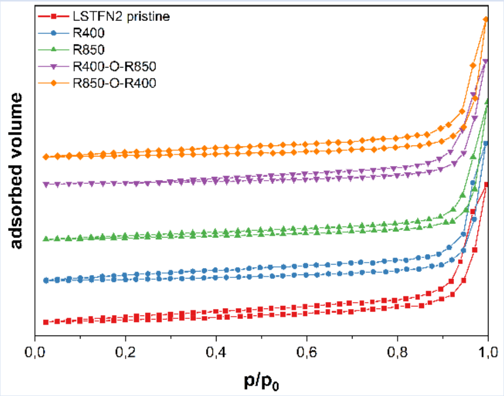

Figure R1. *Nitrogen physisorption isotherms of the pristine and exsolved materials*

Analysis of the BET surface areas (see ***Table R1****) shows that, after the first exsolution treatment, only a non-significant decrease in specific surface area is observed. More importantly, despite the different exsolution conditions and the substantial structural modifications induced by exsolution, the overall porosity of the materials remains largely unaffected. A noticeable decrease in specific surface area is observed only for the shapeshifted materials, which can be attributed to the prolonged high-temperature oxidation followed by the re-reduction process.*

Table R1: *BET specific surface area values of the pristine and exsolved materials*

Sample	*BET specific surface area [m^2g^{-1}]*
LSTFN2 (pristine)	*15*
R400	*13*
R850	*14*
R850OR400	*10*
R400OR850	*10*

We added the discussion of the SSA at page 2 and 4 for the pristine, exsolved and at shapeshifted materials. The BET values have been added in Table 1 and the physisorp tion isotherms integrated in ***Supplementary Fig. 1***

Comment 2 of Reviewer 1: The bar of TEM and EELS spectroscopy in Figure 3 is not clear. Please modify. The images of Figure 3C should be rotated to intuitively display the thickness of thin amorphous shell. The description of Figure 3e is absent. Please add the information.

We thank for the reviewer's comment. To clearly suggest the scale of each TEM images and the thickness of shell layer, we revise the ***Figure 3*** *as below. We also added description for EELS data for R850 sample, as well. The revised version is presented in page 5 of manuscript.*

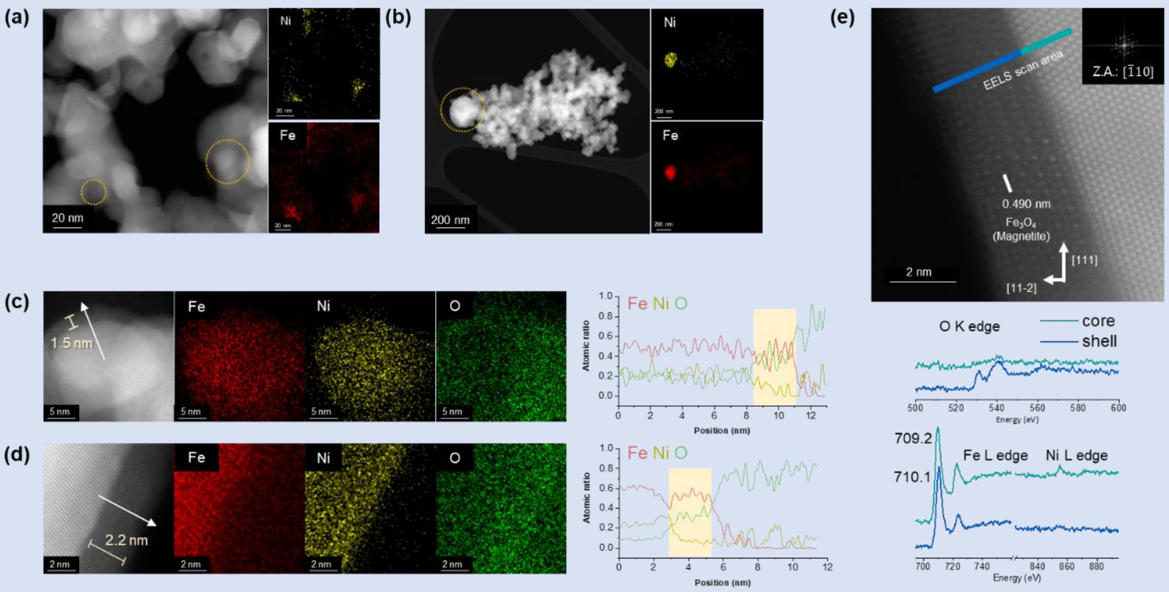

Figure R2*. HADDF-STEM image and respective iron (red) and nickel (yellow) EDX mapping result of the R400 (a), and R850 (b) materials; High-resolution images and EDX mapping result with line-scan signal (scan direction and shell thickness are denoted in the figure) of R400 (c) and R850 (d) materials; (e) Atomic resolution STEM and EELS result of R850, indicating the different composition of the core and the shell portions in the exsolved nanoparticles.*

Comment 3 of Reviewer 1: In situ TEM experiments were performed under high-vacuum conditions to avoid the disturbing effect of the air exposure (Figure 4). In other word, H2 was absent, so that what's the role of H2 atmosphere during the exsolution process?

We sincerely appreciate the reviewer's valuable comment. Exsolution, a spontaneous phase separation process in a multicomponent solid solution, based on the differences in reduction tendency of each ionic element. Once the oxygen chemical potential gradient is formed across the solid-gas interface, it drives flux of point defect within the solid matrix to equilibrate the potential that accompanies oxygen vacancy and metal precipitate [2]. Such reaction can be expressed as below:

$$\mathrm{M}_{\mathrm{M}}^{\times} + \frac{m}{2} O_{O}^{\times} = V_{M}^{m'} + \frac{m}{2}\ V_{\mathrm{O}}^{\cdot\cdot} + M_{metal,surf} + \frac{m}{4}\mathrm{O}_{2,(\mathrm{g})}$$

From a view of defect chemistry, exsolution involves the release of oxygen ions as a gas, leaving a positive oxygen vacancy and reduced metal cations in a form of metallic state. These reduction processes occur when ΔG for a total reaction is negative, suggesting that hydrogen is not an essential factor to trigger exsolution. However, hydrogen can be the simplest agent to create differences in oxygen chemical potential at relatively mild conditions. ***Table R2*** *summarizes ΔG for the reduction process of various binary metal oxides. Once the hydrogen is introduced in the reduction process, it significantly lowers the value, thereby turns the equilibrium towards forward direction. Thus, hydrogen can shift the overall reaction forward, thereby accelerating the exsolution process. In our in-situ experiment, the vacuum level reaches near 10^{-9} mbar, which is far lower than the typical atmosphere (pO_2 = 210 mbar). Also, there is a minute amount of hydrogen that inevitably comes from the metal parts of the TEM apparatus and the vacuum facility as well [3]. In sum, we would like to say that hydrogen stimulates the exsolution process, and our in situ condition is far enough to trigger the initial growth behavior of exsolution as like reported in earlier literatures [4].*

Table R2. *Comparison of ΔG_{red} for various binary metal oxide at 900 ℃ condition. ΔG_{red} represent Gibbs free energy changes for reduction process. We assume two reaction equation; $2MO = 2M + O_{2(g)}$, $MO + H_2 = M + H_2O_{(g)}$, for w/o H_2 and w/ H_2, respectively.*

Element	ΔG_{red} w/o H_2 (kJ/mol)	ΔG_{red} w/ H_2 (kJ/mol)

NiO	*267.2*	***-49.5***
CoO	*289.1*	***-31.6***
MnO	*596.9*	***115.3***
FeO	*375.3*	***4.5***
PdO	*-17.5*	***-191.9***

References

[2] S. Yanagisawa et al., J. Phys. Chem. C, 2013, 117 (3), 1278-1286.

[3] N. Miyauchi et al., J. Vac. Sci. Technol., 2024, 42 (1), 013201.

[4] D. Neagu et al., ACS Nano, 2019, 13 (11), 12996-13005.

Comment 4 of Reviewer 1: It was stated that EDXS results of the in situ TEM analyses show that the shell layer is composed of iron and oxygen only (Figure S4). Figure S4 should be corrected to Figure S3. Please check it. On the other hand, accord to the EDXS results, the conclusion that iron and oxygen are enriched on the surface of particles is very inconclusive. Please provide more favorable EDXS evidence.

*We thank the reviewer for the comment on the EDX data. We corrected the number of supplementary figures. Regards to the EDX result during in situ TEM analysis, there are still undeveloped technical issues in acquiring X-ray signals at such high temperature (900 ℃). We eliminate the drifts by using a piezoelectric stage and Velox software; however, incident electrons and emitted X-ray scatters by intense infrared which increase the noise level. Even with the integrated wide-angle detector in the Ultra-X EDX system (Thermo Fischer Scientific), it is a challenge to eliminate the noise level during the high-temperature EDX analysis. Thus, we conducted an extra EDX experiment and collected additional data on it (**Figure R3**). Also, we've measured the d-spacing of the shell layer (~ 1.7 Å), which implies the shell is likely to be Fe_3O_4, not a metallic species. We henceforth revise the*

Supplementary Fig. 4.

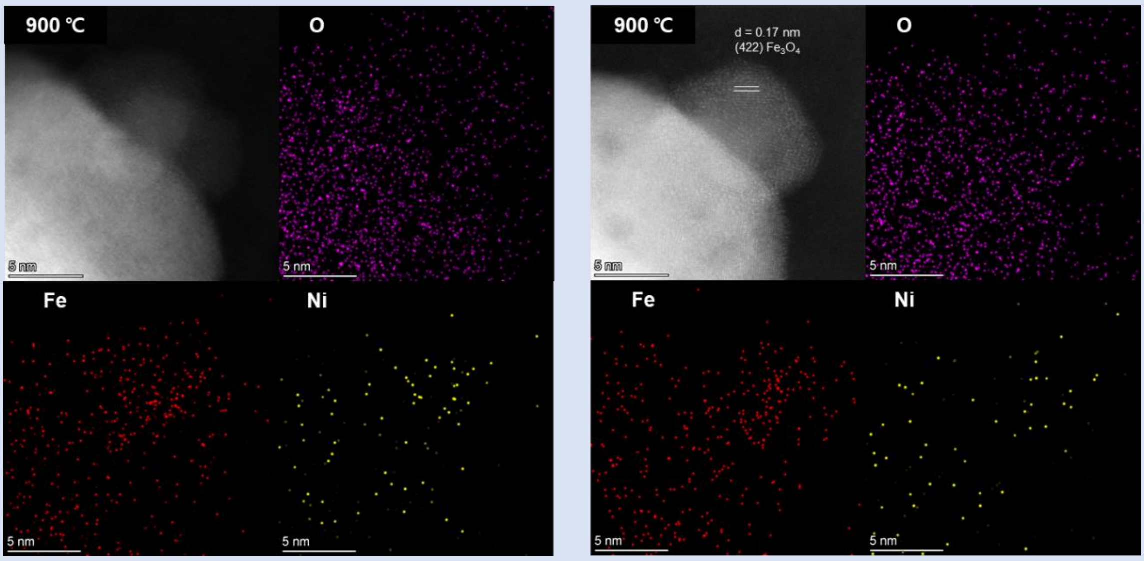

Figure R3. *In situ HADDF-EDX mapping of the LSTFN2 material at 900 ℃.*

Comment 5 of Reviewer 1: Two Figure 4 are in present manuscript. The numbering of the image is incorrect.

We thank for the reviewer's kind attention to details. We hence revise the number of Figure in the manuscript.

Comment 6 of Reviewer 1: The author concluded that exsolved nanoparticles in R400 (Figure 3d) are covered with thin amorphous shell (~ 1 nm) of iron oxide (FeOx). However, in Figure 4c, the shell was absent in the picture of the shapeshifting mechanism. Why?

We appreciate the reviewer's comment. Figure 4c still presents FeO_x outmost shell, which is formed by the air exposure prior to the TEM analysis. However, the displayed images do not clearly reveal the amorphous layer due to the contrast in the image. Thereafter, we've conducted overlay image mapping process and line profile including HADDF intensity to

*figure out the presence of FeO_x shell for ROR400 sample (**Figure R4**). As can be seen in the element distribution along the line scan area, there are distinct FeO_x-domains (~ 1 nm of thickness) which underpin the shell structure for ROR400. We've added it as **Supplementary Fig. 8** to prevent misleading the readers. Please refer to the figures as below:*

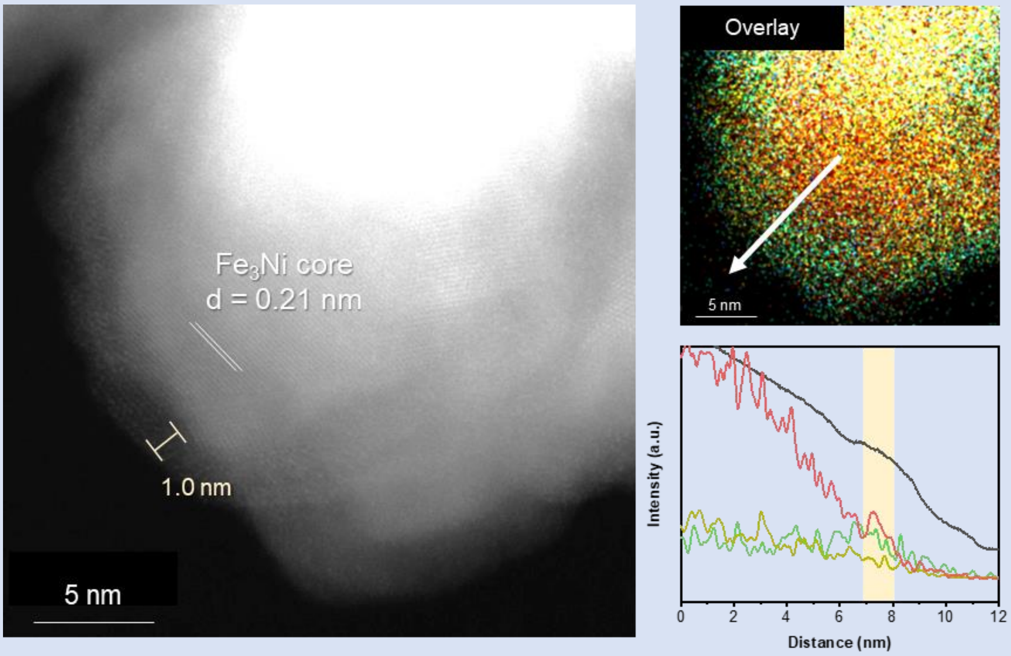

***Figure R4.** HADDF-STEM EDX analysis result for ROR400 and corresponding line profile of each element across the boundary area of the core-shell structure. The colored area represents the amorphous FeO_x shell domains, surrounding the alloy core.*

Comment 7 of Reviewer 1: What's the ideal product and reaction routine for CO2-mediated ethane conversion? The activity of the R400 and R850 catalysts should be compared with the literature results, to highlight the novelty of exsolution process for designing multifunctional catalyst materials.

We thank the Reviewer for his/her valuable comment. The reaction between ethane and CO_2 can mainly lead to two different pathways, the CO_2-assisted oxidative dehydrogenation of ethane (1), and the dry reforming of ethane (2):

$$C_2H_6 + CO_2 \rightarrow C_2H_4 + CO + H_2O \text{ (ODH)} \quad (1)$$

$$C_2H_6 + 2CO_2 \rightarrow 4CO + 3H_2 \text{ (DER)} \quad (2)$$

Among them, the first reaction (1) – ODH, is undoubtedly more desired, since the produced C_2H_4 is a more valuable product than H_2/CO (syngas). This reaction also proposes a potential alternative to the current industrial production of ethylene (C_2H_4) via steam cracking. In the case of reaction (1), CO_2 is used as a reactant (specifically as a soft oxidant), and the reaction can also be operated at a lower temperature than steam cracking or the non-oxidative dehydrogenation, which can significantly reduce the carbon footprint of ethylene production, given the rapidly growing ethylene market [5].

Reaction (2) – DER, is less desired than ODH, but it can still be valuable, considering that underutilized ethylene (e.g., from shale gas) reacts with captured CO_2 in order to produce valuable synthesis gas (H_2 and CO mixture), which can be used in subsequent Fischer-Tropsch synthesis. Furthermore, the dry reforming of ethane can be performed at milder conditions (roughly 100 ℃ lower temperature) compared to the typical reaction of dry reforming of methane [6].

References

[5] A.N. Biswas, et al., Joule, 2022, 6(2), 269–273.

[6] T. Zhang, et al., Engineering, 2022, 18, 173–185.

Until now, many different catalyst materials have been prepared in order to achieve high catalytic activity, selectivity, and stability, but only for their respective targeted reaction (i.e., CO_2-assisted dehydrogenation of ethane OR dry ethane reforming). Therefore, the novelty of the current work does not specifically lie in overcoming these reaction metrics compared to other reported catalysts, but rather in demonstrating the potential to switch the reaction selectivity (between reactions (1) and (2)) by simply changing the exsolution treatment conditions on a single multifunctional perovskite catalyst material and also via potential regeneration treatments during multi-cyclic operation. As such, the different material structures reported in this work are derived from a single perovskite material (with $La_{0.4}Sr_{0.40}Ti_{0.6}Fe_{0.35}Ni_{0.05}O_{3\pm\delta}$ formula) by just changing the exsolution conditions (specifically temperature), which subsequently leads to a different surface chemistry/ presence of different surface-active sites, and thus different reaction selectivity. This is the first time that this has been reported for this particular reaction, as other works typically

rely on changing the material composition in order to achieve variable reaction selectivity (e.g., via the variation of the Fe:Ni molar ratio). The fact that the catalytic performance/ selectivity is (at least partially) switchable following re-oxidation and re-exsolution on the same material composition is an added advantage to this approach, but further future work needs to directed toward complete perovskite nanostructure regeneration and thus full selectivity switching under multi-cyclic operation.

*Subsequently, as per the Reviewer's suggestion, we proceeded to provide a comparison Table in the supplementary information (**Supplementary Table 2**), where the catalytic performance of R400 and R850 (different exsolution treatment) is compared to other catalytic materials reported in the literature, for the respective reactions (CO_2-assisted oxidative ethane dehydrogenation and dry ethane reforming). It is noted that the majority of the relevant literature works perform this reaction at a lower temperature (600 ℃). Nevertheless, R850 does compare quite favorably for the dehydrogenation reaction (ODH) even when compared to works using similar reaction temperature (650 – 700 ℃). R400 provides a decent dry reforming reactivity, but it can be outcompeted by other catalysts in the literature operating at a similarly high temperature, providing that they also show high stability with minimal coke deposition. In any case, the ability to switch the catalytic selectivity on the same material by simply altering the exsolution treatment is a unique advantage of the current work, and this in turn highlights the novelty of the exsolution process for designing multifunctional catalyst materials. The comparison Table is also presented below for convenience:*

Table R3. *Catalytic performance comparison between R400, R850, and other reported catalysts in the literature for the respective reactions of CO_2-assisted oxidative dehydrogenation of ethane (ODH) and dry ethane reforming (DER).*

**1) CO_2-assited oxidative dehydrogenation of ethane (ODH)**			
**Catalyst**	***Reaction conditions***	***Catalytic Performance***	***Ref.***
R850 ($La_{0.4}Sr_{0.40}Ti_{0.6}Fe_{0.35}Ni_{0.05}O_{3\pm\delta}$ with exsolution at 850 ℃)	*WHSV = 24000 ml g_{cat}^{-1} h^{-1} C_2H_6:CO_2 = 1:1 T = 700 ℃ (8 h)*	$X_{C_2H_6}$ = 22.1% X_{CO_2} = 30.2% $S_{C_2H_4}$ = 75.2%	*This work*
Ni_1Fe_3/CeO_2	*WHSV = 24000 ml*	$X_{C_2H_6}$ = 3.5%	*[7]*

	g_{cat}^{-1} h^{-1}<br>$C_2H_6:CO_2$ = 1:1<br>T = 600 ℃ (11-13 h)	X_{CO_2} = 5.9%<br>$S_{C_2H_4}$ = 77.5%	
1.0% PdFe (1:9)/ CeO_2	WHSV = 24000 ml g_{cat}^{-1} h^{-1}<br>$C_2H_6:CO_2$ = 1:1<br>T = 600 ℃ (11-12 h)	$X_{C_2H_6}$ = 6.6%<br>X_{CO_2} = 8.3%<br>$S_{C_2H_4}$ = 85.8%	[8]
NiFe/CeO_2-C1<br>(Ni:Fe = 1:4)	WHSV = 24000 ml g_{cat}^{-1} h^{-1}<br>$C_2H_6:CO_2$ = 1:1<br>T = 600 ℃ (12 h)	$X_{C_2H_6}$ = 4.6%<br>X_{CO_2} = 6.2%<br>$S_{C_2H_4}$ = 83%	[9]
$Fe_{1.5}Ni_{0.5}/ZrO_2$-M<br>(ZrO_2 monoclinic)	WHSV = 9000 ml g_{cat}^{-1} h^{-1}<br>$C_2H_6:CO_2$ = 1:1<br>T = 700 ℃ (100 min)	$X_{C_2H_6}$ = 23.4%<br>X_{CO_2} = 28.0%<br>$S_{C_2H_4}$ = 80%	[10]
$Ti_{0.5}Ce_{0.5}O_2$	WHSV = 6000 ml g_{cat}^{-1} h^{-1}<br>$C_2H_6:CO_2$ = 1:1<br>T = 700 ℃ (8 h)	$X_{C_2H_6}$ = 32.3%<br>X_{CO_2} = 34.6%<br>$S_{C_2H_4}$ = 54.2%	[11]
Fe/NiO-MgO-ZrO_2<br>(5Fe/10NiMgZr, Fe-700 (5h))	WHSV = 7500 ml g_{cat}^{-1} h^{-1}<br>$C_2H_6:CO_2$ = 1:1<br>T = 650 ℃ (6.5 h)	$X_{C_2H_6}$ = 23%<br>X_{CO_2} = 25%<br>$S_{C_2H_4}$ = 90%	[12]
Fe/$MgAl_2O_4$<br>(Fe/3Mg7Al)	WHSV = 6000 ml g_{cat}^{-1} h^{-1}<br>$C_2H_6:CO_2$ = 1:1<br>T = 650 ℃ (60 min)	$X_{C_2H_6}$ = 21%<br>X_{CO_2} = 20%<br>$S_{C_2H_4}$ = 84%	[13]
$Fe_{1.5}Ni_{0.5}/CeO_2$	WHSV = 9000 ml g_{cat}^{-1} h^{-1}<br>$C_2H_6:CO_2$ = 1:1<br>T = 700 ℃ (2 h)	$X_{C_2H_6}$ = 20%<br>X_{CO_2} = 16%<br>$S_{C_2H_4}$ = 65%	[14]
$Fe_{1.5}Ni_{0.5}$/CeTi-C-2<br>(2% Ti substitution)	WHSV = 9000 ml g_{cat}^{-1} h^{-1}<br>$C_2H_6:CO_2$ = 1:1<br>T = 650 ℃ (1 h)	$X_{C_2H_6}$ = 10.6%<br>X_{CO_2} = 16.3%<br>$S_{C_2H_4}$ = 82.7%	[15]
$MgFeAlO_4$	WHSV = 9000 ml g_{cat}^{-1} h^{-1}<br>$C_2H_6:CO_2$ = 1:5<br>T = 600 ℃ (8 h)	$X_{C_2H_6}$ = 25.1%<br>X_{CO_2} = 22.1%<br>$S_{C_2H_4}$ = 92.8%	[16]
**2) Dry ethane reforming (DER)**			
**Catalyst**	**Reaction conditions**	**Catalytic Performance**	**Ref.**
R400<br>($La_{0.4}Sr_{0.40}Ti_{0.6}Fe_{0.35}Ni_{0.05}O_{3\pm\delta}$ with exsolution at 400 ℃)	WHSV = 24000 ml g_{cat}^{-1} h^{-1}<br>$C_2H_6:CO_2$ = 1:1<br>T = 700 ℃ (8 h)	$X_{C_2H_6}$ = 32.6%<br>X_{CO_2} = 67.6%<br>S_{CO} = 78.3%	This work
Ni_3Fe_1/CeO_2	WHSV = 24000 ml	$X_{C_2H_6}$ = 16.7%	[7]

	g_{cat}^{-1} h^{-1}<br>$C_2H_6:CO_2 = 1:1$<br>T = 600 ℃ (11-13 h)	X_{CO_2} = 41.3%<br>S_{CO} = 98.6%	
$Ni_{0.05}Ce_{0.975}O_2$	WHSV = 9000 ml g_{cat}^{-1} h^{-1}<br>$C_2H_6:CO_2 = 1:1$<br>T = 700 ℃ (2 h)	$X_{C_2H_6}$ = 63.9%<br>X_{CO_2} = 88.1%<br>S_{CO} = 94.3%	[11]
$LaFe_{0.9}Ni_{0.1}O_3$	WHSV = 24000 ml g_{cat}^{-1} h^{-1}<br>$C_2H_6:CO_2 = 1:1$<br>T = 600 ℃ (12-14 h)	$X_{C_2H_6}$ = 21.4%<br>X_{CO_2} = 49.7%<br>S_{CO} = 97.5%	[17]
$PtNi/CeO_2$ (Pt:Ni = 1:3)	WHSV = 24000 ml g_{cat}^{-1} h^{-1}<br>$C_2H_6:CO_2 = 1:1$<br>T = 600 ℃ (12 h)	$X_{C_2H_6}$ = 22.8%<br>X_{CO_2} = 53.4%<br>S_{CO} = 92.8%	[18]
$Ni/MgAl_2O_4$	WHSV = 60000 ml g_{cat}^{-1} h^{-1}<br>$C_2H_6:CO_2 = 1:2$<br>T = 800 ℃ (18 h)	$X_{C_2H_6}$ = 96%<br>X_{CO_2} = 91%<br>S_{CO} = 93%	[19]
$CaZr_{0.8}Ni_{0.2}O_{3-\delta}$	WHSV = 15000 ml g_{cat}^{-1} h^{-1}<br>$C_2H_6:CO_2 = 1:2$<br>T = 700 ℃ (8 h)	$X_{C_2H_6}$ = 83%<br>X_{CO_2} = 82%<br>S_{CO} = 93%	[20]
$FeNi/Ce\text{-}Al_{0.5}$ (Fe:Ni = 3:1)	WHSV = 24000 ml g_{cat}^{-1} h^{-1}<br>$C_2H_6:CO_2 = 1:1$<br>T = 600 ℃ (8 h)	$X_{C_2H_6}$ = 11.7%<br>X_{CO_2} = 33.1%<br>S_{CO} = 97.7%	[21]
$NdFe_{0.7}Ni_{0.3}O_3$	WHSV = 60000 ml g_{cat}^{-1} h^{-1}<br>$C_2H_6:CO_2 = 1:2$<br>T = 600 ℃ (8-10 h)	$X_{C_2H_6}$ = 31.5%<br>X_{CO_2} = 42.4%<br>S_{CO} = 97.8%	[22]
Ni-2.4La@Si	WHSV = 36000 ml g_{cat}^{-1} h^{-1}<br>$C_2H_6:CO_2 = 1:2$<br>T = 700 ℃ (200 h)	$X_{C_2H_6}$ = 96%<br>X_{CO_2} = 86%<br>S_{CO} = 87%	[23]
Ni-Si@Ce	WHSV = 24000 ml g_{cat}^{-1} h^{-1}<br>$C_2H_6:CO_2 = 1:2$<br>T = 650 ℃ (10 h)	$X_{C_2H_6}$ = 70%<br>X_{CO_2} = 71%<br>S_{CO} = 97%	[24]

References

[7] B. Yan, et al., Proc. Natl. Acad. Sci., 2018, 115(12), 2902-2907.

[8] Z. Xie, et al., Chem, 2020, 6(10), 2703–2716.

[9] M. Guo, et al., ChemCatChem, 2021, 13(13), 3119-3131.

[10] X. Li, et al., Fuel, 2022, 322, 124122.

[11] L. Li, et al., Appl. Catal. A: Gen., 2022, 635, 118565.

[12] M. Tasioula, et al., ACS Catal., 2023, 13(4), 2176–2189.

[13] Y. Zheng, et al., ACS Catal., 2023, 14(7), 11153−11163.

[14] X. Li, et al., Int. J. Hydrogen Energy., 2024, 79, 45–59.

[15] X. Li, et al., Chem. Eng. J., 2024, 484, 149047.

[16] Q. Du, et al., Carbon Capture Sci. Technol., 2025, 14, 100327.

[17] B. Zhao, et al., J. Catal., 2018, 358, 168–178.

[18] Z. Xie, et al., Appl. Catal. B: Environ., 2019, 245, 376–388.

[19] K. H. Kim, et al., J. CO2 Util., 2021, 53, 101713.

[20] A. I. Tsiotsias, et al., J. CO2 Util., 2022, 61, 102046.

[21] T. Zhang, et al., Engineering., 2022, 18, 173–185.

[22] Y. Wang, et al., Appl. Catal. B: Environ., 2023, 337, 123010.

[23] C. Wang, et al., Chem. Eng. J., 2024, 491, 152091.

[24] X. Chen, et al., Int. J. Hydrogen Energy., 2025, 103, 501–512.

Please note, that relevant changes have been made in the revised manuscript, and supplementary information file with red font.

Reviewer #2

The authors investigate dynamic redox behavior in layered double hydroxide (LDH)-derived NiFe catalysts for ethylene and CO_2 hydrogenation, focusing on the interplay between structural evolution and catalytic performance. Through a combination of operando XAS, in situ STEM, and transient measurements, they propose that redox cycles during activation and reaction influence selectivity and conversion. The topic aligns well with the scope of Nature Communications. However, the core claims remain unconvincing due to oversimplification in the interpretation of catalytic performance. While the work touches on important themes in catalyst restructuring and redox chemistry, the manuscript does not provide a comprehensive discussion of prior studies in LDH-derived systems or other dynamic metal-oxide catalysts in CO_2/ethylene hydrogenation. Several relevant works are overlooked, which limits the ability to understand the novelty and implications of the current findings. A stronger foundation in the literature would help frame the study's contributions and gaps more clearly.

We thank the Reviewer for his/her valuable comment. ***We kindly note, that the materials used in this work are perovskite-derived, not LDH-based materials. They consist of a perovskite backbone structure with supported exsolved bimetallic Fe-Ni nanostructures, that differ based on the exsolution treatment conditions and the regeneration treatments.*** *Furthermore, the catalytic testing that we performed utilized ethane and CO_2 as co-reactants. The two main reactions occurring are CO_2-assisted oxidative dehydrogenation of ethane (reaction (1)), and dry ethane reforming (reaction (2)), which are shown below:*

$$C_2H_6 + CO_2 \rightarrow C_2H_4 + CO + H_2O \text{ (ODH)} \quad (1)$$

$$C_2H_6 + 2CO_2 \rightarrow 4CO + 3H_2 \text{ (DER)} \quad (2)$$

*In any case, in agreement with the Reviewer's suggestion, we proceeded to expand the discussion on relevant literature works, as a means to establish a stronger foundation with the relevant literature and highlight the novelty and contributions of the current study (**Table R1**).*

Table R1. *Catalytic performance comparison between R400, R850, and other reported catalysts in the literature for the respective reactions of CO_2-assisted oxidative dehydrogenation of ethane (ODH) and dry ethane reforming (DER).*

**1) CO_2-assited oxidative dehydrogenation of ethane (ODH)**			
**Catalyst**	***Reaction conditions***	***Catalytic Performance***	***Ref.***
R850 ($La_{0.4}Sr_{0.40}Ti_{0.6}Fe_{0.35}Ni_{0.05}O_{3\pm\delta}$ with exsolution at 850 ℃)	*WHSV = 24000 ml g_{cat}^{-1} h^{-1} $C_2H_6:CO_2$ = 1:1 T = 700 ℃ (8 h)*	$X_{C_2H_6}$ = 22.1% X_{CO_2} = 30.2% $S_{C_2H_4}$ = 75.2%	*This work*
Ni_1Fe_3/CeO_2	*WHSV = 24000 ml g_{cat}^{-1} h^{-1} $C_2H_6:CO_2$ = 1:1 T = 600 ℃ (11-13 h)*	$X_{C_2H_6}$ = 3.5% X_{CO_2} = 5.9% $S_{C_2H_4}$ = 77.5%	*[1]*
1.0% PdFe (1:9)/ CeO_2	*WHSV = 24000 ml g_{cat}^{-1} h^{-1} $C_2H_6:CO_2$ = 1:1 T = 600 ℃ (11-12 h)*	$X_{C_2H_6}$ = 6.6% X_{CO_2} = 8.3% $S_{C_2H_4}$ = 85.8%	*[2]*
NiFe/CeO_2-C1 (Ni:Fe = 1:4)	*WHSV = 24000 ml g_{cat}^{-1} h^{-1} $C_2H_6:CO_2$ = 1:1 T = 600 ℃ (12 h)*	$X_{C_2H_6}$ = 4.6% X_{CO_2} = 6.2% $S_{C_2H_4}$ = 83%	*[3]*
$Fe_{1.5}Ni_{0.5}/ZrO_2$-M (ZrO_2 monoclinic)	*WHSV = 9000 ml g_{cat}^{-1} h^{-1} $C_2H_6:CO_2$ = 1:1 T = 700 ℃ (100 min)*	$X_{C_2H_6}$ = 23.4% X_{CO_2} = 28.0% $S_{C_2H_4}$ = 80%	*[4]*
$Ti_{0.5}Ce_{0.5}O_2$	*WHSV = 6000 ml g_{cat}^{-1} h^{-1} $C_2H_6:CO_2$ = 1:1 T = 700 ℃ (8 h)*	$X_{C_2H_6}$ = 32.3% X_{CO_2} = 34.6% $S_{C_2H_4}$ = 54.2%	*[5]*
Fe/NiO-MgO-ZrO_2 (5Fe/10NiMgZr, Fe-700 (5h))	*WHSV = 7500 ml g_{cat}^{-1} h^{-1} $C_2H_6:CO_2$ = 1:1 T = 650 ℃ (6.5 h)*	$X_{C_2H_6}$ = 23% X_{CO_2} = 25% $S_{C_2H_4}$ = 90%	*[6]*
Fe/$MgAl_2O_4$ (Fe/3Mg7Al)	*WHSV = 6000 ml g_{cat}^{-1} h^{-1} $C_2H_6:CO_2$ = 1:1 T = 650 ℃ (60 min)*	$X_{C_2H_6}$ = 21% X_{CO_2} = 20% $S_{C_2H_4}$ = 84%	*[6]*
$Fe_{1.5}Ni_{0.5}/CeO_2$	*WHSV = 9000 ml g_{cat}^{-1} h^{-1} $C_2H_6:CO_2$ = 1:1 T = 700 ℃ (2 h)*	$X_{C_2H_6}$ = 20% X_{CO_2} = 16% $S_{C_2H_4}$ = 65%	*[7]*
$Fe_{1.5}Ni_{0.5}$/CeTi-C-2 (2% Ti substitution)	*WHSV = 9000 ml g_{cat}^{-1} h^{-1} $C_2H_6:CO_2$ = 1:1*	$X_{C_2H_6}$ = 10.6% X_{CO_2} = 16.3% $S_{C_2H_4}$ = 82.7%	*[8]*

	T = 650 °C (1 h)		
$MgFeAlO_4$	*WHSV = 9000 ml g_{cat}^{-1} h^{-1} C_2H_6:CO_2 = 1:5 T = 600 °C (8 h)*	$X_{C_2H_6}$ = 25.1% X_{CO_2} = 22.1% $S_{C_2H_4}$ = 92.8%	*[9]*
	2) Dry ethane reforming (DER)		
Catalyst	***Reaction conditions***	***Catalytic Performance***	***Ref.***
R400 ($La_{0.4}Sr_{0.40}Ti_{0.6}Fe_{0.35}Ni_{0.05}O_{3\pm\delta}$ with exsolution at 400 °C)	*WHSV = 24000 ml g_{cat}^{-1} h^{-1} C_2H_6:CO_2 = 1:1 T = 700 °C (8 h)*	$X_{C_2H_6}$ = 32.6% X_{CO_2} = 67.6% S_{CO} = 78.3%	*This work*
$Ni_{0.05}Ce_{0.975}O_2$	*WHSV = 9000 ml g_{cat}^{-1} h^{-1} C_2H_6:CO_2 = 1:1 T = 700 °C (2 h)*	$X_{C_2H_6}$ = 63.9% X_{CO_2} = 88.1% S_{CO} = 94.3%	*[5]*
Ni_3Fe_1/CeO_2	*WHSV = 24000 ml g_{cat}^{-1} h^{-1} C_2H_6:CO_2 = 1:1 T = 600 °C (11-13 h)*	$X_{C_2H_6}$ = 16.7% X_{CO_2} = 41.3% S_{CO} = 98.6%	*[10]*
$LaFe_{0.9}Ni_{0.1}O_3$	*WHSV = 24000 ml g_{cat}^{-1} h^{-1} C_2H_6:CO_2 = 1:1 T = 600 °C (12-14 h)*	$X_{C_2H_6}$ = 21.4% X_{CO_2} = 49.7% S_{CO} = 97.5%	*[11]*
$PtNi/CeO_2$ (Pt:Ni = 1:3)	*WHSV = 24000 ml g_{cat}^{-1} h^{-1} C_2H_6:CO_2 = 1:1 T = 600 °C (12 h)*	$X_{C_2H_6}$ = 22.8% X_{CO_2} = 53.4% S_{CO} = 92.8%	*[12]*
$Ni/MgAl_2O_4$	*WHSV = 60000 ml g_{cat}^{-1} h^{-1} C_2H_6:CO_2 = 1:2 T = 800 °C (18 h)*	$X_{C_2H_6}$ = 96% X_{CO_2} = 91% S_{CO} = 93%	*[13]*
$CaZr_{0.8}Ni_{0.2}O_{3-\delta}$	*WHSV = 15000 ml g_{cat}^{-1} h^{-1} C_2H_6:CO_2 = 1:2 T = 700 °C (8 h)*	$X_{C_2H_6}$ = 83% X_{CO_2} = 82% S_{CO} = 93%	*[14]*
$FeNi/Ce\text{-}Al_{0.5}$ (Fe:Ni = 3:1)	*WHSV = 24000 ml g_{cat}^{-1} h^{-1} C_2H_6:CO_2 = 1:1 T = 600 °C (8 h)*	$X_{C_2H_6}$ = 11.7% X_{CO_2} = 33.1% S_{CO} = 97.7%	*[15]*
$NdFe_{0.7}Ni_{0.3}O_3$	*WHSV = 60000 ml g_{cat}^{-1} h^{-1} C_2H_6:CO_2 = 1:2 T = 600 °C (8-10 h)*	$X_{C_2H_6}$ = 31.5% X_{CO_2} = 42.4% S_{CO} = 97.8%	*[16]*
Ni-2.4La@Si	*WHSV = 36000 ml g_{cat}^{-1} h^{-1} C_2H_6:CO_2 = 1:2*	$X_{C_2H_6}$ = 96% X_{CO_2} = 86%	*[17]*

	T = 700 ℃ (200 h)	S_{CO} = 87%	
Ni-Si@Ce	*WHSV = 24000 ml* g_{cat}^{-1} h^{-1} $C_2H_6:CO_2$ *= 1:2* *T = 650 ℃ (10 h)*	$X_{C_2H_6}$ = 70% X_{CO_2} = 71% S_{CO} = 97%	*[18]*

*In the introduction section, a relevant discussion on the two reactions occurring and the most prominent relevant literature works has been provided. "A common application for this variation ... favoring C-H bond scission and ethane dehydrogenation." (**page 2-3**). In the catalytic experiments section, the discussion on relevant literature works has also been improved, and a comparison Table has also been included in the **Supplementary Table 2**, where the catalytic performance of R400 and R850 catalysts (prepared from the same material via different exsolution treatment) is compared to other catalytic materials reported in the literature, for the respective reactions (CO_2-assisted oxidative ethane dehydrogenation and dry ethane reforming). As such, corresponded discussion part is added in the main text (**page 12**)*

Please note, that relevant changes have been made in the revised manuscript, and supplementary information file with red font.

References

[1] B. Yan, et al., Proc. Natl. Acad. Sci., 2018, 115(12), 2902-2907.

[2] Z. Xie, et al., Chem, 2020, 6(10), 2703–2716.

[3] M. Guo, et al., ChemCatChem, 2021, 13(13), 3119-3131.

[4] X. Li, et al., Fuel, 2022, 322, 124122.

[5] L. Li, et al., Appl. Catal. A: Gen., 2022, 635, 118565.

[6] M. Tasioula, et al., ACS Catal., 2023, 13(4), 2176–2189.

[7] Y. Zheng, et al., ACS Catal., 2023, 14(7), 11153−11163.

[8] X. Li, et al., Int. J. Hydrogen Energy., 2024, 79, 45–59.

[9] X. Li, et al., Chem. Eng. J., 2024, 484, 149047.

[10] Q. Du, et al., Carbon Capture Sci. Technol., 2025, 14, 100327.

[11] B. Zhao, et al., J. Catal., 2018, 358, 168–178.

[12] Z. Xie, et al., Appl. Catal. B: Environ., 2019, 245, 376–388.

[13] K. H. Kim, et al., J. CO2 Util., 2021, 53, 101713.

[14] A. I. Tsiotsias, et al., J. CO2 Util., 2022, 61, 102046.

[15] T. Zhang, et al., Engineering., 2022, 18, 173–185.

[16] Y. Wang, et al., Appl. Catal. B: Environ., 2023, 337, 123010.

[17] C. Wang, et al., Chem. Eng. J., 2024, 491, 152091.

[18] X. Chen, et al., Int. J. Hydrogen Energy., 2025, 103, 501–512.

Comment 1 of Reviewer 2: The reaction evaluation, which should form the basis for understanding structure–function relationships, is underdeveloped. The authors primarily link catalytic behavior to exposure and conversion rather than through mechanistic insights. The treatment of selectivity is superficial, lacking a detailed breakdown of product distribution or reaction pathways. Without a thorough kinetic or mechanistic discussion, the proposed correlation between structural states and catalytic performance lacks depth and robustness. Key reaction parameters such as activation energy, turnover frequency, and product-specific rates are absent, further limiting interpretability. The dynamic nature of the reaction conditions, particularly the linear temperature ramp toward steady state, presents an opportunity for extracting mechanistic insights. The manuscript does not fully exploit this aspect. Comparing catalyst behavior at equivalent conversion levels during the transient phase could reveal whether the observed performance differences stem from intrinsic activity or changes in redox state. Such analysis is absent, leaving ambiguity about whether the differences between catalysts are kinetic, structural, or purely linked to the accessible number of sites.

We thank the Reviewer for his/her valuable comment. In agreement with the Reviewer's

suggestions, we proceeded to make several significant changes, conducted additional catalytic experiments, and greatly expanded the discussion regarding the catalytic evaluation section of our Manuscript. The relevant changes made are the following:

*First of all, we proceeded to calculate the reactant conversion rates and the product formation rates/ space time yields (STY) based on the values at the end of the catalytic tests in Figure 6. These have been included in a new Table presented in the Supporting Information (**Table R2**). Regarding ethane dehydrogenation (ODH), a comparison with the literature can be made by utilizing the work of Qiang et al. [19], where it can be concluded that R850, with a STY for C_2H_4 at 692 μmol g_{cat}^{-1} min^{-1} has a quite high C_2H_4 production compared to the other literature works, that is only slightly smaller to the higher value obtained in their own work at 700 °C (798 μmol g_{cat}^{-1} min^{-1}).*

***Table R2.** Reactant conversion and product formation rates following 11 hours of reaction, i.e., 3 h of temperature ramping (400 to 700 °C) and 8 h of time on stream (700 °C constant).*

**Material**	**Reactant conversion rate (μmol g_{cat}^{-1} min^{-1})**		**Product formation rate/ Space time yield (STY) (μmol g_{cat}^{-1} min^{-1})**		
	**C_2H_6**	**CO_2**	**C_2H_4**	**CO**	**H_2**
R400	1357	2817	283	3650	2163
R850	921	1260	692	1513	750
R850-O-R400	995	1545	463	1738	963
R400-O-R850	870	1200	579	1375	713

***Regarding the reaction mechanisms for ODH and DER**, we kindly note that there are quite many already published works in the literature that have studied the reaction mechanism and the reaction intermediates for similar materials (Fe-Ni based) in detail using several techniques (incl. in-situ DRIFTS, pulse experiments, and DFT calculations). A quite informative and representative reaction scheme has been presented in the work of Xie et al. [20]:*

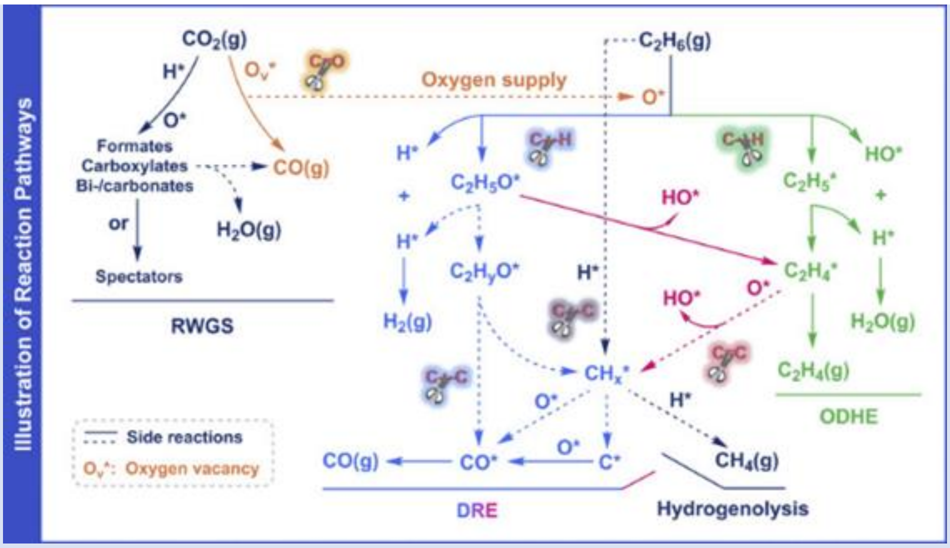

Figure R1. *Schematic illustration of potential reaction pathways for the reaction of CO_2 and C_2H_6 [20].*

In short, CO_2 is commonly reported to be activated to CO and reactive oxygen species (O) on the support, the metal-support interface, or also on the FeO_x overlayer, and then O* species are utilized for the ethane activation part. For ODH (or ODHE), ethane is activated to $C_2H_{5(ads)}$, then to $C_2H_{4(ads)}$ (both indicated as C_2H_x in* ***Figure 8****), which eventually desorbs to $C_2H_{4(g)}$, i.e., the C-C bond is preserved, and only C-H bonds are cleaved. For DER (or DRE), potential adsorbed intermediate species include $C_2H_{x(ads)}$, $C_2H_xO_{(ads)}$, and $CH_{x(ads)}$, which, following various oxidation steps, are eventually oxidized to $CO_{(g)}$.*

*We kindly note that we have provided a rather lengthy discussion in the Manuscript regarding the possible reaction mechanisms during ODH and DER based on prior literature works, as well as a relevant Figure (****Figure 8****). We also note that we proceeded to perform some important revisions in the relevant discussion part, and on* ***Figure 9****. The current manuscript focuses heavily on the catalyst characterization part, the material structural transformations occurring during different exsolution/ oxidation/ regeneration treatments, and how these can lead to selectivity switching between ODH and DER over a single multifunctional perovskite material. Therefore, we kindly note, that an additional detailed mechanistic or kinetic investigation (via e.g., in-situ DRIFTS, DFT analysis, dedicated kinetic experiments etc.), such as those presented in many prior works (please refer to the references cited in the Manuscript during the relevant discussion) would deviate from the main scope of the current work.*

However, in agreement with the Reviewer's suggestion, we did proceed to conduct additional experiments regarding the calculation of key reaction parameters, such as the activation energy values, and to compare catalyst behavior at (almost) equivalent conversion levels. Since the obtained data points during the transient phase of the experiments in Figure 6 were not enough to calculate these parameters we proceeded to conduct additional dedicated experiments with more data points during the temperature ramp (10 °C step in the 560 – 660 °C range), in order to be able to more accurately calculate the reaction rates at lower conversions, the activation energy values, and to better compare the catalytic activity and selectivity at almost equivalent C_2H_6 *conversions. The results are now presented as* **Supplementary Fig. 11, 12, and Supplementary Table 3 and 4.**

Figure R2. *displays the Arrhenius plots along with small insets for the reactant conversion/ product-specific formation rates.* ***Table R3*** *lists the activation energy values for* C_2H_6 *and* CO_2 *conversion, as well as CO and* C_2H_4 *production, obtained from the Arrhenius plots. These activation energy values are in agreement with those reported in other literature works [21-28]. In all of the materials, the* CO_2 *activation energy values are lower than those for* C_2H_6 *activation, which can be ascribed to the concurrent side-reaction of reverse water-gas shift (RWGS) [21,24]. The activation energy for CO production generally follows the trend of* CO_2 *and* C_2H_6 *conversion, whereas for* C_2H_4 *production, the values are quite higher, even for the dehydrogenation-selective materials. By comparing the different materials, R400 has the lowest activation energy values, due to its significantly pronounced low-temperature catalytic activity (as also shown in* **Figure** *7). On the contrary, the activation energy values are the highest for R850, whereas the regenerated samples (R850-O-R400 and R400-O-R850) show intermediate values. Please note, that we did not proceed to calculate the turnover frequency (TOF) values due to the following reasons: i) The catalyst structure is highly complicated with different metallic and oxidic phases, meaning that there are also likely different active sites for* C_2H_6 *and* CO_2 *activation, ii) there is a high degree of uncertainty on the precise amount of Ni and Fe residing on the surface compared to the amount in the bulk, and iii) probing the active sites with typical gas chemisorption (e.g., CO) does not take into account the sites in the* FeO_x *overlayer, which are active for dehydrogenation (ODH).*

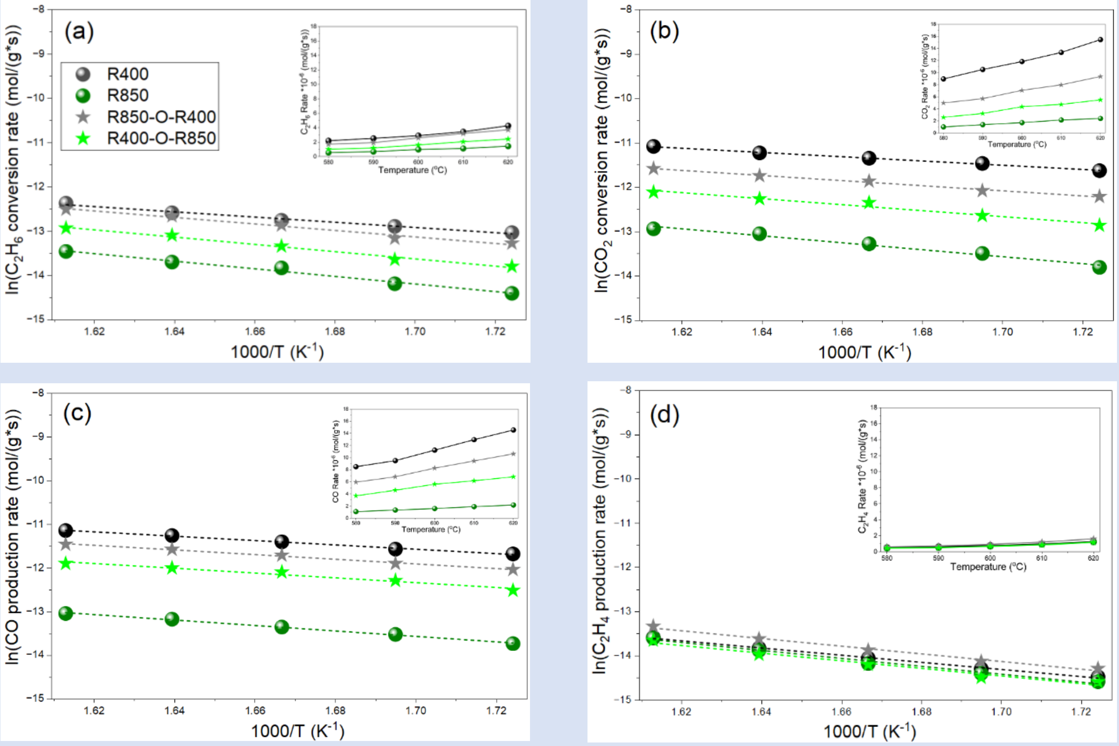

Figure R2. *Arrhenius plots for the calculation of the apparent activation energies: Natural logarithm of the consumption rates of C_2H_6 (a) and CO_2 (b), and the production rates of CO (c) and C_2H_4 (d) versus reciprocal temperature. The corresponding rates versus temperature plots are presented as insets.*

Table R3. *Activation energy values for C_2H_6 and CO_2 conversion, as well as CO and C_2H_4 production, calculated via the Arrhenius plots.*

Material	$E_a(C_2H_6)$	$E_a(CO_2)$	$E_a(CO)$	$E_a(C_2H_4)$
R400	104	84	88	143
R850	151	138	110	158
R850-O-R400	129	101	94	153
R400-O-R850	144	119	97	152

*Additionally, in agreement with the Reviewer's suggestion, a comparison is made between the different materials at almost equivalent C_2H_6 conversion levels (7-8% C_2H_6 conversion values, **Figure R3**) based on these new catalytic experiments (necessary to achieve similar C_2H_6 conversion values for all 4 materials at slightly different temperature). As shown in Figure S10, the CO_2 conversion varies greatly within the materials, as do the ethane-basis selectivity values for CO (reforming, DER) and C_2H_4 (dehydrogenation, ODH). In particular, R400 still shows the highest DER selectivity and R850 the highest ODH selectivity, with the regenerated materials falling in between (particularly R850-O-R400, due to the incomplete*

*regeneration of the initial perovskite structure during re-oxidation). This verifies that the observed catalytic performance (activity and selectivity) differences over the 4 different materials obtained from the same parent perovskite, LSTFN, stem from the intrinsic catalytic activity (kinetics) [29]. A similar comparison at comparable C_2H_6 conversion levels, showing opposing selectivity values for CO (reforming) and C_2H_4 (dehydrogenation) is also reported in Ref. [29] from the Chen group. In their case, these opposing selectivity values are achieved via preparing two different materials with different Ni:Fe ratios over a CeO_2 support. As such, a relevant discussion on the reaction mechanism has been provided in the main text (**page 12-14**)*

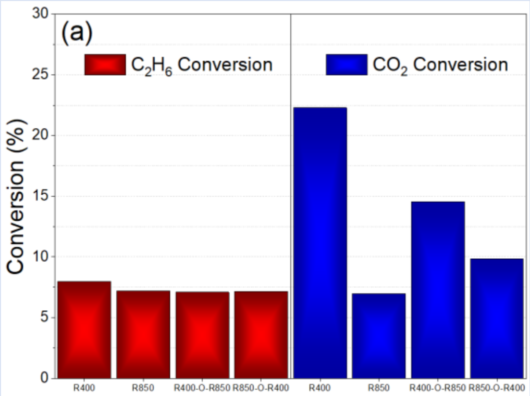

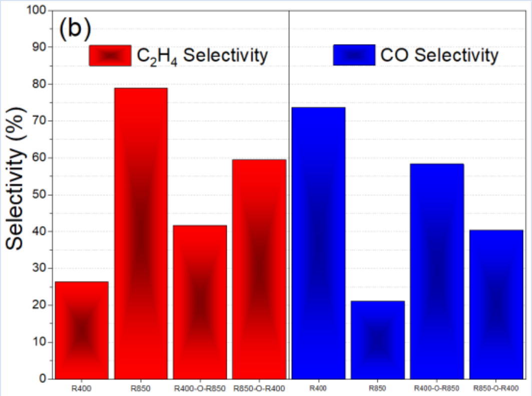

***Figure R3**. Comparison of C_2H_6 and CO_2 conversions (a), as well as C_2H_4 and CO selectivities (b) at similar C_2H_6 conversion levels (7-8%).*

Please note, that relevant changes have been made in the revised manuscript, and supplementary information file with red font.

References:

[19] W. Qiang, et al., Chinese J. Catal., 2025, 70, 272–284.

[20] Z. Xie, et al., Matter, 2021, 4(2), 408–440.

[21] Z. Xie, et al., Appl. Catal. B: Environ., 2019, 245, 376–388.

[22] Y. Wang, et al., Appl. Catal. B: Environ., 2023, 337, 123010.

[23] C. Wang, et al., Chem. Eng. J., 2024, 491, 152091.

[24] X. Chen, et al., Int. J. Hydrogen Energy, 2025, 103, 501–512.

[25] A. I. Tsiotsias, et al., ACS Nano, 2022, 16(6), 8904–8916.

[26] F. Cancino-Trejo, et al., Chem. Eng. J. Adv., 2022, 12, 100404.

[27] M. Guo, et al., ChemCatChem, 2021, 13(13), 3119–3131.

[28] X. Li, et al., Int. J. Hydrogen Energy, 2024, 79, 45–59.

[29] B. Yan, et al., Proc. Natl. Acad. Sci., 2018, 115(33), 8278–8283.

Comment 3 of Reviewer 2: The RX-O-RY sample, which appears to have undergone a reduction-oxidation-reduction treatment, is not described clearly. It is implied that this sequence mimics potential in situ redox cycles during catalytic operation. However, the paper does not explore whether these transformations can occur under actual reaction conditions, such as in the presence of ethylene or CO_2. The lack of post-reaction characterization or detailed in situ monitoring under working conditions makes it difficult to establish whether the proposed redox cycling mechanism is relevant during catalysis. The stability and reversibility of the transformations under reaction conditions are not adequately supported.

We thank the Reviewer for his/her valuable comment. ***In agreement with the Reviewer's suggestion****, we proceeded to perform* ***in situ XRD characterization*** *following oxidation and reduction (exsolution) treatments,* ***additional catalytic experiments*** *(temperature ramp + time-on-stream)* ***with in-situ intermediate oxidation and reduction (exsolution) treatments****,* ***Atomic resolution-TEM analysis*** *as well as a* ***deeper post-reaction characterization on the spent catalyst materials****.*

To ease the reviewer's concerns on the actual surface transformation occurring on one single system we applied for beamtime at the synchrotron facility DESY, Hamburg and successfully performed in situ XRD during reduction and oxidation cycles on the LSTFN2 material at the

P08 beamline. We kindly ask to consider that due to safety restriction of the synchrotron facility, it was not possible to carry out in situ catalysis experiments.
*We performed two set of measurements mimicking the redox cycles of R400-O-R850 and R850-O-R400 on LSTFN2 (**Figure R4**). The exsolution and redissolution procedures of LSTFN2 powder sample were tracked following two protocols: 1) (**Figure R5a**) The sample was heated to 850 °C under synthetic air (10 mL min^{-1}) with a heating rate of 10 °C min^{-1}. Once the temperature was reached, the atmosphere was switched to 4% H_2 in N_2. The gas flow was initially 10 mL min^{-1} for ~20 minutes and then increased to 20 mL min^{-1} for a 3-hour reduction period. The gas was then switched back to synthetic air (20 mL min^{-1}) for a 5-hour of re-oxidation at 850 °C. Finally, the sample was cooled to 400 °C at 10 °C min^{-1} in air and subjected to a second reduction in 4% H_2 in N_2 (20 mL min^{-1}) for 3 hours before cooling to room temperature. 2) (**Figure R5b**) The sample was heated to 400 °C in synthetic air (20 mL min^{-1}) with 10 °C min^{-1}. At 400 °C, the gas was switched to 4% H_2 in N_2 (20 mL min^{-1}) for a 3-hour reduction. The atmosphere was then switched back to air (20 mL min^{-1}), and the temperature was raised to 850 °C for a 5-hour re-oxidation. Finally, while maintaining 850 °C, the gas was switched to 4% H_2 in N_2 (20 mL min^{-1}) for an additional 3-hour reduction.*

*The obtained results are in full agreement with the ex situ characterization shown in the manuscript. The XRD data in **Figures R5** reveal significant structural changes during reduction at 850 °C under H_2. The emergence of a new peak at Q = 3.0 $Å^{-1}$, coupled with a pronounced intensity increase at Q = 3.5 and 4.9 $Å^{-1}$, is attributed to the formation of $Fe_{0.64}Ni_{0.36}$ alloy nanoparticles. The concurrent decay of the peak at ~2.5 $Å^{-1}$ confirms the depletion of the $NiFe_2O_4$ phase. Notably, exsolution was triggered consistently upon exposure to 850 °C in H_2, independent of the sample's thermal and redox history. The reversibility of this process was confirmed by the redissolution of the nanoparticles upon removal of either the reducing atmosphere or the high temperature, indicating a reversible transformation between a metallic $Fe_{0.64}Ni_{0.36}$ alloy and a reincorporated oxide phase.*

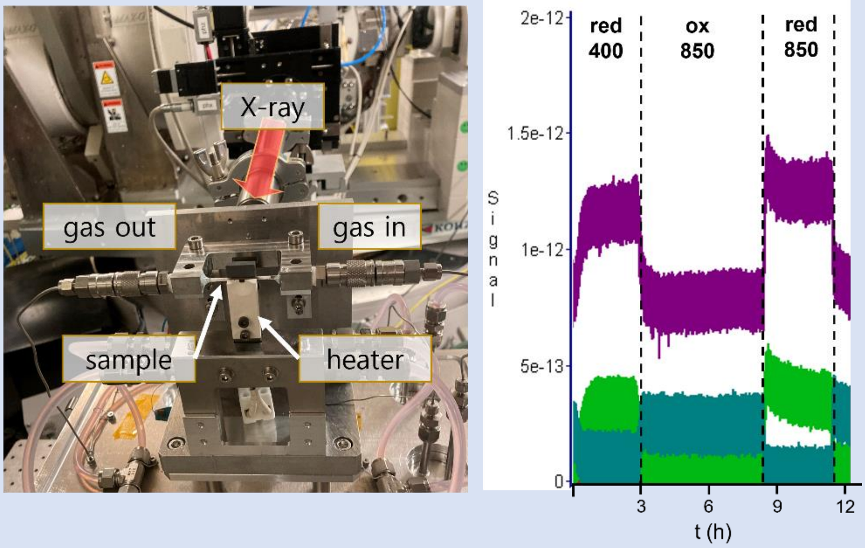

Figure R4. *Picture of the high-temperature in situ cell (left) and gas profile of the mass spectrometer during the R400-O-R850 redox cycle (right). The purple curve indicates the concentration of N_2, the light green indicates H_2 and the dark green curve indicates the signal of O_2.*

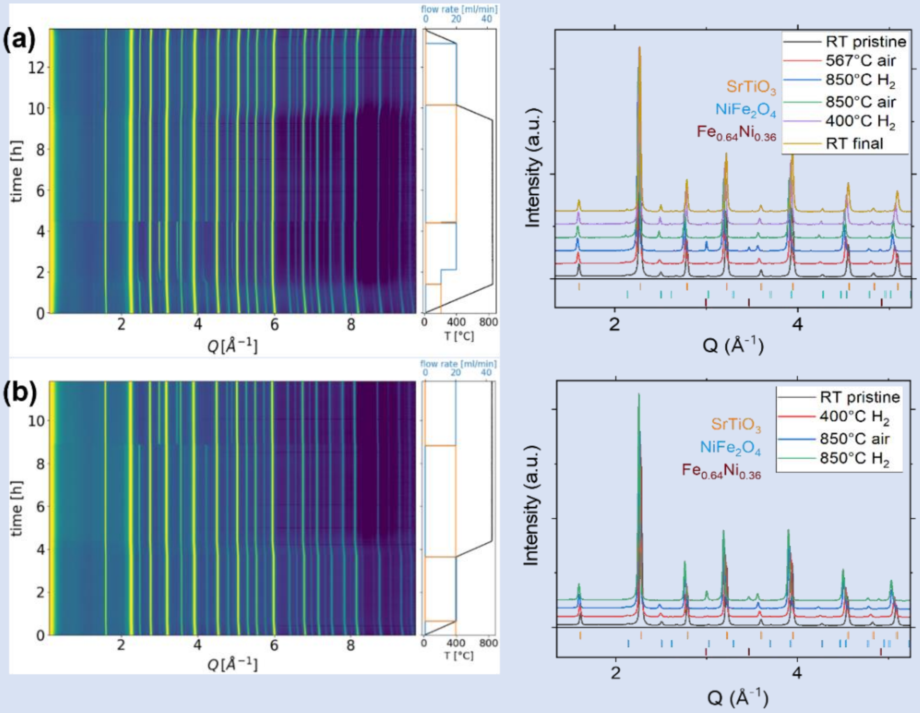

Figure R5. *In situ XRD patterns during the R850-O-R400 (a) and the R400-O-R850 (b) redox cycles of LSTN2 along with the respective XRD curves at each redox stage. The flow rate of air and H_2 in N_2 is shown in orange and blue, respectively. The temperature profile is marked in black.*

*To further evaluate whether the catalytic performance/ selectivity switch (and thus the corresponding material structural transformations) can occur in-situ, i.e., in the catalytic evaluation setup, a new, cyclic, catalytic experiment was designed, which consisted of 3 catalytic cycles with intermediate in-situ oxidation and reduction treatment steps. As described in the experimental section (**page 17 of main text**), we started with the R400 material (previously exsolved at 400 ℃) and performed the same procedure of temperature ramping (400 to 700 ℃) and time-on-stream at 700 ℃, as in **Figure R6** (albeit with a reduced time-on-stream duration of 4 h); this step being denoted as Cycle 1. Afterwards, in-situ oxidation in the catalytic reactor setup was performed at 850 ℃ by flowing 20% O_2 in Ar, followed by in-situ reduction at 850 ℃ under 5% H_2 in Ar, in order to mimic the oxidation and reduction treatments performed ex-situ for R400-O-R850. The material was then evaluated again via the same catalytic procedure (Cycle 2), followed again by in-situ oxidation (850 ℃) and reduction (400 ℃), in order to attempt a reversal to the catalytic performance of R400, and another catalytic cycle followed (Cycle 3). The results are presented in **Figure 9** in the Revised Manuscript.*

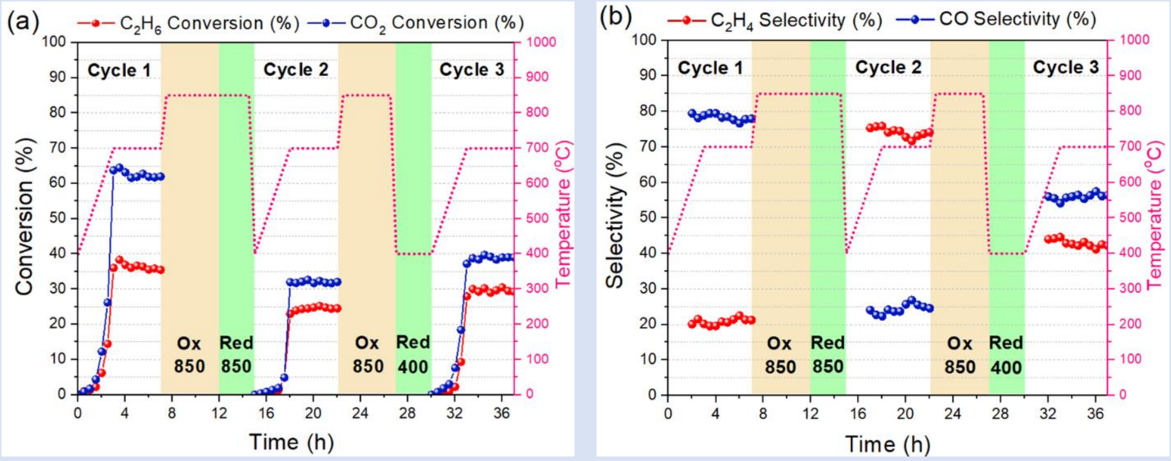

***Figure R6.** Trend over time for the (a) C_2H_6 and CO_2 conversions, as well as (b) C_2H_4 and CO selectivities during the catalytic testing for 3 cycles (temperature ramping from 400 to 700 ℃ and time-on-stream at 700 ℃ constant) with intermediate in-situ oxidation and reduction steps at either 850 or 400 ℃.*

*Based on **Figure R6**, it is clear that the first catalytic cycle (Cycle 1) for the R400 material proceeds similarly to the experiment in Figure 6, namely leading to relatively high C_2H_6 and*

*CO_2 conversions, which increase with temperature and remain relatively stable at 700 °C with a small degree of deactivation. The R400 material is quite selective towards dry ethane reforming (DER), with approx. 78% CO selectivity. Following in-situ oxidation and reduction at 850 °C, a dramatic shift in catalytic performance during Cycle 2 is observed. On the one hand, lower C_2H_6 and CO_2 conversion values are reached at 700 °C, which however remain quite stable, but on the other hand, the reaction selectivity is completely switched in favor of oxidative ethane dehydrogenation (ODH), with C_2H_4 selectivity of approx. 75%. This catalytic behavior is quite similar to R400-O-R850, with the corresponding ex-situ oxidation/ reduction treatments, but without undergoing prior catalytic operation, and even to the freshly exsolved R850 material (**Figure 7 of main text**). Afterwards, during Cycle 3, and following in-situ oxidation at 850 °C and in-situ reduction at 400 °C, an attempt is made to revert the catalytic activity and selectivity back to the initial one of R400 (Cycle 1). However, the C_2H_6 and CO_2 conversion values are stabilized at lower levels than during Cycle 1 (though higher than during Cycle 2), and the selectivity is rather intermediate between dry reforming and dehydrogenation (though slightly favouring dry reforming), at approx. 56% CO selectivity vs 42% C_2H_4 selectivity (a small amount of CH_4 is also produced in all materials). This means that the catalytic performance and selectivity switch from dehydrogenation to reforming is only partial. This is also reflected however on the initial catalytic performance results on Figure 6 for R850-O-R400, as well as on the material characterization results of R850-O-R400, where the initial state of R400 could not be completely regenerated.*

Therefore, although opposite ethane reforming/dehydrogenation selectivities following different exsolution treatments over the same perovskite starting material, and even efficient selectivity switch from reforming to dehydrogenation following in-situ treatments, were achieved, future research work needs to be directed on the complete structural regeneration of the initial perovskite structure, in order to achieve complete selectivity switching during multi-cyclic operation.

*In addition, we conducted HAADF-STEM, EDX, and EELS analysis on the post-catalysis (spent) samples R400, R850, R850-O-R400, and R400-O-R850 to study potential material nanostructure changes of the spent catalysts following the catalytic testing procedure presented in **Figure R6** (temperature ramping and time-on-stream at 700 ^oC) We also wanted*

to observe whether the outmost FeO_x layer is maintained or altered during the catalytic operation.

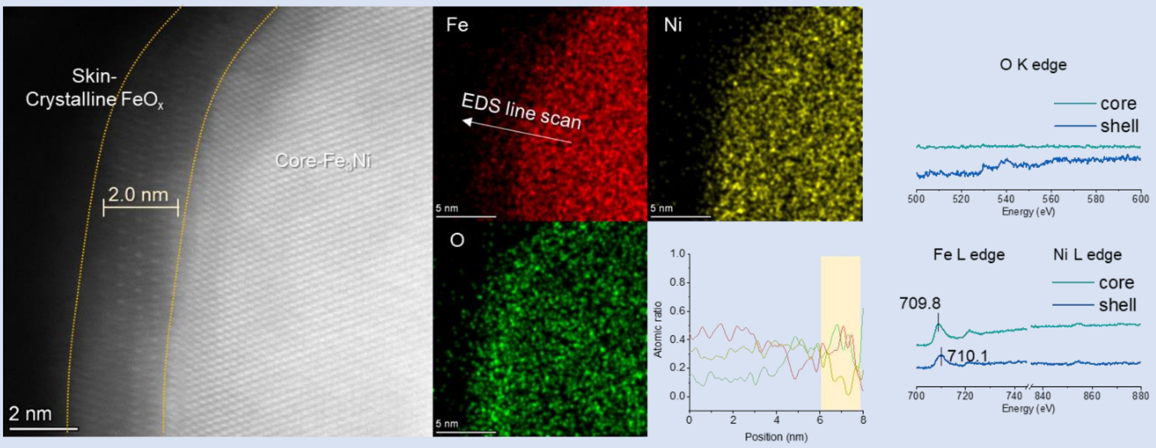

Figure R7. *HADDF-STEM images and corresponding EDX and EELS results on spent R850 (post-catalysis).*

Figure R7 *represents the exsolved nanoparticle (representative image) for spent R850 after the catalytic reaction of temperature ramping and time-on-stream, which clearly shows the intact crystalline FeO_x overlayer (also present in the fresh/exsolved sample) even after the prolonged reaction operation. The enrichment of iron species was observed in the line scan area (~ 2 nm), with less abundant Ni. The EELS result further reveals that the outermost layer is composed of iron oxide (710.1 eV), with distinct differences in core metals. In the case of the spent R400, the outermost FeO_x layer presents an amorphous-like state (Figure R8), which is attributed to ex-situ air exposure during the handling process (similarly to fresh/ exsolved R400). In the O K edge, it reveals broader peak intensity compared to the R850, indicative of less hybridization between Fe and O, presumably resulting from native oxide [30, 31]. Thus, it is likely to note that metallic FeNi nanoparticles are the major active species for R400, while FeNi nanoparticles encapsulated by FeO_x overlayer are the major active species for R850, and that the reaction conditions do not alter these initial active species any further.*

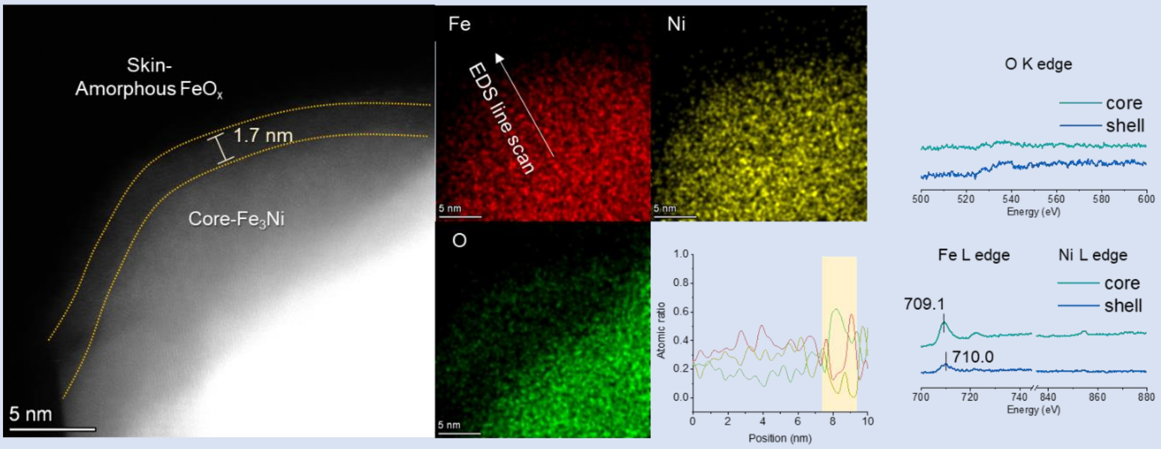

Figure R8. *HADDF-STEM images and corresponding EDX and EELS results on spent R400 (post-catalysis).*

We further investigate the crystallinity of the outermost layer for the redox-treated (regenerated) spent samples (R400-O-R850 and R850-O-R400). As can be seen in the TEM images of ***Figure R9****, the active species vary depending on the final reduction treatment. For R400-O-R850, the outermost layer presents crystallinity with a relatively well-resolved O K edge spectrum in EELS, while the R850-O-R400 shows an amorphous* FeO_x *layer similar to what we've observed in reduced samples, and these are well coincident with our prior in situ XRD results. We would like to note that they are maintained during catalysis when compared to the corresponding fresh/ exsolved samples, as in the cases of R400 and R850, owing to the high structural stability of all the perovskite-derived material used in this study.*

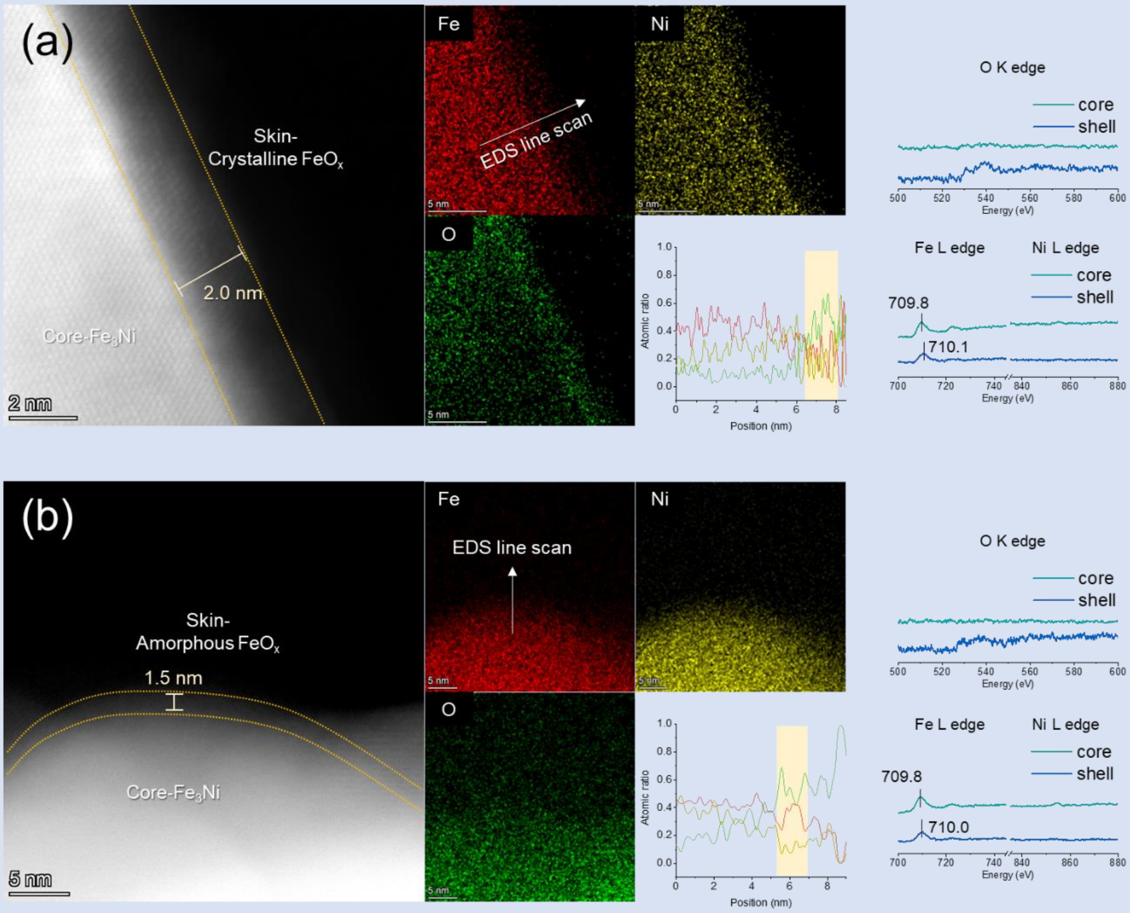

Figure R9. *HADDF-STEM images and corresponding EDX and EELS results on spent (a) R400-O-R850, and (b) R850-O-R400 (post-catalysis).*

As such, a relevant discussion on the reaction mechanism has been provided in the main text ***(page 9, 12-15, Figure 6, 9, 10, Supplementary Fig. 9, and 20)***

Please note, that relevant changes have been made in the revised manuscript, and supplementary information file with red font.

<u>References:</u>

[30] C. Wang, et al., J. Am. Chem. Soc., 2009, 131(25), 8824-8832.

[31] C. H. Park, et al., Adv. Mater., 2024, 36(28), 2403392.

Comment 4 of Reviewer 2: Furthermore, the literature context is insufficiently developed. The title and abstract are very vague.

We thank the Reviewer for his/her valuable comment. In agreement with the Reviewer's suggestion, we have proceeded to expand and improve the literature context, as described in our previous answer to the first comment (Comment #1). Furthermore, as per the Reviewer's suggestion, we also proceeded to revise the title and the abstract. Please refer to the Revised version as below:

Title: Shapeshifting exsolved FeNi bimetallic nanostructures as catalytic switchers during the CO2-mediated ethane conversion

Abstract: In the era of the energy transition, the development of sustainable, performing, and multifunctional catalysts that adapt to complex catalytic processes is essential. Herein we present the development of shapeshifting bimetallic FeNi catalysts based on the exsolution approach for the CO_2*-mediated ethane conversion.* Fe_3Ni *(3 nm) and* $FeO_x@Fe_3Ni$ *(40 nm) exsolved nanoparticles were obtained from a* $La_{0.4}Sr_{0.4}Ti_{0.6}Fe_{0.35}Ni_{0.05}O_{3-\delta}$ *(LSTFN) parent perovskite when exposed to a reducing atmosphere at 400 and 850 °C, respectively. The formation of the core-shell system was analyzed by in situ TEM, Mössbauer and X-ray absorption spectroscopy and ascribed to the strong metal-support interactions. Mediated by the oxidative regeneration of the perovskite host, a shapeshift between the two different nanoparticle structures in the same perovskite parent material was realized to a great extent by a secondary reduction (exsolution) treatment at the opposite temperature. Catalytic tests showed that the materials with supported* Fe_3Ni *and* $FeOx@Fe_3Ni$ *nanostructures (exsolved at 400 and 850 °C) were stable and selective by approx. 80% towards the ethane dry reforming and the* CO_2 *assisted ethane oxidative dehydrogenation pathway, respectively, i.e., achieving opposite catalytic selectivity via simply treating the same parent material at different exsolution temperatures. Furthermore, upon regeneration treatment and nanoparticle shapeshifting, the catalytic selectivity was switched to a great extent. This trend was also observed during in situ regeneration (oxidation/ reduction) in a multi cyclic operation, thus showing that exsolution offers a novel and promising strategy for the design of modern, multifunctional catalyst materials with switching catalytic performance.*

Reviewer #3

I co-reviewed this manuscript with one of the reviewers who provided the listed reports. This is part of the Nature Communications initiative to facilitate training in peer review and to provide appropriate recognition for Early Career Researchers who co-review manuscripts.
